# Garsorasib, a KRAS G12C inhibitor, with or without cetuximab, an EGFR antibody, in colorectal cancer cohorts of a phase II trial in advanced solid tumors with KRAS G12C mutation

**DOI:** 10.1038/s41392-025-02274-z

**Published:** 2025-06-17

**Authors:** Dan-Yun Ruan, Hao-Xiang Wu, Ye Xu, Pamela N. Munster, Yanhong Deng, Gary Richardson, Dong Yan, Myung-Ah Lee, Keun-Wook Lee, Hongming Pan, Steven Hager, Xingya Li, Shaozhong Wei, Xinfang Hou, Craig Underhill, Michael Millward, Ina Nordman, Jingdong Zhang, Jianzhen Shan, Guohong Han, Jaspreet Grewal, Shirish M. Gadgeel, Rachel E. Sanborn, Seok Jae Huh, Xiaohua Hu, Yihong Zhang, Ziyong Xiang, Laisheng Luo, Xiaoxi Xie, Zhe Shi, Yaolin Wang, Ling Zhang, Feng Wang, Rui-Hua Xu

**Affiliations:** 1 Department of Clinical Research, Sun Yat-sen University Cancer Center, State Key Laboratory of Oncology in South China, Guangdong Provincial Clinical Research Center for Cancer, Sun Yat-sen University, Guangzhou, China; 2 Research Unit of Precision Diagnosis and Treatment for Gastrointestinal Cancer, Chinese Academy of Medical Sciences, Guangzhou, China; 3 Department of Colorectal Surgery, Fudan University Shanghai Cancer Center, Shanghai, China; 4 Department of Medicine, University of California San Francisco, San Francisco, CA USA; 5 Department of Medical Oncology, The Sixth Affiliated Hospital, Sun Yat-sen University, Guangzhou, China; 6 Department of Medical Oncology, Cabrini Hospital - Malvern, Malvern, VIC Australia; 7 Department of Oncology, Beijing Luhe Hospital Affiliated to Capital Medical University, Beijing, China; 8 Division of Medical Oncology, Department of Internal Medicine, Seoul St. Mary’s Hospital, College of Medicine, Cancer Research Institute, The Catholic University of Korea, Seoul, South Korea; 9 Seoul National University College of Medicine, Seoul National University Bundang Hospital, Seongnam, Republic of Korea; 10 Department of Medical Oncology, Sir Run Run Shaw Hospital, Zhejiang University School of Medicine, Hangzhou, China; 11 Medical Oncology Hematology, California Cancer Associates for Research and Excellence, Inc. (cCARE), Fresno, CA USA; 12 Department of Medical Oncology, The First Affiliated Hospital of Zhengzhou University, Zhengzhou, China; 13 Department of Gastrointestinal Oncology Surgery, Hubei Cancer Hospital, Wuhan, China; 14 Department of Medical Oncology, Henan Cancer Hospital, Zhengzhou, China; 15 Border Medical Oncology Research Unit, Albury Wodonga Regional Cancer Centre, Albury, NSW Australia; 16 Linear Clinical Research & University of Western Australia, Perth, WA Australia; 17 Medical Oncology Department, Calvary Mater Newcastle, Waratah, NSW Australia; 18 Medical Oncology Department of Gastrointestinal Cancer, Liaoning Cancer Hospital & Institute, Shenyang, China; 19 Department of Medical Oncology, The First Affiliated Hospital of Zhejiang University School of Medicine, Hangzhou, China; 20 Department of Liver Diseases and Digestive Interventional Radiology, Xi’an International Medical Center Hospital, Xi’an, China; 21 Medical Oncology Hematology, Norton Cancer Institute, Louisville, KY USA; 22 Division of Hematology and Oncology, Department of Internal Medicine, Henry Ford Cancer Institute/Henry Ford Health System, Detroit, MI USA; 23 Medical Oncology Department, Earle A. Chiles Research Institute, Providence Cancer Institute, Portland, OR USA; 24 Division of Hematology-Oncology, Department of Internal Medicine, Dong-A University College of Medicine, Busan, South Korea; 25 Department of Medical Oncology, The First Affiliated Hospital of Guangxi Medical University, Nanning, China; 26 InventisBio Co. Ltd, Shanghai, China; 27 Department of Medical Oncology, Sun Yat-sen University Cancer Center, State Key Laboratory of Oncology in South China, Guangdong Provincial Clinical Research Center for Cancer, Sun Yat-sen University, Guangzhou, China

**Keywords:** Clinical trials, Gastrointestinal cancer, Drug development

## Abstract

Mutations in the KRAS gene have long been implicated in the pathogenesis of colorectal cancer (CRC). KRAS G12C inhibitors overcome the “undruggable” challenge, enabling precision therapy. Garsorasib (D-1553), a highly potent and selective KRAS G12C inhibitor, has demonstrated promising anti-tumor activity and favorable safety profile in early clinical trials. We conducted an open-label, nonrandomized phase II trial (ClinicalTrials.gov, NCT04585035) to assess the safety and efficacy of garsorasib with or without cetuximab in KRAS G12C-mutated CRC. In the monotherapy cohort (n = 26), objective response rate (ORR) was 19.2% (95% CI, 6.6–39.4), disease control rate (DCR) was 92.3% (95% CI, 74.9–99.1), median progression-free survival (PFS) was 5.5 months (95% CI, 2.9–11.6) and median overall survival (OS) was 13.1 months (95% CI, 9.5-NE). In the combination cohort (n = 42), ORR was 45.2% (95% CI, 29.8–61.3), DCR was 92.9% (95% CI, 80.5–98.5), median PFS was 7.5 months (95% CI, 5.5–8.1), and median OS was not reached. Grade ≥3 treatment-related adverse events occurred in 5 (19.2%) and 6 (14.3%) patients in monotherapy and combination cohort, respectively. Garsorasib with or without cetuximab showed a promising efficacy and manageable safety profiles in heavily pretreated patients with KRAS G12C-mutated CRC, providing a potential new treatment approach for such population.

## Introduction

Colorectal cancer (CRC) accounts for approximately 10% of all cancer cases diagnosed annually, and remains as the second leading cause of cancer-related deaths worldwide.^1^ Metastatic progression occurs in over 50% of CRC patients during their disease course, rendering metastatic colorectal cancer (mCRC) a persistent therapeutic challenge. While highly-selected oligometastatic cases may achieve durable remission through multidisciplinary interventions including metastasectomy, the majority of mCRC patients are incurable. For this population, systemic therapies combining cytotoxic agents with targeted therapies, such as anti- epidermal growth factor receptor (EGFR) therapy, constitute the cornerstone of survival prolongation. However, KRAS (Kirsten rat sarcoma viral oncogene homolog) mutations occur in approximately 40% of CRC cases and confer resistance to anti-EGFR therapy. Clinical outcomes demonstrate marked inter-subtype heterogeneity among different KRAS variants, with particularly unfavorable prognostic implications observed in the KRAS G12C subtype. This specific mutation, present in ~3% of CRC cases,^2^ is associated with significantly reduced progression-free survival (PFS) and overall survival (OS) compared to KRAS wild-type tumors.^3–5^ The convergence of these poor survival outcomes with demonstrated refractoriness to conventional cytotoxic therapies and anti-EGFR regimens underscores an urgent need for innovative therapeutic strategies.

KRAS has retained its “undruggable” designation for decades, primarily attributed to two structural challenges.^6^ Firstly, the KRAS protein exhibits exceptionally high binding affinity for GTP, coupled with the abundant intracellular concentration of GTP, rendering the development of competitive inhibitors that effectively displace GTP-bound KRAS exceedingly challenging. Secondly, the absence of surface crevices suitable for small-molecule engagement defies traditional structure-based drug design paradigms. The era of targeting mutant KRAS was inaugurated in 2013 through seminal work by Ostrem and colleagues,^7^ which identified a druggable allosteric binding pocket adjacent to the switch-II region of the KRAS G12C mutant protein. Crucially, this switch-II pocket exhibits allele-specific accessibility—absent in both wild-type KRAS and other KRAS mutants—thereby enabling selective pharmacological targeting of the KRAS G12C oncoprotein. This discovery laid the foundation for structure-guided drug development, and direct KRAS G12C inhibition has become possible. In recent years, two KRAS G12C inhibitors, sotorasib and adagrasib, have been approved by the United States Food and Drug Administration (US FDA) for the treatment of patients with locally advanced or metastatic non-small-cell lung cancer (NSCLC),^8,9^ but not in mCRC as monotherapy as to date.

So far, the reported efficacies of KRAS G12C inhibitors monotherapy-exemplified by agents such as sotorasib, adagrasib, and divarasib-were less impressive in KRAS G12C-mutated CRC than those in NSCLC, with reported objective response rates (ORR) ranging from 9.7% to 29.1% and median progression-free survival (PFS) from 4 to 5.6 months.^10–12^ This intertumoral disparity suggests the involvement of CRC-specific resistance mechanisms, which is potentially driven by compensatory pathway activation. It has been reported that adaptive RAS-MAPK feedback reactivation occurred following KRAS G12C inhibition, and this reactivation may thus lead to KRAS G12C inhibition resistance.^13,14^ This adaptive feedback is mainly mediated by EGFR and its downstream signaling pathway in CRC. Accordingly, preclinical and clinical evidence suggests that dual targeting of KRAS G12C and EGFR could overcome this resistance mechanism.^14^ In the KRYSTAL-1 trial, an ORR of 34.0% was observed by the combination of adagrasib and cetuximab, which achieved a median PFS of 6.9 months.^15^ While in the phase III CodeBreaK 300 study, sotorasib plus panitumumab achieved a median PFS of 5.6 months and the hazard ratio for disease progression or death as compared with the standard-of-care group was 0.48.^16^ Based on these findings, FDA granted accelerated approval to adagrasib plus cetuximab on June 21, 2024, and approval to sotorasib plus panitumumab on January 16, 2025. However, Asian populations are significantly underrepresented in these two approved combination therapies, highlighting unmet clinical needs in this population.

Garsorasib is a potent small-molecule inhibitor of KRAS G12C that selectively and covalently binds to the switch-II pocket of KRAS G12C mutated protein in its inactive guanosine diphosphate-bound conformation to inhibit KRAS oncogenic signaling. Garsorasib has shown potent in vitro and in vivo anti-tumor activity in preclinical studies with high oral bioavailability and distribution to central nervous system tissues.^17^ Garsorasib 600 mg twice daily regimen can maintain garsorasib exposure above a target trough concentration throughout 24 hours and enable a sustainable inhibition of KRAS-dependent signaling for the duration of the dosing interval, which is predicted to maximize anti-tumor activity.^18,19^ This has been reflected in preliminary clinical data from garsorasib monotherapy which showed promising activity across several tumor types in patients with heavily pretreated KRAS G12C mutated cancer.^19–21^ As mentioned above, preclinical studies have suggested that a KRAS G12C inhibitor in combination with cetuximab, an EGFR-targeted antibody, could be an effective clinical strategy to overcome the resistance. Therefore, we conducted an open-label, nonrandomized phase II trial (ClinicalTrials.gov, NCT04585035) to assess the feasibility of garsorasib with or without cetuximab. Here, we present the safety and efficacy results from patients with KRAS G12C-mutated advanced or metastatic CRC receiving garsorasib alone or plus cetuximab in this trial.

## Results

### Patient disposition and characteristics

**Fig. 1:**
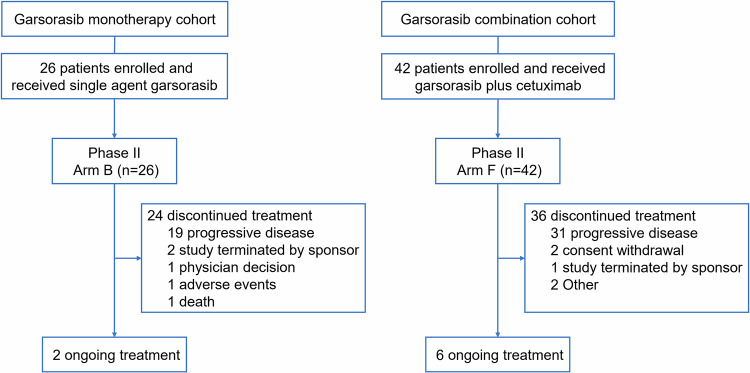
Study Flowchart

Between November 23, 2021 and June 29, 2023, a total of 26 patients with pretreated mCRC harboring KRAS G12C mutation were enrolled and received single-agent garsorasib treatment. The median duration of treatment was 6.0 months (range, 1.0–21.3), and the median follow-up was 13.0 months (range, 1.8–23.3) as data cut-off at February 29, 2024 (Supplementary Table 1). At the time of data cut-off, treatment was discontinued in 24 patients (92.3%), most of which were due to disease progression (Fig. 1). Between July 14, 2022 and May 10, 2023, 42 patients with pretreated mCRC harboring KRAS G12C mutation were enrolled and received garsorasib plus cetuximab, with median duration of treatment of 7.7 months (range, 0.9–18.0) and median follow-up of 13.0 months (range, 2.3–19.1) as data cut-off at February 29, 2024 (Supplementary Table 1). A total of 36 (85.7%) patients discontinued from study treatment, most of which were also due to disease progression (Fig. 1). The details of patient disposition are shown in Fig. 1.

Demographic and baseline characteristics are summarized in Table 1. In the garsorasib monotherapy cohort, the median age was 61.5 years (range, 44–75), and 38.5% of enrolled patients were female. In the combination cohort, the median age was 54 years (range, 32–76), and 38.1% of enrolled patients were female. The majority of the patients in both cohorts had previously received fluoropyrimidine, oxaliplatin or irinotecan-based chemotherapy as well as anti-VEGF therapy, with a median number of three prior lines of systematic therapy.

**Table 1:** Baseline demographics and clinical characteristics

	Monotherapy cohort (n = 26)	Combination cohort (n = 42)
Sex, n (%)		
Male	16 (61.5)	26 (61.9)
Female	10 (38.5)	16 (38.1)
Age (years)		
Median (range)	61.5 (44,75)	54.0 (32, 76)
Race, n (%)		
Asian	17 (65.4)	36 (85.7)
Hispanic	1 (3.8)	0
White	8 (30.8)	6 (14.3)
ECOG, n (%)		
0	11 (42.3)	13 (31.0)
1	15 (57.7)	29 (69.0)
Metastases, n (%)		
Yes	26 (100)	42 (100)
Metastases sites, n (%)		
Liver	17 (65.4)	32 (76.2)
Lung	12 (46.0)	18 (42.9)
Bone	6 (23.1)	2 (4.8)
Stage at study entry, n (%)		
IV	26 (100)	42 (100)
Primary tumor location, n (%)		
Left colon	13 (50.0)	15 (35.7)
Right colon	5 (19.2)	12 (28.6)
Rectum	8 (30.8)	15 (35.7)
Number of prior systematic treatment		
Median (range)	3 (1, 10)	3 (1, 6)
≥2, n (%)	24 (92.3)	36 (85.7)
≥3, n (%)	15 (57.7)	25 (59.5)
Prior systematic anti-tumor therapy		
Fluoropyrimidine/Oxaliplatin/Irinotecan	26 (100.0)/26 (100.0)/22 (84.6)	41 (97.6)/42 (100.0)/38 (90.5)
Anti-VEGF therapy	23 (88.5)	39 (92.9)
Regorafenib and/or trifluridine/tipiracil and/or Fruquintinib	4 (15.4)	11 (26.2)

### Efficacy

**Fig. 2:**
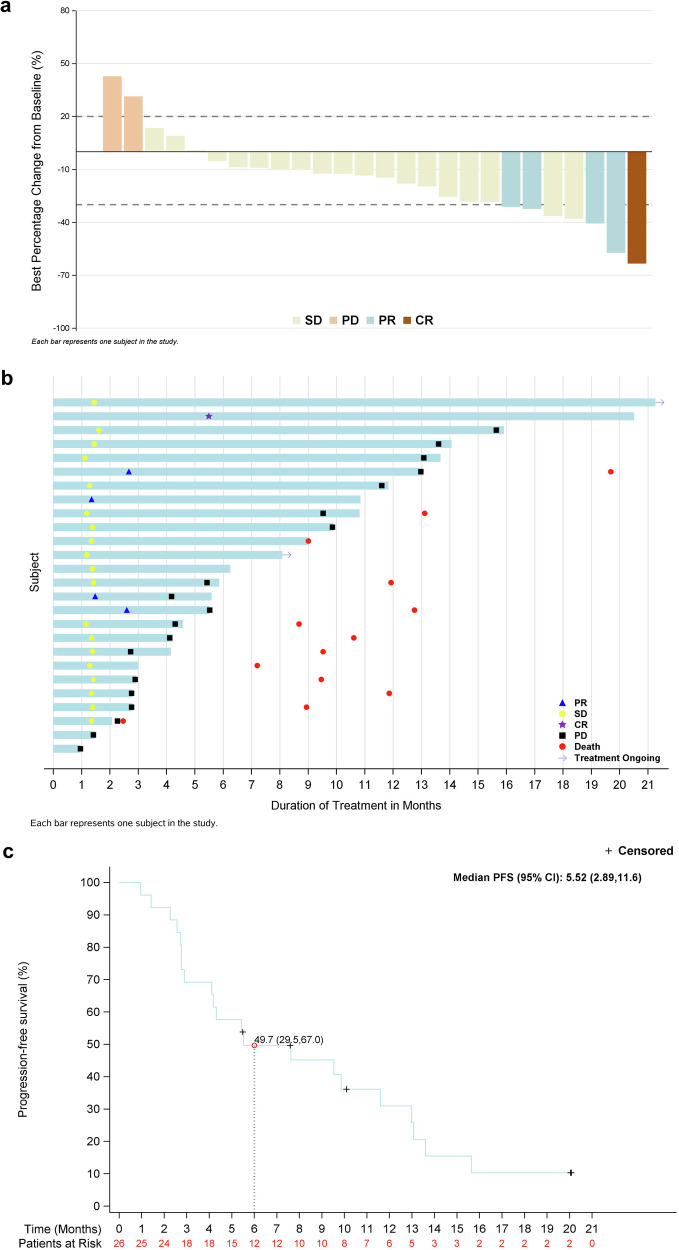
Efficacy outcomes with garsorasib monotherapy. **a** Best tumor response and tumor burden change from baseline. **b** Swimmer plot of time to response, treatment duration. **c** Kaplan-Meier estimates of progression-free survival

As of February 29, 2024, among 26 patients who were treated with single-agent garsorasib, one patient achieved complete response (CR), 4 achieved partial response (PR) and 19 had stable disease (SD) (Table 2; Fig. 2a). The confirmed ORR and disease control rate (DCR) were 19.2% (95% CI, 6.6–39.4) and 92.3% (95% CI, 74.9–99.1), respectively (Table 2). The median time to response and median duration of response were 2.6 months (range, 1.4–5.5), and 10.3 months (95% CI, 2.7–not estimated [NE]), respectively (Fig. 2b; Table 2). The median PFS was 5.5 months (95% CI, 2.9–11.6) (Fig. 2c; Supplementary Table 2). The Kaplan-Meier estimates of PFS were 49.7% (95% CI, 29.5–67.0) at 6 months, 45.2% (95% CI, 25.5–63.0) at 9 months, and 31.0% (95% CI, 13.9–49.9) at 12 months (Supplementary Table 2). The median OS was 13.1 months (95% CI, 9.5-NE) (Supplementary Table 3). The Kaplan-Meier estimates of OS were 96.0% (95% CI, 74.8–99.4) at 6 months, 83.6% (95% CI, 62.0–93.5) at 9 months and 58.0% (95% CI, 35.9–74.8) at 12 months (Supplementary Table 3).

**Table 2:** Clinical anti-tumor activity summary

	Monotherapy cohort (n = 26)	Combination cohort (n = 42)
**Time to Response (Months)**		
Median	2.6	1.7
Min, Max	1.4, 5.5	1.2, 13.8
**Best Overall Response, n (%)**		
Complete response	1 (3.8)	1 (2.4)
Partial response	4 (15.4)	18 (42.9)
Stable disease	19 (73.1)	20 (47.6)
Progressive disease	2 (7.1)	2 (4.8)
Unable to evaluate	0	1 (2.4)
**Disease control rate, n (%)**	24 (92.3)	39 (92.9)
95% CI	74.9, 99.1	80.5, 98.5
**Confirmed objective response rate, n (%)**	5 (19.2)	19 (45.2)
95% CI	6.6, 39.4	29.8, 61.3
**Median duration of response, months**	10.3	8.2
95% CI	2.7, NE	4.2, NE
**Median progression-free survival, months**	5.5	7.5
95% CI	2.9, 11.6	5.5, 8.1
**Median overall survival, months**	13.1	Not reached
95% CI	9.5, NE	11.3, NE

**Fig. 3:**
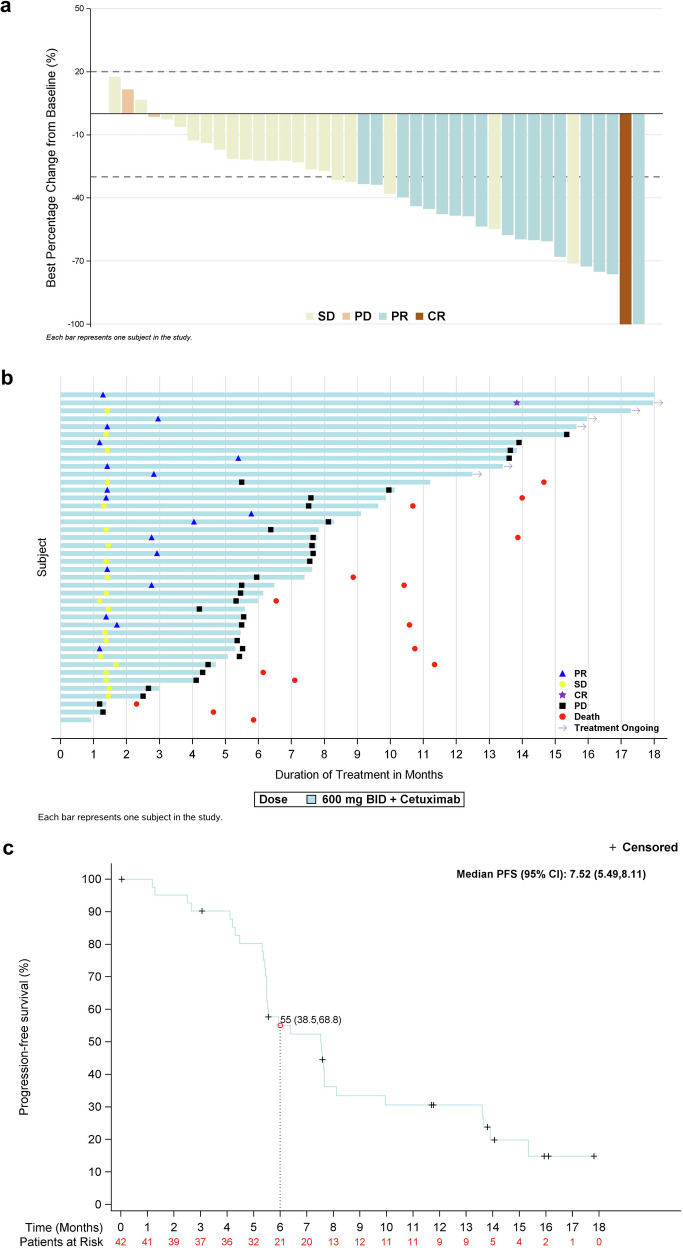
Efficacy outcomes with garsorasib plus cetuximab. **a** Best tumor response and tumor burden change from baseline. **b** Swimmer plot of time to response, treatment duration. **c** Kaplan-Meier estimates of progression-free survival

In the combination therapy cohort, of the 42 patients who received garsorasib plus cetuximab, one patient achieved CR, 18 achieved PR and 20 had SD (Table 2; Fig. 3a), with a confirmed ORR and DCR of 45.2% (95% CI, 29.8–61.3) and 92.9% (95% CI, 80.5–98.5), respectively. The median time to response and median duration of response were 1.7 months (range, 1.2–13.8), and 8.2 months (95% CI, 4.2-NE), respectively (Fig. 3b; Table 2). The median PFS was 7.5 months (95% CI, 5.5–8.1) (Fig. 3c; Supplementary Table 2). The Kaplan-Meier estimates of PFS were 55.0% (95% CI, 38.5–68.8) at 6 months, 33.4% (95% CI, 19.2–48.3) at 9 months and 30.6% (95% CI, 16.9–45.5) at 12 months (Supplementary Table 2). The median OS was not reached (95% CI, 11.3-NE) as data cut-off (Supplementary Table 3). The Kaplan-Meier estimates of OS at 6 months, 9 months, and 12 months were 92.7% (95% CI, 79.0–97.6), 82.5% (95% CI, 66.7–91.3), and 67.5% (95% CI, 49.7–80.2), respectively (Supplementary Table 3).

Exploratory subgroup analyses for ORR across key clinical subgroups both in monotherapy and combination therapy cohorts are shown in Supplementary Fig. 1 and Supplementary Fig. 2, respectively. Representative computed tomography (CT) scans showing the treatment effect of garsorasib monotherapy and garsorasib plus cetuximab are provided in Supplementary Fig. 3 and Supplementary Fig. 4, respectively.

### Safety

Treatment-related adverse events (TRAEs) of any grade were reported in 14 (53.8%) patients in the monotherapy cohort, most of which were grade 1-2 (Table 3). The most common (≥10%) TRAEs were liver function abnormalities and gastrointestinal events (Table 3). The full list of TRAEs of any grade is provided in the Supplementary Table 4. TRAEs of grade 3 or 4 occurred in 5 (19.2%) patients, including increased alanine aminotransferase, increased aspartate aminotransferase, increased gamma-glutamyl transferase, diarrhea, and hyperbilirubinaemia. TRAEs led to dose reduction or interruption in 5 (19.2%) patients, and treatment discontinuation in one (3.8%) patient (Table 3). No fatal TRAEs were reported.

**Table 3:** TRAEs summary

	Monotherapy cohort (n = 26)	
**TRAE Summary, n (%)**		
TRAEs of any grade	14 (53.8)	
Grade 3-4 TRAEs	5 (19.2)	
Leading to Dose Reduction or Interruption	5 (19.2)	
Leading to Drug Discontinuation	1 (3.8)	
Leading to Death	0	
**Most Common (≥10%) TRAEs, n (%)**	**Any Grade**	**Grade 3-4**
Alanine aminotransferase increased	8 (30.8)	3 (11.5)
Aspartate aminotransferase increased	7 (26.9)	2 (7.7)
Diarrhea	3 (11.5)	1 (3.8)
Blood bilirubin increased	3 (11.5)	0

	Combination cohort (n = 42)	
**TRAE Summary, n (%)**	**Garsorasib**	**Cetuximab**
TRAEs of any grade	42 (100)	
Grade 3-4 TRAEs	6 (14.3)	
Leading to Dose Reduction or Interruption	4 (9.5)	11 (26.2)
Leading to Drug Discontinuation	0	1 (2.4)
Leading to Death	0	0
**Most Common (≥10%) TRAEs, n (%)**	**Any Grade**	**Grade 3-4**
Rash	30 (71.4)	2 (4.8)
Aspartate aminotransferase increased	15 (35.7)	0
Paronychia	14 (33.3)	0
Alanine aminotransferase increased	13 (31.0)	0
Blood bilirubin increased	9 (21.4)	0
Dry skin	7 (16.7)	0
Proteinuria	6 (14.3)	0
Skin fissures	6 (14.3)	0
Dermatitis acneiform	5 (11.9)	1 (2.4)
Nausea	5 (11.9)	0
Pruritus	5 (11.9)	0

In the combination cohort, TRAEs of any grade were reported in 42 (100%) patients, most of which were grade 1–2 (Table 3, Supplementary Table 5). The most common (≥10%) TRAEs were skin and subcutaneous tissue disorders, and liver function abnormalities (Table 3). The full list of TRAEs of any grade is provided in the Supplementary Table 5. TRAEs of grade 3 or 4 occurred in 6 (14.3%) patients, including rash, dermatitis acneiform, increased bilirubin conjugated, hypomagnesaemia and pruritic rash. No Grade 5 TRAEs were reported. TRAEs led to garsorasib dose reduction or interruption in 4 (9.5%) patients. No patient reported discontinuation of garsorasib due to TRAEs. TRAEs led to cetuximab dose reduction or interruption in 11 (26.2%) patients, and cetuximab discontinuation in one (2.4%) patient (Table 3).

In the monotherapy cohort, TRAE of blood bilirubin increased (11.5%), bilirubin conjugated increased (7.7%) and hyperbilirubinaemia (3.8%) were commonly observed, and TRAE of blood bilirubin increased (21.4%), bilirubin conjugated increased (9.5%) and blood bilirubin unconjugated increased (2.4%) were also commonly observed in the combination treatment cohort. Most of these adverse events were grade 1-2 with only one grade 3 hyperbilirubinaemia and one grade 3 bilirubin conjugated increased in monotherapy cohort and combination cohort, respectively. These events were resolved with garsorasib dose interruption and did not lead to garsorasib dose reduction or permanent discontinuation. No Hy’s law cases were reported.

## Discussion

Approximately 2–4% of mCRC patients harbor the KRAS G12C mutation,^3,12,22,23^ FDA granted approval to adagrasib plus cetuximab and sotorasib plus panitumumab only recently and there is still a great unmet need for such patients who have a dismal prognosis. In this study, garsorasib was evaluated as a single agent and in combination with cetuximab in non-parallel and non-randomized cohorts of heavily pretreated KRAS G12C-mutated mCRC patients. The combination of garsorasib and cetuximab demonstrated higher ORR and a trend toward improved PFS and OS compared with garsorasib alone, suggesting potential synergistic effect between a KRAS inhibitor and an anti-EGFR antibody. This finding is consistent with the discoveries made in preclinical studies^13,14,24^ and the observations from clinical studies involving drugs of the same class.^15,16^

In the present study, no unmanageable safety concerns were identified in either cohort. In the monotherapy cohort, the most common TRAEs were liver function abnormalities and gastrointestinal events, which were reversible and manageable with dose interruption and supportive medication. The incidence and severity observed in the monotherapy cohort were generally consistent with those observed in a pooled analysis of the safety of garsorasib among 306 patients for the purpose of marketing approval in China (refer to garsorasib label in China for details). These events were clinically manageable and resolved with garsorasib dose interruption and/or reduction and usually did not lead to treatment discontinuation. On the other hand, TRAE of blood bilirubin increased was reported in 11.5% and 21.4% of patients in garsorasib monotherapy and combination therapy cohorts, respectively. However, most of these adverse events were grade 1–2 with only one grade 3 event in each cohort, and no Hy’s law cases were reported. There is also no trend of dose-related hepatotoxicity in the dose escalation study of garsorasib.^18^ Besides, TRAE of increased blood bilirubin was also commonly observed in some of the other KRAS G12C inhibitors such as JAB-21822 and IBI351,^25–27^ indicating that it might not be a coincidence but a common phenomenon. In terms of gastrointestinal adverse effects frequently observed in other KRAS G12C inhibitors,^11,12,28^ the incidence was generally lower in garsorasib (diarrhea at 11.5%, nausea at 7.7%, and no vomiting reported), compared to those reported of other KRAS G12C inhibitors (diarrhea at 31.7–69%, nausea at 19–78%, and vomiting at 7.9–58%). This phenomenon was also evidenced by the safety data reported in two studies evaluating garsorasib monotherapy in advanced NSCLC patients, in which the incidences of gastrointestinal adverse effects, including nausea, diarrhea and vomiting, were around 20%.^19,29^

In the combination cohort, the safety profile was generally manageable and consistent with those reported for each drug alone. There were also no observed synergistic toxic effects in the combination cohort. Dose modification and discontinuation were mainly attributed to cetuximab (26.2% and 2.4%, respectively). No patients in this cohort discontinued treatment due to garsorasib-related adverse events. Interestingly, the incidence of grade 3-4 TRAE (14.3%) in the combination cohort was numerically lower than that (19.2%) in the monotherapy cohort. Similar results were observed in the KRYSTAL-1 study evaluating adagrasib as monotherapy or in combination with cetuximab in the same populaton.^21^ TRAEs of grade 3–4 occurred in 34.1% (15/44) of patients who received adagrasib monotherapy, whereas they declined to 15.6% (5/32) in patients who received adagrasib plus cetuximab. Further studies are warranted to uncover the underlying mechanisms.

Both in the monotherapy and combination cohorts, the majority of patients (92.3% and 85.2%) had received at least two prior lines of systematic therapy, and 84.6% and 92.9% of them were pretreated with anti-VEGF therapy, respectively. Nevertheless, single-agent garsorasib and the combination of garsorasib and cetuximab achieved considerable ORRs of 19.2% and 45.2%, and a trend toward prolonged PFS and OS, as compared with the standard-of-care includes regorafenib and trifluridine/tipiracil, which demonstrated an ORR of 1.0% and 1.6%, respectively.^30,31^ Similar results were observed in other studies investigating KRAS G12C inhibitors, such as adagrasib,^16^ sotorasib,^11^ and divarasib,^32^ as monotherapy and combination therapy. Therefore, garsorasib is worthy of further investigation in phase III trial against standard-of-care therapy, especially in combination with cetuximab.

Several limitations of this study should be acknowledged. Firstly, the nonrandomized design does not allow for direct comparisons between garsorasib monotherapy/combination and standard-of-care, but garsorasib with or without cetuximab indeed demonstrated promising anti-tumor activity as later-line therapy for these mCRC subsets compared with historical control. Secondly, the sample size was relatively small in this study, and randomized clinical trials with large sample size are needed to confirm the findings. Thirdly, the present follow-up time was not enough to capture the long-term survival benefit of the combination cohort. Since promising anti-tumor activity was indicated by considerably enhanced ORR and PFS in the present study, further investigation of garsorasib plus cetuximab is needed to confirm its survival benefit in large sample study with long term follow-up.

In conclusion, favorable safety profile and promising anti-tumor activity were observed in both the garsorasib monotherapy and combination cohorts. Most importantly, this is the first study reporting the highest proportion of Asian patients with mCRC receiving a KRAS G12C inhibitor as to date, which provides further evidence supporting the generalizability of KRAS G12C inhibitors in such population. Confirmatory randomized phase III studies with large sample size are planned to further evaluate the efficacy and safety of garsorasib in combination with cetuximab versus standard-of-care as the later-line treatment in this population.

## Materials and methods

### Study design

We conducted a multicenter, phase I/II, open-label study to evaluate the safety and efficacy of single-agent garsorasib and in combination with other anti-tumor therapies in patients with advanced or metastatic KRAS G12C-mutated solid tumors, including CRC (refer to protocol for detailed study design). Phase I study comprised of two parts: monotherapy dose escalation (phase Ia from 150 mg to 1600 mg) and dose combination (phase Ib). The RP2D was determined to be 600 mg twice daily (BID) based on the results from phase Ia study,^18^ and was used in phase II stage of this study. The phase II portion is a six-arm, non-parallel, open-label, non-randomized study to evaluate the efficacy of garsorasib as single agent and in combination in patients with advanced or metastatic solid tumors with KRAS G12C mutation. Among them, Arm B enrolled patients with KRAS G12C mutated advanced solid tumors (including CRC) other than NSCLC and were treated with garsorasib at RP2D; Arm F only enrolled patients with CRC and were treated with garsorasib plus cetuximab. Here, we report the efficacy and safety results from patients with KRAS G12C-mutated advanced or metastatic CRC receiving garsorasib with or without cetuximab, which consist of a subgroup of CRC patients from Arm B and the whole Arm F in the phase II study. A total of 24 study centers from China, the United States, Australia, and South Korea contributed to the CRC patient enrollment (Supplementary Table 6). In the garsorasib monotherapy cohort, all 26 patients were from Arm B. In the garsorasib combination cohort, all 42 patients were from Arm F.

The protocol amendments related to the two cohorts in this study are as follows: 1) To include garsorasib in combination with cetuximab in dose expansion (phase II), and to refine the study procedures, such as ECG test, PK sample time points (version 1.3 to version 2.0); 2) Based on accumulated preliminary efficacy data in mCRC, anti-tumor activities were expected in phase II Arm B and Arm F, thus the previous assumptions of Simon 2 stage design in Arm B and Arm F were no longer applicable. The design of these two arms was converted into a simple expansion cohort design and the sample sizes of these arms were adjusted accordingly (version 2.0 to version 3.0).

This study adhered to Good Clinical Practice guidelines (as defined by the International Council on Harmonisation) and principles of the Declaration of Helsinki. The study protocol was approved by the independent ethics committee or institutional review board at each participating site. Written informed consent was obtained from all patients before screening. This study was registered with ClinicalTrials.gov number NCT04585035.

### Participants

Patients (≥18 years of age) with histologically or cytologically confirmed advanced or metastatic CRC harboring KRAS G12C mutation (confirmed by historical local lab results within 5 years prior to treatment, as well as by central lab for phase II), were eligible for this study. Patients receiving garsorasib alone must be refractory to or intolerant of existing standard treatment, and patients receiving garsorasib plus cetuximab should have progressed after at least one available standard therapy. Patients who had unstable or progressive central nervous system metastases, prior treatment of KRAS G12C inhibitors, or anti-EGFR therapy (only for patients receiving garsorasib plus cetuximab) were excluded.

### Procedures

Patients with advanced or metastatic CRC in the monotherapy cohort received oral garsorasib at RP2D (600 mg BID) in a fasting state. In the combination cohort, patients were treated with oral garsorasib 600 mg BID in combination with intravenous cetuximab at an initial dose of 400 mg per square meter of body-surface area (mg/m^2^) on day 1 of cycle 1 and 250 mg/m^2^ weekly thereafter. Each 21-day period was considered as one treatment cycle. Treatment continued until disease progression, withdrawal of consent, unacceptable toxicity or discontinuation from the study for other reasons.

Adverse events (AEs) were graded according to the National Cancer Institute Common Terminology Criteria for Adverse Events (NCI CTCAE) version 5.0. AEs were collected from the time the patient provided written informed consent until the end of safety follow-up (30 calendar days after the last dose of study treatment or until start of new anti-tumor therapy).

### Outcomes

The primary endpoint was ORR. Secondary endpoints included DCR, PFS, DOR, and OS, as well as type, incidence, severity, and attribution of AEs. The ORR, DCR, PFS and DOR were evaluated by the investigator according to Response Evaluation Criteria in Solid Tumors (RECIST) version 1.1.

### Sample size considerations

Although there was no formal statistical hypothesis prespecified, sample size estimation was performed to determine the sample size when we conducted this trial. The consideration of sample size was provided as follows:

Monotherapy cohort: the ORR of third-line standard-of care (SOC) treatment in mCRC is less than 5%. Assuming the ORR of garsorasib monotherapy is around 20%, 26 subjects will provide more than 80% power to assure that the ORR of garsorasib monotherapy is greater than 5%.

Combination cohort: Assuming the ORR of garsorasib in combination with cetuximab is around 40%, 42 subjects will provide more than 80% power to assure that the ORR of garsorasib in combination with cetuximab is greater than 20%.

### Statistical analysis

The efficacy analyses in this study were performed following ITT principle. All the subjects received at least one dose of investigational drug, no matter with or without post baseline tumor assessments, were included in the analysis set for efficacy analysis and safety analysis as the whole population. The protocol defined this population as the safety analysis set but it is indeed the same as the ITT population. Statistical Analysis System version 9.4 (SAS Institute, Cary, NC) was used to analyze the efficacy outcomes. ORR, DCR and their 95% confidence intervals (CI) were calculated using Clopper-Pearson method. PFS, OS and DOR were analyzed by the Kaplan-Meier method.

## Supplementary information

Supplementary Materials

Study Protocol

## Data Availability

The datasets (including de-identified individual data) generated during the current study are available from the corresponding author upon request by contacting xurh@sysucc.org.cn, not for commercial use. All requests will be reviewed by the corresponding author and the sponsor, InventisBio within 2 weeks. A signed data access agreement with the sponsor is required before data sharing.
