# Supplementary material for: Garsorasib, a KRAS G12C inhibitor, with or without cetuximab, an EGFR antibody, in colorectal cancer cohorts of a phase II trial in advanced solid tumors with KRAS G12C mutation
Source: Signal Transduct Target Ther. 2025 Jun 17;10:189. doi: 10.1038/s41392-025-02274-z (PMC12170901; doi:10.1038/s41392-025-02274-z)
Supplement: Supplementary file 1 — Supplementary Materials [file 41392_2025_2274_MOESM1_ESM.docx]

Supplementary Materials for

Garsorasib, a KRAS G12C inhibitor, with or without cetuximab, an EGFR antibody, in colorectal cancer cohorts of a phase II trial in advanced solid tumors with KRAS G12C mutation

Dan-Yun Ruan^#^, Hao-Xiang Wu^#^, Ye Xu, Pamela N Munster, Yanhong Deng, Gary Richardson, Dong Yan, Myung-Ah Lee, Keun-Wook Lee, Hongming Pan, Steven Hager, Xingya Li, Shaozhong Wei, Xinfang Hou, Craig Underhill, Michael Millward, Ina Nordman, Jingdong Zhang, Jianzhen Shan, Guohong Han, Jaspreet Grewal, Shirish M. Gadgeel, Rachel E. Sanborn, Seok Jae Huh, Xiaohua Hu, Yihong Zhang, Ziyong Xiang, Laisheng Luo, Xiaoxi Xie, Zhe Shi, Yaolin Wang, Ling Zhang, Feng Wang^*^, Rui-Hua Xu^*^

^#^ These authors contributed equally to the manuscript and thus share the first authorship.

^*^ Correspondence to: xurh@sysucc.org.cn; wangfeng@sysucc.org.cn.

**This PDF includes:**

Figures S1 to S4

Tables S1 to S6


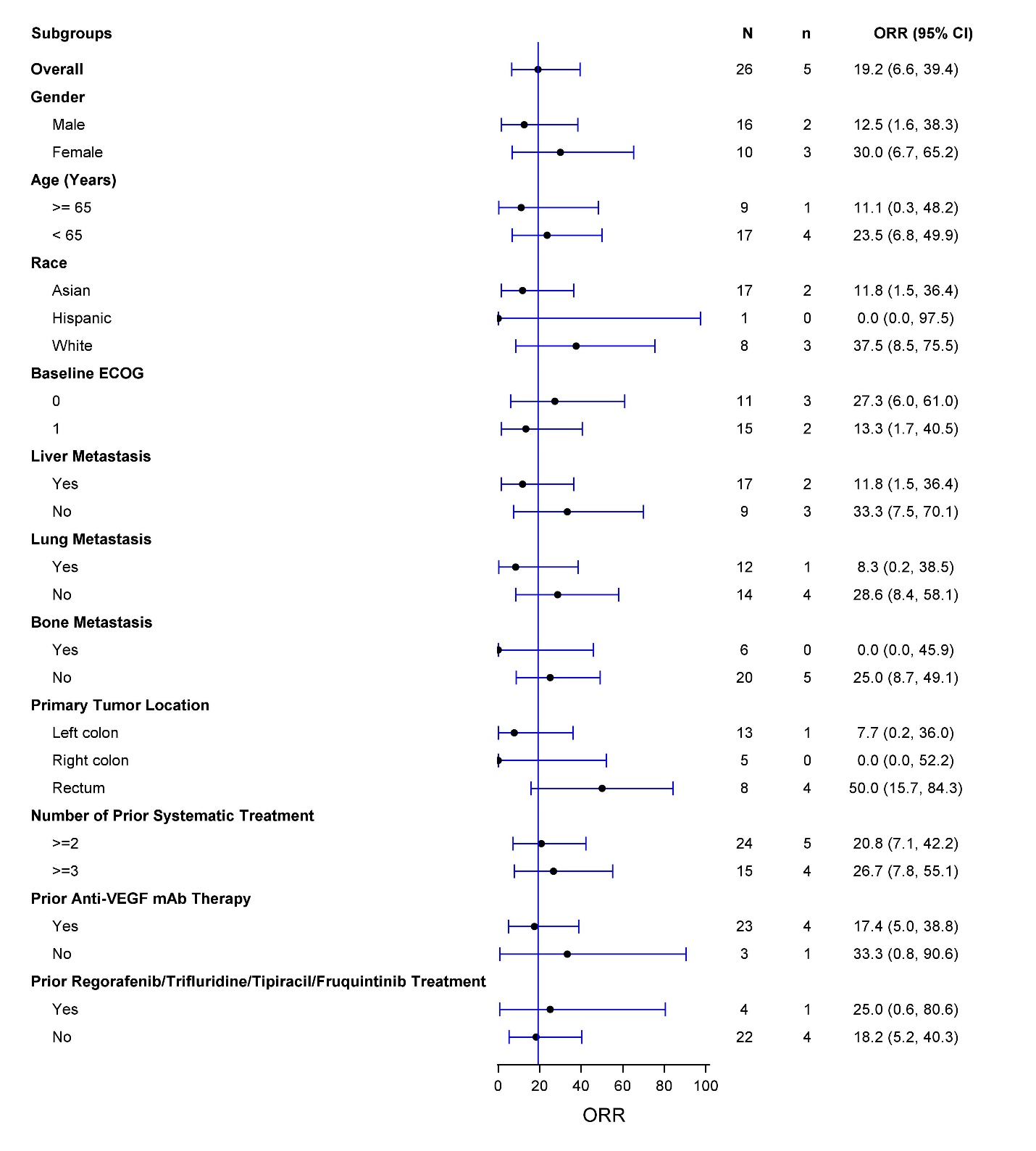


Figure S1. Subgroup Analysis for Objective Response Rate – Garsorasib Monotherapy Cohort.


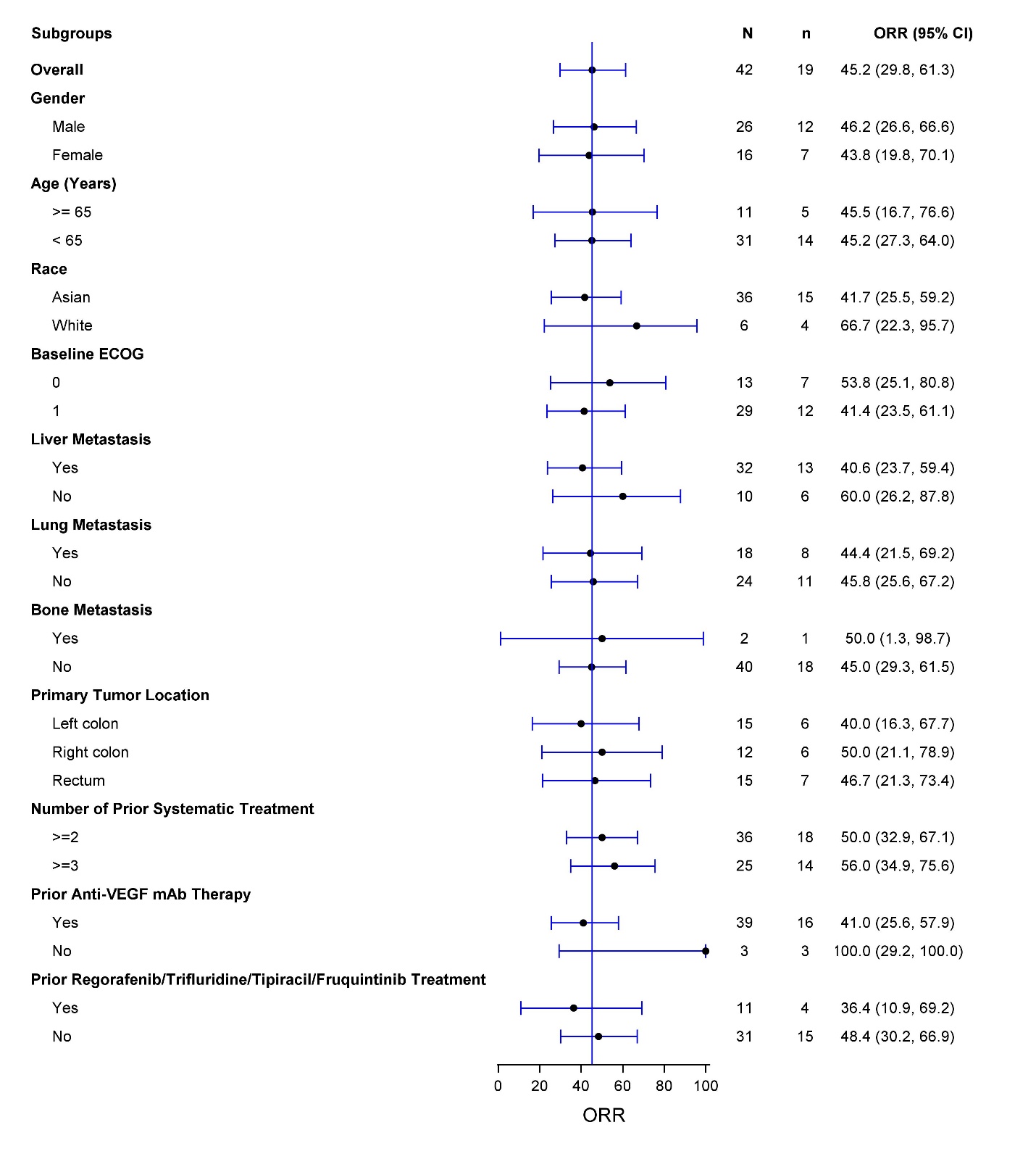


**Figure S2. Subgroup Analysis for Objective Response Rate – Garsorasib Combination Cohort.**


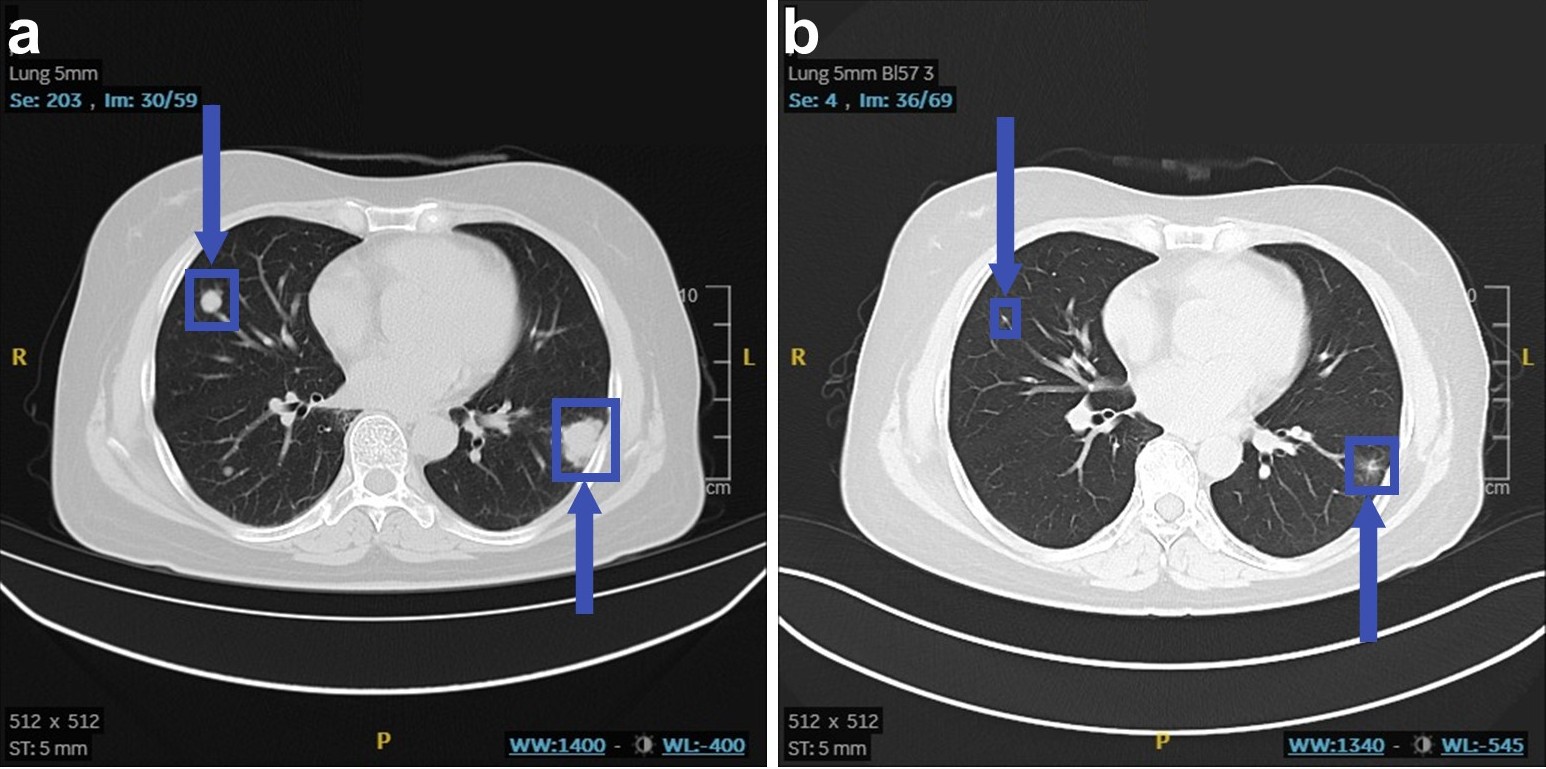


**Figure S3. Computed tomography (CT) scans showing the treatment effect of garsorasib monotherapy.** (a) CT scan before garsorasib monotherapy. (b) CT scan after garsorasib monotherapy for 61 days. Arrows indicate the target lesions.


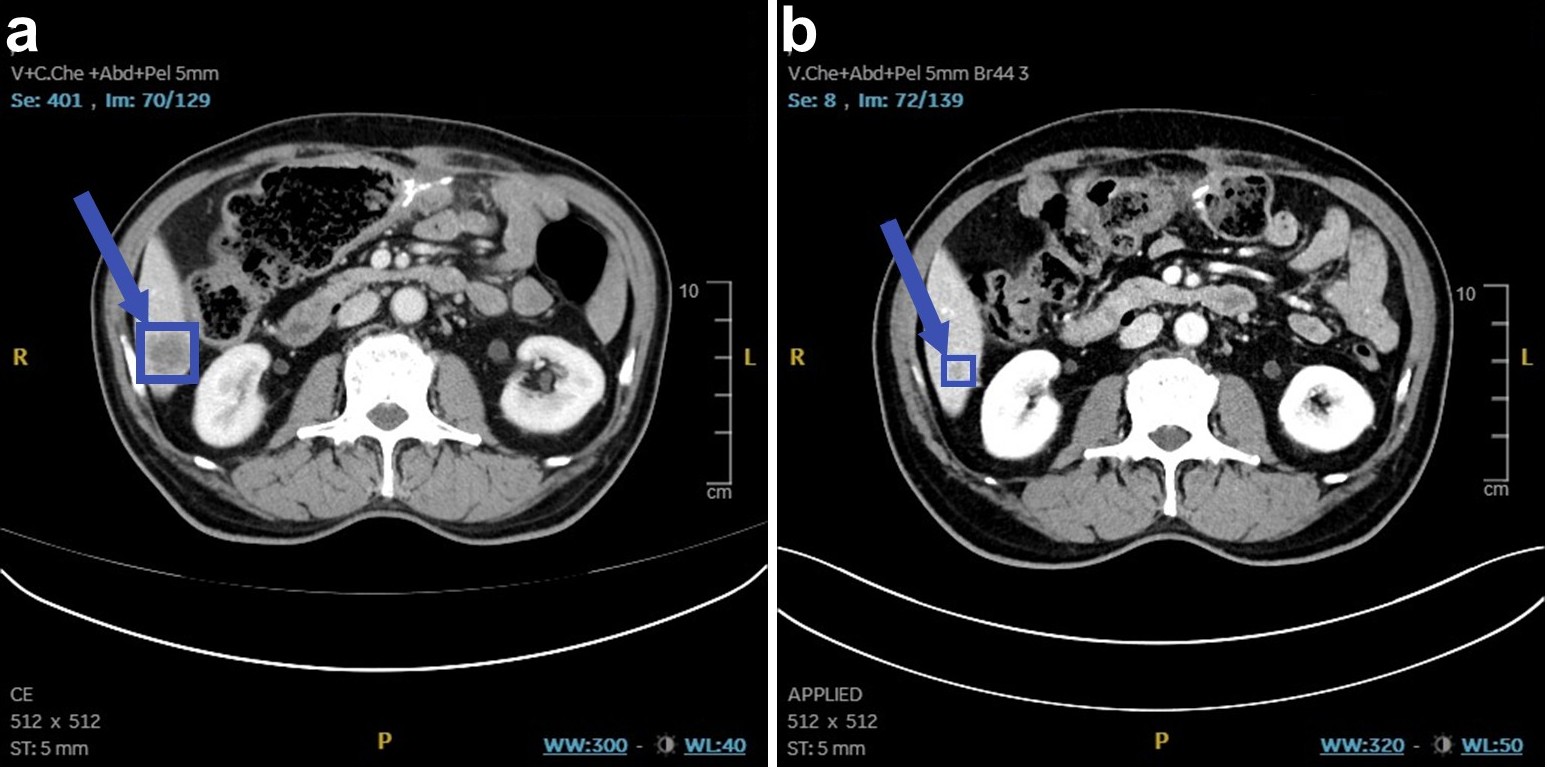


**Figure S4. Computed tomography (CT) scans showing the treatment effect of** **garsorasib plus cetuximab combination treatment.** (a) CT scan before garsorasib plus cetuximab combination treatment. (b) CT scan after garsorasib plus cetuximab combination treatment for 83 days. Arrows indicate the target lesions.

Table S1. Drug Exposure

|  | **Monotherapy cohort**  **(N=26)** | **Combination cohort**  **(N=42)** |
| --- | --- | --- |
| **Duration of Treatment (Months)** |  |  |
| n | 26 | 42 |
| Median | 6.0 | 7.7 |
| Min, Max | (0.7, 21.3) | (0.8, 18.0) |
| **Duration of Follow-up (Months)** |  |  |
| n | 26 | 42 |
| Median | 13.0 | 13.0 |
| Min, Max | (1.8, 23.3) | (2.3, 19.1) |

Table S2. Progression-free survival (Kaplan-Meier method)

| **Progression-free survival** | **Monotherapy cohort**  **(N=26)** | **Combination cohort**  **(N=42)** |
| --- | --- | --- |
| No. of Events | 21 (80.8) | 31 (73.8) |
| No. of Censored | 5 (19.2) | 11 (26.2) |
| Median & 95% CI | 5.5 (2.9, 11.6) | 7.5 (5.5, 8.1) |
| At 3 Months (%) & 95% CI | 69.2 (47.8, 83.3) | 90.2 (76.1, 96.2) |
| At 6 Months (%) & 95% CI | 49.7 (29.5, 67.0) | 55.0 (38.5, 68.8) |
| At 9 Months (%) & 95% CI | 45.2 (25.5, 63.0) | 33.4 (19.2, 48.3) |
| At 12 Months (%) & 95% CI | 31.0 (13.9, 49.9) | 30.6 (16.9, 45.5) |

Table S3. Overall survival (Kaplan-Meier method)

| **Overall survival** | **Monotherapy cohort**  **(N=26)** | **Combination cohort**  **(N=42)** |
| --- | --- | --- |
| No. of Events | 13 (50.0) | 15 (35.7) |
| No. of Censored | 13 (50.0) | 27 (64.3) |
| Median & 95% CI | 13.1 (9.5, NE) | Not reached (11.3, NE) |
| At 3 Months (%) & 95% CI | 96.0 (74.8, 99.4) | 97.6 (84.3, 99.7) |
| At 6 Months (%) & 95% CI | 96.0 (74.8, 99.4) | 92.7 (79.0, 97.6) |
| At 9 Months (%) & 95% CI | 83.6 (62.0, 93.5) | 82.5 (66.7, 91.3) |
| At 12 Months (%) & 95% CI | 58.0 (35.9, 74.8) | 67.5 (49.7, 80.2) |

Table S4. Treatment-related adverse events in monotherapy cohort

|  | **Total, n (%)** | **Grade 3/4, n (%)** |
| --- | --- | --- |
| Number of patients with at least one TRAE | 14 (53.8) | 5 (19.2) |
| Alanine aminotransferase increased | 8 (30.8) | 3 (11.5) |
| Aspartate aminotransferase increased | 7 (26.9) | 2 (7.7) |
| Diarrhoea | 3 (11.5) | 1 (3.8) |
| Blood bilirubin increased | 3 (11.5) | 0 |
| Nausea | 2 (7.7) | 0 |
| Gamma-glutamyltransferase increased | 2 (7.7) | 2 (7.7) |
| Bilirubin conjugated increased | 2 (7.7) | 0 |
| Hypothyroidism | 2 (7.7) | 0 |
| Blood alkaline phosphatase increased | 1 (3.8) | 0 |
| Fatigue | 1 (3.8) | 0 |
| Myalgia | 1 (3.8) | 0 |
| Rash | 1 (3.8) | 0 |
| White blood cell count decreased | 1 (3.8) | 0 |
| Dizziness | 1 (3.8) | 0 |
| Eyelid oedema | 1 (3.8) | 0 |
| Hyperbilirubinaemia | 1 (3.8) | 1 (3.8) |
| Lymphocyte count decreased | 1 (3.8) | 0 |
| Pain in extremity | 1 (3.8) | 0 |
| Platelet count decreased | 1 (3.8) | 0 |
| Pneumonitis | 1 (3.8) | 0 |
| Taste disorder | 1 (3.8) | 0 |

Table S5. Treatment-related adverse events in combination treatment cohort

|  | **Total, n (%)** | **Grade 3/4, n (%)** |
| --- | --- | --- |
| Number of patients with at least one TRAE | 42 (100) | 6 (14.3) |
| Rash | 30 (71.4) | 2 (4.8) |
| Aspartate aminotransferase increased | 15 (35.7) | 0 |
| Paronychia | 14 (33.3) | 0 |
| Alanine aminotransferase increased | 13 (31.0) | 0 |
| Blood bilirubin increased | 9 (21.4) | 0 |
| Dry skin | 7 (16.7) | 0 |
| Proteinuria | 6 (14.3) | 0 |
| Skin fissures | 6 (14.3) | 0 |
| Dermatitis acneiform | 5 (11.9) | 1 (2.4) |
| Nausea | 5 (11.9) | 0 |
| Pruritus | 5 (11.9) | 0 |
| Bilirubin conjugated increased | 4 (9.5) | 1 (2.4) |
| Constipation | 4 (9.5) | 0 |
| Diarrhoea | 4 (9.5) | 0 |
| Hypoalbuminaemia | 4 (9.5) | 0 |
| Acne | 3 (7.1) | 0 |
| Asthenia | 3 (7.1) | 0 |
| Bilirubin urine present | 3 (7.1) | 0 |
| Dizziness | 3 (7.1) | 0 |
| Dry eye | 3 (7.1) | 0 |
| Electrocardiogram QT prolonged | 3 (7.1) | 0 |
| Gastrooesophageal reflux disease | 3 (7.1) | 0 |
| Mouth ulceration | 3 (7.1) | 0 |
| Stomatitis | 3 (7.1) | 0 |
| Vomiting | 3 (7.1) | 0 |
| Anaemia | 2 (4.8) | 0 |
| Conjunctivitis | 2 (4.8) | 0 |
| Folliculitis | 2 (4.8) | 0 |
| Hypertrichosis | 2 (4.8) | 0 |
| Hypomagnesaemia | 2 (4.8) | 1 (2.4) |
| Infusion related reaction | 2 (4.8) | 0 |
| Platelet count decreased | 2 (4.8) | 0 |
| Protein urine present | 2 (4.8) | 0 |
| Skin infection | 2 (4.8) | 0 |
| Urinary tract infection | 2 (4.8) | 0 |
| Abdominal discomfort | 1 (2.4) | 0 |
| Abdominal pain upper | 1 (2.4) | 0 |
| Aphthous ulcer | 1 (2.4) | 0 |
| Arteriosclerosis | 1 (2.4) | 0 |
| Arthralgia | 1 (2.4) | 0 |
| Atrioventricular block first degree | 1 (2.4) | 0 |
| Blepharitis | 1 (2.4) | 0 |
| Blood bilirubin unconjugated increased | 1 (2.4) | 0 |
| Blood lactate dehydrogenase increased | 1 (2.4) | 0 |
| Blood thyroid stimulating hormone increased | 1 (2.4) | 0 |
| Chills | 1 (2.4) | 0 |
| Decreased appetite | 1 (2.4) | 0 |
| Dermatitis | 1 (2.4) | 0 |
| Dermatitis allergic | 1 (2.4) | 0 |
| Dysuria | 1 (2.4) | 0 |
| Eczema | 1 (2.4) | 0 |
| Eczema infected | 1 (2.4) | 0 |
| Erythema | 1 (2.4) | 0 |
| Face oedema | 1 (2.4) | 0 |
| Fatigue | 1 (2.4) | 0 |
| Flushing | 1 (2.4) | 0 |
| Gamma-glutamyltransferase increased | 1 (2.4) | 0 |
| Gingival bleeding | 1 (2.4) | 0 |
| Headache | 1 (2.4) | 0 |
| Hypersensitivity | 1 (2.4) | 0 |
| Hyperthyroidism | 1 (2.4) | 0 |
| Hypocalcaemia | 1 (2.4) | 0 |
| Hypokalaemia | 1 (2.4) | 0 |
| Hyponatraemia | 1 (2.4) | 0 |
| Insomnia | 1 (2.4) | 0 |
| Keratitis | 1 (2.4) | 0 |
| Lipase increased | 1 (2.4) | 0 |
| Malaise | 1 (2.4) | 0 |
| Meibomian gland dysfunction | 1 (2.4) | 0 |
| Micturition urgency | 1 (2.4) | 0 |
| Muscle spasms | 1 (2.4) | 0 |
| Neurodermatitis | 1 (2.4) | 0 |
| Neuropathy peripheral | 1 (2.4) | 0 |
| Oedema peripheral | 1 (2.4) | 0 |
| Onychoclasis | 1 (2.4) | 0 |
| Pain | 1 (2.4) | 0 |
| Palmar-plantar erythrodysaesthesia syndrome | 1 (2.4) | 0 |
| Pneumonia | 1 (2.4) | 0 |
| Pollakiuria | 1 (2.4) | 0 |
| Pyrexia | 1 (2.4) | 0 |
| Rash maculo-papular | 1 (2.4) | 0 |
| Rash papular | 1 (2.4) | 0 |
| Rash pruritic | 1 (2.4) | 1 (2.4) |
| Rash pustular | 1 (2.4) | 0 |
| Red blood cells urine positive | 1 (2.4) | 0 |
| Sinus tachycardia | 1 (2.4) | 0 |
| Skin toxicity | 1 (2.4) | 0 |
| Supraventricular extrasystoles | 1 (2.4) | 0 |
| Trichorrhexis | 1 (2.4) | 0 |
| Urine bilirubin increased | 1 (2.4) | 0 |
| Urobilinogen urine increased | 1 (2.4) | 0 |
| Vision blurred | 1 (2.4) | 0 |
| White blood cells urine positive | 1 (2.4) | 0 |

**Table S6. Number of patients enrolled in each study site**

| **Monotherapy cohort (N=26)** |  |  |
| --- | --- | --- |
| **Site** | **Country/Area** | **Patients enrolled, n** |
| Sun Yat-sen University Cancer Center | China | 5 |
| Cabrini Haematology and Oncology Centre | Australia | 3 |
| The Catholic University of Korea, Seoul St. Mary's Hospital | Korea, Republic of | 3 |
| The Sixth Affiliated Hospital of Sun Yat-Sen University | China | 3 |
| Seoul National University Bundang Hospital | Korea, Republic of | 2 |
| UCSF Medical Center - Pulmonary Hypertension Clinic | United States | 2 |
| Asan Medical Center | Korea, Republic of | 1 |
| California Cancer Associates for Research and Excellence (CCARE) - Fresno | United States | 1 |
| Dong-A University Hospital | Korea, Republic of | 1 |
| Henan Cancer Hospital | China | 1 |
| Henry Ford Health System | United States | 1 |
| Hubei Cancer Hospital | China | 1 |
| Linear Clinical Research | Australia | 1 |
| Providence Portland | United States | 1 |
| **Combination cohort (N=42)** |  |  |
| **Site** | **Country/Area** | **Patients enrolled, n** |
| Sun Yat-sen University Cancer Center | China | 14 |
| Fudan University Shanghai Cancer Center | China | 5 |
| Beijing Luhe Hospital, Capital Medical University | China | 3 |
| UCSF Medical Center - Pulmonary Hypertension Clinic | United States | 3 |
| Sir Run Run Shaw Hospital (SRRSH), affiliated with the Zhejiang University School of Medicine | China | 2 |
| The First Affiliated Hospital of Zhengzhou University | China | 2 |
| The Sixth Affiliated Hospital of Sun Yat-Sen University | China | 2 |
| Hubei Cancer Hospital | China | 2 |
| Border Medical Oncology | Australia | 1 |
| Cabrini Haematology and Oncology Centre | Australia | 1 |
| Calvary Mater Newcastle | Australia | 1 |
| Henan Cancer Hospital | China | 1 |
| Liaoning Cancer Hospital & Institute | China | 1 |
| Seoul National University Bundang Hospital | Korea, Republic of | 1 |
| The First Affiliated Hospital of Zhejiang University School of Medicine | China | 1 |
| The First Affiliated Hospital of Guangxi Medical University | China | 1 |
| Xi 'an International Medical Center Hospital | China | 1 |
