# Supplementary material for: Garsorasib, a KRAS G12C inhibitor, with or without cetuximab, an EGFR antibody, in colorectal cancer cohorts of a phase II trial in advanced solid tumors with KRAS G12C mutation
Source: Signal Transduct Target Ther. 2025 Jun 17;10:189. doi: 10.1038/s41392-025-02274-z (PMC12170901; doi:10.1038/s41392-025-02274-z)
Supplement: Supplementary file 2 — Study Protocol [file 41392_2025_2274_MOESM2_ESM.pdf]

CLINICAL PROTOCOL

A Phase 1/2, Open-Label Study to Evaluate the Safety, Tolerability, Pharmacokinetics and Efficacy of D-1553 in Subjects with Advanced or Metastatic Solid Tumors with KRAS^{G12C} Mutation

PROTOCOL NUMBER:	D1553-101
VERSION NUMBER:	3.1
PROTOCOL DATE:	02 December 2022
PRODUCT:	D-1553
PHASE:	1/2
SPONSOR:	InventisBio Co., Ltd.<br>Room 210, Building #4, 67 Libing Road,<br>Zhangjiang Hi-Tech Park, Pudong,<br>Shanghai, 201203, P. R. of China<br><br>InventisBio US<br>25A Hanover Road, Suite 340<br>Florham Park, NJ 07932

This document contains confidential information belonging to InventisBio. Acceptance of this document constitutes the agreement by the recipient that no unpublished information contained herein will be published or disclosed without the prior written approval of InventisBio, except that this document may only be disclosed to the appropriate institutional review boards/ethical committees so long as they are requested to maintain its confidentiality.

STATEMENT OF COMPLIANCE

The study is carried out in accordance with the Good Clinical Practice (GCP) as required by the following:

- International Council for Harmonisation of Technical Requirements for Pharmaceuticals for Human Use (ICH) E6 R2 (2016);
- United States (US) Code of Federal Regulations (CFR) applicable to clinical studies (45 CFR Part 46; 21 CFR Part 50, 21 CFR Part 56, and 21 CFR Part 312);
- Local regulations in countries where the trial is conducted.

SIGNATURE PAGE

The signature below constitutes the approval of this protocol and its attachments and provides the necessary assurances that this trial is conducted according to all stipulations of the protocol, including all statements regarding confidentiality and according to the local legal and regulatory requirements and the applicable US federal regulations and ICH guidelines.

Sponsor: InventisBio Co., Ltd.

Signed: ________________________________ Date: ________________

Name: Ling Zhang, MD, PhD

Title: Chief Medical Officer

SIGNATURE PAGE

The signature below constitutes the approval of this protocol and its attachments and provides the necessary assurances that this trial is conducted according to all stipulations of the protocol, including all statements regarding confidentiality and according to the local legal and regulatory requirements and the applicable US federal regulations and ICH guidelines.

Site Investigator: *

Signed: ______________________________ Date: ______________

Name:

Title: Principal Investigator

** The protocol should be signed by the local investigator who is responsible of the study implementation at his/her specific site; i.e., for Investigational New Drug study of US, the individual who signs the Form FDA 1572.*

Table of Contents

CLINICAL PROTOCOL 1

STATEMENT OF COMPLIANCE 2

SIGNATURE PAGE 3

SIGNATURE PAGE 4

TABLE OF CONTENTS 5

LIST OF TABLES 10

LIST OF FIGURES 11

LIST OF ABBREVIATIONS AND DEFINITION OF TERMS 12

RATIONALE AND SUMMARY OF CHANGES FROM PROTOCOL VERSION 1.3 15

RATIONALE AND SUMMARY OF CHANGES FROM PROTOCOL VERSION 2.0 16

RATIONALE AND SUMMARY OF CHANGES FOR PROTOCOL VERSION 3.0 17

RATIONALE AND SUMMARY OF CHANGES FOR PROTOCOL VERSION 3.1 18

1 PROTOCOL SUMMARY 19

1.1 Synopsis 19

1.2 Schema 33

1.3 Schedule of Visits and Procedures 35

2 INTRODUCTION 57

2.1 Background of D-1553 57

2.2 [redacted] 57

2.3 Background of Cetuximab 59

2.4 Pharmacology 60

2.5 Pharmacokinetics and ADME 60

2.6 Toxicology 61

2.7 Study Rationale 62

2.8 Rationale for Selection of the Starting Dose of D-1553 62

2.9 Dose Escalation 63

2.10 [redacted] 63

2.11 [redacted] 64

2.12 [redacted] 65

2.13 Benefit/Risk Assessment 66

3 STUDY OBJECTIVES AND ENDPOINTS 68

3.1 Phase 1 Objective(s) 68

3.1.1 Primary Objectives 68

3.1.2 Secondary Objectives 68

3.2 Phase 1 Endpoints 68

3.2.1 Primary Endpoints 68

3.2.2 Secondary Endpoints 68

3.3 Phase 2 Objective(s) 68

3.3.1 Primary Objective: 68

3.3.2 Secondary Objectives: 69

3.4 Phase 2 Endpoints 69

3.4.1 Primary Endpoint 69

3.4.2 Secondary Endpoints 69

4 INVESTIGATIONAL PLAN 70

4.1 Overall Study Design 70

4.1.1 Phase 1a (Dose Escalation) 70

4.1.2 Phase 1b (Dose Combination) 72

4.1.3 Phase 2 75

4.1.4 Dose Escalation and Stopping Rules 77

4.1.5 Definition of Maximum Tolerated Dose/Recommended Phase 2 Dose 78

4.2 Dose-limiting Toxicity Criteria 78

4.2.1 [redacted] 78

4.2.2 [redacted] 80

4.3 Study Termination Criteria 80

5 STUDY POPULATION 81

5.1 Inclusion Criteria 81

5.2 Exclusion Criteria 84

5.3 Subject Completion and Withdrawal 87

5.3.1 Subject Completion 87

5.3.2 Subject Withdrawal 88

5.4 Subject Replacement 88

6 STUDY TREATMENT 89

6.1 Allocation to Treatment 89

6.2 Investigational Product D-1553 89

6.2.1 Formulation, Appearance, Packaging and Labeling 89

6.2.2 Handling and Storage 89

6.2.3 Product Accountability 90

6.2.4 Administration 90

6.2.5 Discontinuation of Study Treatment 91

6.2.6 Dose Modification 91

6.2.7 Dose Interruptions/Delay/Reductions 92

[redacted]

6.5 Cetuximab 99

6.5.1 Administration 99

6.5.2 Dose Modification 100

6.5.3 Adverse Reaction 101

6.6 Other Medications in combination groups/arms 101

6.7 Concomitant Medications/Treatments 101

6.7.1 Prohibited Concomitant Therapy (during treatment and within 28 days after drug discontinuation) 101

6.7.2 Concomitant Therapy requiring caution during the study 102

6.7.3 Permitted Concomitant Therapy during the study 103

6.8 [redacted] 105

6.9 Compliance 105

7 STUDY PROCEDURES ... 107
7.1 Phase 1 Procedures ... 107
7.1.1 Phase 1 - Screening Procedures (Day-28 To Day -1) ... 107
7.1.2 Phase 1 – Pre-dose Assessment (Day-1 To Day 1) ... 108
7.1.3 Phase 1 - Treatment (Day 1 of Treatment Cycle 1) ... 109
7.1.4 Phase 1 - Day 2 of Treatment Cycle 1 ... 109
7.1.5 Phase 1b (Day 8 of Treatment Cycle 1) ... 109
7.1.6 Phase 1 - Day 14 of Cycle 1 ... 110
7.1.7 Phase 1 - Day 1 of Treatment Cycle ≥2 ... 110
7.1.8 Phase 1 - Upon Disease Progression or Unacceptable AE or Withdrawal from Study ... 111
7.1.9 Phase 1 - Follow-Up ... 111
7.2 Phase 2 Procedures ... 112
7.2.1 Phase 2 -Molecular Pre-screening of Tumor and ctDNA Samples for KRAS Alterations 112
7.2.2 Phase 2 - Screening Procedures (Day -28 To Day -1) ... 113
7.2.3 Phase 2 – Pre-dose Assessment (Day -1 To Day 1) ... 114
7.2.4 Phase 2 -Treatment (Day 1 of Treatment Cycle 1) ... 114
7.2.5 Phase 2 –Arms [redacted] F (Day 2 of Treatment Cycle 1) ... 115
7.2.6 Phase 2 – Arms [redacted] F (Day 8 of Treatment Cycle 1) ... 115
7.2.7 Phase 2 - Day 1 of Treatment Cycle ≥2 ... 115
7.2.8 Phase 2 - Upon Disease Progression or Unacceptable AE or Withdrawal from Study ... 116
7.2.9 Phase 2 - Follow-Up ... 116
8 ADVERSE EVENT REPORTING ... 118
8.1 Adverse events ... 118
8.1.1 Definition of Adverse Events ... 118
8.1.2 Severity Assessment of Adverse Events ... 118
8.1.3 Causality Assessment of Adverse Events ... 119
8.2 Serious adverse events ... 119
8.2.1 Definition of Serious Adverse Events ... 119
8.2.2 Hospitalization ... 120
8.2.3 Disease Progression and Death ... 120
8.2.4 Other Anti-Tumor Therapy ... 121
8.2.5 SAE reporting requirements ... 121
8.3 Adverse Event of Special Interest (AESI) ... 122
8.3.1 Overdose ... 122

8.3.2 Liver function test abnormalities 122
8.4 Pregnancy 123
8.5 Collection and follow-up of AE/AESI/SAE 124
9 DATA ANALYSIS/STATISTICAL METHODS 125
9.1 Statistical Hypotheses 125
9.2 Sample Size Determination 125
9.2.1 Phase 1a (Dose Escalation) 125
9.2.2 Phase 1b (Dose Combination) 125
9.2.3 Phase 2 125
9.3 Populations for Analyses 126
9.4 Statistical Analyses 126
9.4.1 General Considerations 126
9.4.2 Primary Endpoint(s) 127
9.4.3 Secondary Endpoint(s) 127
9.4.4 Safety Analyses 127
9.4.5 Efficacy 127
9.4.6 Other Analyses 128
9.5 Interim Analyses 128
9.6 Data Monitoring Committee 128
9.7 Protocol Deviations 128
10 QUALITY CONTROL AND QUALITY ASSURANCE 129
10.1 Monitoring of the Study 129
10.2 Audit 129
10.3 Inspection 129
10.4 Study Record Retention 129
11 DATA HANDLING AND RECORD KEEPING 130
11.1 Data Collection 130
11.2 Case Report Forms and Source Documents 130
11.3 Access to Source Documents 130
11.4 Data Management 130
12 ETHICS/PROTECTION OF THE HUMAN SUBJECTS 132
12.1 Ethical Conduct of the Study 132

12.2 Subject Data Protection ... 132
12.3 Ethics and Regulatory Review ... 132
12.4 Informed Consent ... 132
12.5 Changes to the Clinical Study Protocol and Informed Consent Document ... 132
13 RISK MANAGEMENT ... 134
14 REFERENCES ... 135
15 APPENDIX ... 138
APPENDIX 1 ECOG PERFORMANCE STATUS ... 138
APPENDIX 2 PROTOCOL REQUIRED LABORATORY ASSESSMENTS ... 139
APPENDIX 3 LIST OF CONCOMITANT MEDICATIONS TO BE USED WITH CAUTION ... 140
APPENDIX 4 PROHIBITED CONCOMITANT MEDICATION ... 141

List of Tables

Table 1-1 Schedule of Activities for Phase 1a (Dose Escalation) ... 35
Table 1-2 Schedule of Activities for Phase 1b (Dose Combination) ... 39
Table 1-3 Schedule of Activities for Phase 2: [redacted] Arm B (Single Agent) ... 44
Table 1-4 Schedule of Activities for Phase 2: [redacted] Arm F (Combination) ... 49
Table 1-5 Plasma Pharmacokinetic Blood Sampling for Phase 1 ... 55
Table 1-6 Plasma Pharmacokinetic Blood Sampling and ECG for Phase 2 ... 56
Table 2-1 Human Starting Dose Based on Preclinical Studies of D-1553 ... 63
Table 4-1 Dose Escalation Cohorts For Phase 1a ... 70
Table 4-2 Intermediate Dose Escalation Cohorts for Phase 1a ... 72
Table 4-3 Simon 2-stage design futility criteria for [redacted]. ... 75
Table 6-1 Phase 1a (Dose Escalation) ... 91
Table 6-2 Dose Modification Table ... 91
Table 6-3 [redacted] ... 94
Table 6-4 [redacted] ... 98
Table 8-1 National Cancer Institute Common Terminology Criteria for Adverse Events Severity Grading ... 119

Table 8-2 Criteria for Liver Function Test Abnormalities 123
Table 9-1 Populations for Analysis 126

List of Figures

Figure 1-1 Study Design for Phase 1 33
Figure 1-2 Study Design for Phase 2 34

LIST OF ABBREVIATIONS AND DEFINITION OF TERMS

Term	Definition
AE	Adverse Event
AESI	Adverse Event of Special Interest
ALT	Alanine Aminotransferase
AST	Aspartate Aminotransferase
AUC	Area Under the Concentration-time curve
AUC_{0-t}	Area Under the Concentration-time curve from the time of dosing to time t
AUC_{inf}	Area Under the Concentration-time curve from the time of dosing extrapolated to time infinity
BUN	Blood Urea Nitrogen
CapeOx	Capecitabine plus Oxaliplatin
CFR	Code of Federal Regulations
CL/F	Clearance
C_{max}	Maximum Concentration
C_{min}	Minimum Concentration
CNS	Central Nervous System
COVID-19	Coronavirus Disease 2019
CR	Complete Response
CRC	Colorectal Cancer
CRO	Contract Research Organization
CT	Computerized Tomography
CYP	Cytochrome 450
DCR	Disease Control Rate
DLT	Dose-limiting Toxicity
DOR	Duration of Response
ECG	Electrocardiogram
ECOG	Eastern Cooperative Oncology Group
eCRF	Electronic Case Report Form

Term	Definition
FDA	Food and Drug Administration
FIH	First-in-human
GCP	Good Clinical Practice
GGT	Gamma-glutamyltransferase
GI	Gastrointestinal
GLP	Good Laboratory Practices
HIV	Human Immunodeficiency Virus
HNSTD	Highest Non-Severely Toxic Dose
IB	Investigator's Brochure
ICF	Informed Consent Form
ICH	International Council for Harmonisation of Technical Requirements for Pharmaceuticals for Human Use
IEC	Independent Ethics Committee
INR	International Normalized Ratio
IRB	Institutional Review Board
KRAS	Kirsten Rat Sarcoma oncogene homolog
LDH	Lactate Dehydrogenase
LVEF	Left Ventricular Ejection Fraction
mFOLFOX 6	Modified Leucovorin, Fluorouracil and Oxaliplatin 6
MRI	Magnetic Resonance Imaging
MRT	Mean Residence Time
MTD	Maximum Tolerated Dose
NCI CTCAE	National Cancer Institute Common Terminology Criteria for Adverse Events
NSCLC	Non-small Cell Lung Cancer
ORR	Objective Response Rate
OS	Overall Survival
OTC	Over-the-counter
PFS	Progression-free Survival

Term	Definition
PK	Pharmacokinetic(s)
PKS	Pharmacokinetic(s) analysis set
PPS	Per Protocol Analysis Set
PR	Partial Response
pRBC	Packed Red Blood Cell
aPTT	Activated Partial Thromboplastin Time
QTcF	Corrected QT Interval using Fridericia's Formula
RBC	Red Blood Cell
RECIST	Response Evaluation Criteria in Solid Tumors
RP2D	Recommended Phase 2 dose
SAE	Serious Adverse Event
SAP	Statistical Analysis Plan
SARS-CoV-2	Severe Acute Respiratory Syndrome Coronavirus 2
SAS	Safety Analysis Set
SD	Stable Disease
SoA	Schedule of Activities
SOC	Standard of Care
SOP	Standard Operating Procedures
SRC	Safety Review Committee
STD_{10}	Severely Toxic Dose in 10% animal
$t_{½}$	Terminal elimination half-life
t_{max}	Time of maximum concentration
TRAE	Treatment related Adverse Event
ULN	Upper Limit of Normal
US	United States
V_{dss}/F	apparent Volume of Distribution
WBC	White Blood Cell
WOCBP	Woman of Child Bearing Potential
β-hCG	Beta-Human Chorionic Gonadotropin

RATIONALE AND SUMMARY OF CHANGES FROM PROTOCOL VERSION 1.3

[redacted]

Minor editorial changes have been made, including correcting spelling errors in previous version.

A Summary of Changes is provided as Appendix 5.

RATIONALE AND SUMMARY OF CHANGES FROM PROTOCOL VERSION 2.0

This amendment of Protocol D1553-101 Version 2.0 (dated 20 Oct 2021) is issued mainly as follows:

- Include D-1553 in combination with cetuximab in [redacted] dose expansion (phase 2) to refine the study procedures, such as ECG test, PK sample time points, [redacted] blood sample and tumor tissue sample for predictive biomarker and exploratory biomarker testing;
- To refine the concomitant medications requirements in this study;
- To refine the protocol inclusion and exclusion criteria accordingly;
- To update the Section 8.0 Adverse Event Reporting according to Sponsor newly effective protocol template;
- To remove the Summary of Change in appendix 5, the Summary of Change will document in separate document.

RATIONALE AND SUMMARY OF CHANGES FOR PROTOCOL VERSION 3.0

This amendment of Protocol D1553-101 Version 3.0 (dated 21 July 2022) is issued mainly as follows:

- Based on accumulated preliminary efficacy data in mCRC, antitumor activities are expected in Phase 2 Arm B and Arm F, thus the previous assumptions of Simon 2 stage design in Arm B and Arm F are no longer applicable. The design of these two arms are converted into a simple expansion cohort design and the sample sizes of these arms are adjusted accordingly;
- [redacted];
- Clarifications are made in some inclusion and exclusion criteria based on queries from sites.

RATIONALE AND SUMMARY OF CHANGES FOR PROTOCOL VERSION 3.1

This amendment of Protocol D1553-101 Version 3.1 (dated 02 December 2022) is issued mainly as follows:

- [redacted]

1 PROTOCOL SUMMARY

1.1 Synopsis

**Study Title**	A Phase 1/2, Open-Label Study to Evaluate the Safety, Tolerability, Pharmacokinetics and Efficacy of D-1553 in Subjects with Advanced or Metastatic Solid Tumors with KRAS^{G12C} Mutation
**Phase**	Phase 1/2
**Study Drug**	D-1553 (50 mg, 200 mg tablets)
**Phase 1 Objectives**	**Primary Objectives:**<br>• To assess the safety and tolerability of D-1553 single agent and in combination with [redacted] in subjects with advanced or metastatic solid tumors with Kirsten Rat Sarcoma oncogene homolog (KRAS) ^{G12C} mutation<br>• To determine the Dose-Limiting Toxicity (DLT), Maximum Tolerated Dose (MTD) and Recommended Phase 2 Dose (RP2D) of D-1553 single agent and in combination with [redacted] in subjects with advanced or metastatic solid tumors with KRAS^{G12C} mutation<br>**Secondary Objectives**<br>• To evaluate the pharmacokinetic (PK) of D-1553 alone and in combination with [redacted] in subjects with advanced or metastatic solid tumors with KRAS^{G12C} mutation<br>• To preliminarily evaluate the antitumor activity of D-1553 alone and in combination with [redacted] in subjects with advanced or metastatic solid tumors with KRAS^{G12C} mutation
**Phase 2 Objectives**	**Primary Objective**<br>• To assess the antitumor effect of D-1553 single agent and in combination with [redacted] targeted therapy in subjects with advanced or metastatic solid tumors with KRAS^{G12C} mutation<br>**Secondary Objectives**<br>• To assess the safety and tolerability of D-1553 in subjects with advanced or metastatic solid tumors with KRAS^{G12C} mutation<br>• To evaluate the PK of D-1553

**Phase 1 Endpoints**	**Primary Endpoints**<br>• Type, incidence, severity (graded by National Cancer Institute Common Terminology Criteria for Adverse Events [NCI CTCAE], v5.0), attribution and timing of adverse events (AEs)<br>• Incidence of DLTs<br>**Secondary Endpoints**<br>• PK parameters (area under the concentration time curve from the time of dosing to time t (AUC_{0-t}), area under the concentration time curve from the time of dosing extrapolated to time infinity (AUC_{inf}), mean residence time (MRT), maximum concentration (C_{max}), time of maximum concentration (t_{max}), minimum concentration (C_{min}), terminal elimination half-life ($t_{½}$), apparent volume of distribution (V_{dss}/F) and clearance (CL/F)) of D-1553 [redacted]<br>• Objective Response Rate (ORR, Complete Response [CR] + Partial Response [PR]), Disease Control Rate (DCR, [CR + PR + Stable Disease [SD]), Progression -Free Survival (PFS) and Duration of Response (DOR), evaluated by Response Evaluation Criteria in Solid Tumors (RECIST), v1.1
**Phase 2 Endpoints**	**Primary Endpoint**<br>• ORR (CR + PR)<br>**Secondary Endpoints**<br>• DCR (CR + PR + SD), PFS, DOR, evaluated by RECIST, v1.1 and overall survival (OS)<br>• Type, incidence, severity (graded by NCI CTCAE, v5.0), attribution and timing of AEs<br>• Based on PK data obtained in this study as well as PK data obtained from other studies, a population PK analysis will be performed to characterize pharmacokinetic parameters (Clearance (CL), Volume of distribution (V)) and evaluate the effect of extrinsic and intrinsic factors to support proposed dosing regimen
**Study Population Eligibility Criteria**	**<u>Inclusion criteria</u>**<br>1. Subject must be fully informed about their illness and the investigational nature of the study protocol (including foreseeable risks and possible side effects) and must have signed and dated an Institutional Review Board (IRB) /Independent Ethics Committee (IEC) approved Informed Consent Form (ICF) that is in accordance with regulatory and ethics guidelines and it must be obtained before the performance of any protocol related procedures or tests.<br>2. Subject is male or female of at least 18 years of age at the time of signing informed consent.<br>3. Subject must be willing and able to comply with all scheduled visits, treatment, laboratory tests, be able to take oral medication and accept other requirements of the study.<br>4. Subject with histologically or cytologically proven, locally advanced, unresectable and/or metastatic solid tumor.

5. Subject has $KRAS^{G12C}$ mutation in tumor tissue, blood, pleural effusion or other samples containing cancer cells or DNA. [redacted]

6. Phase 2 subjects must be able to provide a sufficient amount of representative tumor specimen (archival sample collected within 5 years) and ctDNA samples for central laboratory testing of KRAS mutation status. Subject with $KRAS^{G12C}$ mutation in tumor or ctDNA samples local testing is acceptable for eligibility; all patients in Phase 2 study will have confirmation of $KRAS^{G12C}$ mutation by Sponsor-designated central laboratory [redacted].

7. Subject has tumor type requirement as follows:

   a. Phase 1a: advanced or metastatic solid tumors for which no standard treatment is available or the subject is refractory to or intolerant of existing standard treatment;

   [redacted] [redacted]

   [redacted] [redacted]

   [redacted] [redacted]

   [redacted] [redacted]

   f. Phase 2 Arm B: advanced or metastatic solid tumors other than NSCLC for which no standard treatment is available or the subject is refractory to or intolerant of existing standard treatment;

   g. [redacted] Phase 2 Arm F: advanced or metastatic CRC that have progression of disease after at least 1 prior regimens:

      - Disease relapse during treatment or within 6 months following adjuvant therapy will be considered advanced or metastatic disease;
      - Patients who have received 2 prior regimens (i.e. those entering the study in the 3rd line setting), must have received oxaliplatin unless it was contraindicated due to underlying conditions.

8. Subject has measurable disease according to RECIST, v1.1. For Phase 1 portion, subject with evaluable disease according to RECIST, v1.1 is also acceptable.

	9. Subject in Phase 1 has an Eastern Cooperative Oncology Group (ECOG) performance status of Grade ≤ 2, [redacted] Subject in Phase 2 has an ECOG performance status of Grade 0 or 1.<br>10. Subject has adequate hematologic function, defined as:<br>• Platelet count ≥ 100 × 10^9/L;<br>• Hemoglobin level ≥ 8.0 g/dL. [redacted] Participants can be on stable dose of erythropoietin (≥ approximately 3 months);<br>• Absolute neutrophil count ≥ 1.5 × 10^9/L.<br>[redacted]<br>11. Subject has adequate renal function, defined as creatinine clearance ≥ 60 mL/min, as calculated using the modified Cockroft Gault equation or other institutional standard formula, or creatinine ≤ 1.5 × upper limit of normal (ULN).<br>12. Subject has adequate liver function, defined as:<br>• Aspartate aminotransferase (AST) levels ≤ 2.5 × ULN (if liver metastases are present, ≤ 5 × ULN);<br>• Alanine aminotransferase (ALT) levels ≤ 2.5 × ULN (if liver metastases are present, ≤ 5 × ULN);<br>• Total bilirubin ≤ 1.5 × ULN (not applicable to subjects with Gilbert's syndrome).<br>• [redacted]<br>[redacted]<br>[redacted]<br>13. Subject has adequate coagulation function, defined as prothrombin time and activated partial thromboplastin time ≤ 1.5 × ULN, and International normalized ratio (INR) ≤ 1.5. For subjects on anticoagulation therapy, the INR should be < 3.0 or within the target range of anticoagulation therapy if applicable (INR should be used instead of prothrombin time for subjects on anticoagulation therapy).<br>14. [redacted]<br>15. Female of childbearing potential must agree to abstain or use effective contraception methods from the time of signing ICF and for the duration of study participation through 6

	months after the last dose of study drug. Acceptable contraceptive methods include: oral, injected or implanted hormonal methods of contraception; intrauterine device or intrauterine system; occlusive cap (diaphragm or cervical/vault caps) with spermicide together with male condom. If needed, investigators can select from the above contraception methods to meet the country or institutional standards of an effective contraception method.<br>• A female subject of childbearing potential is any woman, regardless of sexual orientation, who meets the following criteria: a) not surgically sterile with procedures like tubal ligation, hysterectomy, bilateral salpingectomy or bilateral oophorectomy; or b) not been naturally postmenopausal for at least 12 consecutive months (i.e., has had menses at any time in the preceding 12 consecutive months).<br>16. Male subjects must agree to abstain, be surgically sterilized, or agree to use an effective contraceptive method from the time of signing of ICF and for the duration of study participation through 6 months after the last dose of study drug. Effective contraceptive methods include: a) simultaneous use of condom, and for the female partner, hormonal contraceptives or intrauterine contraceptive device (used since at least 4 weeks prior to dosing); b) simultaneous use of condom, and for the female partner, diaphragm or cervical/vault caps with spermicide. If needed, investigators can select from the above contraception methods to meet the country or institutional standards of an effective contraception method.
**Study Population Eligibility Criteria**	**<u>Exclusion Criteria</u>**<br>1. Subject has prior anticancer or investigational drug treatment within the following windows:<br>a. Prior treatment with an inhibitor specific to KRAS^{G12C} mutation, such as AMG 510, MRTX849, LY3499446, JDQ443 and GDC-6036 (for subjects received previous treatment of KRAS^{G12C} inhibitors, the subjects are acceptable to participate in [redacted] Prior treatment with EGFR inhibitors (for [redacted] Phase 2 Arm F subjects only).<br>b. Any anticancer therapy (including chemotherapy, targeted therapy, immune therapy, etc.) or any other investigational drug therapy less than 14 days or 3 half-lives (whichever is shorter) prior to first dose of study intervention.<br>2. Subject with unstable or progressive central nervous system (CNS) metastases and/or carcinomatous meningitis. Subjects with history of brain metastases are allowed, if they are clinically stable. [redacted]

3. Subjects with clinically significant cardiovascular disease, including:
    - Subject with acute myocardial infarction, severe/unstable angina; or with cardiac insufficiency of New York Heart Association Functional Classification Grade 2 or above;
    - Subject has corrected QT interval using Fridericia's formula (QTcF) prolongation at rest, where the mean QTc interval is > 470 msec based on triplicate measurements of electrocardiogram (ECG);
    - History or current evidence of serious uncontrolled ventricular arrhythmias requiring drug therapy;
    - Left ventricular ejection fraction (LVEF) < 50% (for [redacted] [redacted], [redacted] Arm F subject only);
    - Congenital long QT syndrome, or any known history of torsade de pointes (TdP), or family history of unexplained sudden death;
    - Clinically uncontrolled hypertension (after standard antihypertensive treatment, systolic blood pressure ≥ 150 mmHg and/or diastolic blood pressure ≥ 100 mmHg).
4. Subject has a history of (non-infectious) pneumonitis/interstitial lung disease that required steroids or has current pneumonitis/interstitial lung disease or any active systemic infection including but not limited to severe acute respiratory syndrome coronavirus 2 (SARS-CoV-2) infection.
5. For China and Taiwan region sites only [redacted]
    - Subject with human immunodeficiency virus (HIV) infection (positive HIV 1/2 antibody), hepatitis B infection (positive hepatitis B surface antigen), or hepatitis C infection (quantitative HCV RNA result greater than the lower limit of detection of the assay).

    [redacted]
6. Subject has any history or evidence of substance abuse or medical, psychological or social conditions that may, in the opinion of the investigator, interfere with participation in the study or evaluation of the study results.
7. Subject has impaired gastrointestinal (GI) function or GI diseases that may significantly alter the absorption or metabolism of oral medications.
8. Other serious illness or medical conditions at the investigator's discretion, that may influence study results, including but not limited to serious infection, diabetes, cardiovascular and cerebrovascular accident (CVA) (<6 months before study entry) and lung disease.

9. Subject has unresolved toxicities from prior anticancer therapy, defined as toxicities (other than alopecia) not yet resolved to NCI CTCAE, v5.0, Grade ≤ 1 (Grade ≤ 2 for peripheral neuropathy).

   [redacted]

10. Subject had major surgery within 4 weeks prior to study treatment administration or last dose of palliative radiation therapy within 2 weeks prior to study treatment administration.
    - Subject must have recovered adequately from the surgery and/or any complications of the surgery prior to starting study treatment.
    - Subject must have recovered from all radiation-related toxicities, not requiring corticosteroids, and with no occurrence of radiation pneumonitis. A 1-week washout is permitted for palliative radiation that lasted ≤2 weeks for non-CNS disease.
11. Subject has received radiation therapy >30 Gy to the lung within 6 months of the first dose of study treatment
12. Subject with uncontrolled pleural effusion, pericardial effusion or ascites. A subject who is clinically stable following treatment for these conditions (including therapeutic thoraco- or paracentesis, indwelling catheters) is eligible.
13. Subject is currently receiving or planning to receive medications known to be substrate of CYP3A4 with a narrow therapeutic window, strong inducer or strong inhibitor of CYP3A4, with a known risk to prolong the QT interval or strong inhibitor of P-glycoprotein within 14 days or 5 half-lives of the drug or its major active metabolite, whichever is shorter, prior to study day 1 [redacted]
14. Subject has any other known primary malignancy that is progressing or has required active treatment within the past 3 years. Note: subjects with basal cell carcinoma of the skin, squamous cell carcinoma of the skin, or carcinoma in situ (e.g., breast carcinoma, cervical cancer in situ) that have undergone potentially curative therapy are not excluded.
15. Subject is pregnant or lactating.
16. [redacted]
    - [redacted]
    - [redacted]
    - [redacted]

- [redacted]
  - [redacted]
  - [redacted]
  - [redacted]
  - [redacted]

Study Design

This is a phase 1/2, open-label study of D-1553 single agent and in combination with [redacted] targeted therapy to assess the safety and tolerability, identify the MTD and RP2D, evaluate the PK properties and antitumor activities in subjects with advanced or metastatic solid tumor with $KRAS^{G12C}$ mutation. The study is divided into Phase 1 and Phase 2 portions. Phase 1 will consist of 2 parts: Phase 1a (dose escalation) and Phase 1b (dose combination). Subjects will take D-1553 orally on an empty stomach every day in a 21 or 28-day treatment cycle. The study treatment will be administered until disease progression, withdrawal of consent, unacceptable toxicity or discontinuation from the study for other reasons.

<u>Phase 1a (Dose Escalation)</u>

Phase 1a will start to evaluate 4 sequential cohorts administered with once daily oral doses of D-1553 (see table below): Cohort 1 (150 mg once-daily), Cohort 2 (300 mg once-daily), Cohort 3 (600 mg once-daily) and Cohort 4 (1200 mg once-daily). An additional 3 sequential cohorts administered with twice-daily oral doses of D-1553 will also be evaluated: Cohort 5 (400 mg twice-daily; total 800 mg daily), Cohort 6 (600 mg twice-daily; total 1200 mg daily) and Cohort 7 (800 mg twice-daily; total 1600 mg daily). After the safety of Cohort 3 has been determined, the dose escalation of the twice-daily administered cohorts (Cohorts 5 to 7) will be conducted in parallel to the once-daily administered cohort 4.

Dose Escalation Cohorts For Phase 1a

**Cohorts (once-daily)**	**Cohort 1**	**Cohort 2**	**Cohort 3**	**Cohort 4**		
**Dose**	150 mg once-daily	300 mg once-daily	600 mg once-daily	1200 mg once-daily		
**Cohorts (twice-daily)**				**Cohort 5**	**Cohort 6**	**Cohort 7**

	**Dose**				400 mg twice-daily	600 mg twice-daily	800 mg twice-daily

The starting dose of 150 mg once-daily in human was selected based on the information derived from nonclinical safety pharmacology and toxicology studies. This dose has been intentionally chosen as a conservative starting point to maximize safety margins according to the standard DeGeorge method, [redacted]

Accelerated titration (single subject per cohort) will be used for Cohorts 1 and 2 until one Grade 2 or higher at least possibly treatment-related adverse event (TRAE) occurs during the DLT observation period (treatment cycle 1), after which another 2 subjects will be enrolled in the same cohort and the dose escalation will be converted to follow conventional 3+3 design. If no Grade 2 or higher TRAE occurs in Cohorts 1 and 2, Cohort 3 will start to follow the conventional 3+3 design. Cohorts 5 to 7 will always follow the conventional 3+3 dose escalation design.

The conventional 3+3 dose escalation will be conducted as follows:

Three subjects will be enrolled and treated in the current cohort and observed for DLT during the DLT observation period (treatment cycle 1).

- If there is no DLT, the next higher dose cohort can start.
- If there is one DLT, another 3 subjects will be enrolled and treated.
  - If there is no more than one DLT in 6 subjects, the next higher dose cohort can start.
  - If there are more than one DLTs in 6 subjects, the MTD has been exceed and the next lower dose will be the MTD or an intermediate dose may be evaluated.
- If there are two or more DLTs, the MTD has been exceeded and the next lower dose will be the MTD or an intermediate dose may be evaluated.

This 3+3 dose escalation scheme will be repeated until the MTD is determined or the highest planned dose is reached.

Each subject will receive daily oral doses of D-1553 (once daily for Cohorts 1 to 4 and twice daily for Cohorts 5 to 7) from Day 1 to Day 21 on an empty stomach [redacted] Each 21-day period will be considered as 1 treatment cycle. Subjects may continue daily D-1553 treatment until disease progression, unacceptable treatment related toxicity, withdrawal of consent or discontinuation for any reason, whichever occurs first. Safety will be assessed throughout treatment cycles and during follow-up periods. Dose limiting toxicity will be evaluated during the 1st treatment cycle. Pharmacokinetic parameters (AUC_{0-t}, AUC_{inf}, MRT, C_{max}, t_{max}, C_{min}, $t_{½}$, V_{dss}/F and CL/F) will be evaluated during the 1st and 3rd treatment cycles. Pharmacokinetic timepoints of subsequent cohorts may be adjusted based on PK results from Cohort 1 and/or Cohort 2. Tumor assessments will be performed at screening and every 2 treatment cycles for the first 8 treatment cycles and every 3 treatment cycles thereafter until disease progression.

A Safety Review Committee (SRC), [redacted] will regularly assess the safety of D-1553 and its

combination therapy throughout the study. After completion of the 1st treatment cycle in each cohort of the conventional 3+3 dose escalation scheme, the SRC will review all available data to recommend whether or not to proceed to the next cohort. The SRC may recommend to lower the increment for the next dose level or add additional cohorts at the dose escalation meeting, based on the safety and PK results. Intermediate dose escalation levels (between Cohorts 1 and 4; Cohorts 5 and 7) may be evaluated during the study, as shown in the table below.

Intermediate Dose Escalation Cohorts for Phase 1a

**Cohorts (once daily)**	**Cohort 1**	**Cohort 2**	**Cohort 3**	**Cohort 4**		
Dose	150 mg once-daily	300 mg once-daily	600 mg once-daily	1200 mg once-daily		
Intermediate Dose	200 mg once-daily	450 mg once-daily	900 mg once-daily			
**Cohorts (twice-daily)**				**Cohort 5**	**Cohort 6**	**Cohort 7**
Dose				400 mg twice-daily	600 mg twice-daily	800 mg twice-daily
Intermediate Dose			300 mg twice-daily	500 mg twice-daily	700 mg twice-daily	

At the highest dose level or MTD, the cohort may be expanded to up to 10 subjects for further evaluation of safety, PK and antitumor activity. When a higher dose is determined to be safe, the previous lower dose cohort may be backfilled if further evaluation deems necessary. Subjects who completed the DLT evaluation period can receive a higher dose in subsequent cycles provided that they have tolerated the initial dose and the increased dose has been studied and proven safe. The RP2D (at or below MTD) of D-1553 as a single agent and the way it will be administered (once-daily or twice-daily) will be chosen based on SRC review of the safety and PK data at the completion of Phase 1a, for further evaluation in Phases 2 Arms A and B. This dose should be safe and has shown sufficient PK exposure and/or preliminary antitumor activity in Phase 1a result.

<u>Phase 1b (Dose Combination)</u>

Phase 1b will determine the MTD of D-1553 in combination with [redacted] in subjects with advanced or metastatic [redacted] There are 4 groups in Phase 1b for different tumor types and combinations:

- [redacted]

The enrollment to all groups ([redacted] will occur at the same time and each group will be evaluated independently. From Phase 1a results, if the RP2D of D-1553 single agent is the same as MTD, each group will have 2 dose level cohorts of D-1553 to assess the safety and tolerability of D-1553 in combination with [redacted] in

a conventional 3+3 design: Cohort 1 (1 dose level below RP2D of D-1553 single agent) and Cohort 2 (RP2D of D-1553 single agent). Three subjects will be enrolled in Cohort 1 first. If there is no DLT, Cohort 2 will be started. If there is 1 DLT, another 3 subjects will be enrolled in Cohort 1. If there is no DLT in the additional 3 subjects, Cohort 2 will be started. If at any time there are more than 1 DLTs in a cohort of 3-6 subjects, the MTD has been exceeded and the next lower dose level is declared to be the MTD and safe. A dose level below Cohort 1 dose level may be added as Cohort -1 for 3+3 scheme evaluation, if Cohort 1 level is determined to be unsafe.

From Phase 1a results, if the RP2D of D-1553 single agent is lower than MTD or the MTD is not reached at the highest dose, each group in Phase 1b will start with Cohort 2 (RP2D of D-1553 single agent) in a cohort of 3 subjects to evaluate the safety and PK of combination regimens.

The SRC will review the safety and PK data (if available) of each dose cohort, after completion of the 1st treatment cycle to determine whether or not to proceed to further expansion or de-escalate to the next level. The SRC may recommend to evaluate an intermediate dose level or up to two levels lower than RP2D of D-1553 single agent, based on analysis of the safety data. Once the MTD is determined, or the RP2D of D-1553 single agent is determined to be safe with the combination regimen, that dose level group ([redacted]) may proceed to Phase 2 in Arms [redacted] F, respectively. [redacted]

Each subject will receive an oral dose of D-1553 (once-daily or twice-daily) at the assigned dose, combined with [redacted] cetuximab in a 21-day cycle. Subjects may continue study treatment until disease progression, unacceptable treatment-related toxicity, withdrawal of consent or discontinuation from study for other reasons, whichever occurs first. [redacted] Safety will be assessed throughout treatment cycles and follow-up periods. Pharmacokinetic parameters (area under the concentration time curve (AUC), C_{max}, t_{max}, C_{min}, $t_{½}$, V_{dss}/F and CL/F) will be evaluated during the 1st and 3rd treatment cycles. Tumor assessments will be performed at screening, every 2 treatment cycles for the first 8 treatment cycles and every 3 treatment cycles thereafter until disease progression.

Once a PR or CR is observed, a confirmatory tumor assessment is recommended to be performed with at least 4 weeks' interval per RECIST 1.1.

Phase 2

The Phase 2 portion is a 6 arm, parallel, open-label study to evaluate the efficacy of D-1553 single agent and combination treatments in subjects with advanced or metastatic solid tumors with KRAS ^{G12C} mutation. [redacted] The futility criteria

are based on assumptions for the ORR for the standard of care (SOC) and the expected effect based on the addition of D-1553 to the SOC.

Simon 2-stage design futility criteria for [redacted]

The alternative vs null objective response rate rates (P1 vs P0)	Futility stage 1	Futility stage 2	Applies to
[redacted]	[redacted]	[redacted]	[redacted]
[redacted]	[redacted]	[redacted]	[redacted]
[redacted]	[redacted]	[redacted]	[redacted]

Arms [redacted] B are for single agent D-1553 treatment and Arms [redacted] F are for combination treatments, as follows:

- [redacted]
- Arm B: solid tumors other than NSCLC treated with D-1553 single agent at RP2D; [redacted]
- [redacted]
- [redacted]
- [redacted]
- Arm F: CRC, D-1553 in combination with cetuximab.

Upon completion of Phase 1a (single agent dose escalation), Phase 2 Arms [redacted] B may be started, while Phase 2 Arms [redacted] F may be started once Phase 1b (dose combination) is completed. The sequence of enrollment to each group or arm will be communicated to investigational sites by study team. [redacted] Each arm will enroll approximately 30 subjects (see table above) for evaluation of safety and efficacy in subjects with KRAS^{G12C} bearing solid tumors. If 1 or more arms demonstrate significant antitumor activity that warrants further evaluation, the protocol will be amended to allow further expansion of the arm(s).

Approximately 70 subjects will be enrolled in Phase 2 Arm B.

Approximately 40 subjects will be enrolled in Phase 2 Arm F.

Each subject will receive an oral dose of D-1553 at RP2D. Each 21- or 28-day period will be considered as 1 treatment cycle. Subjects may continue with daily administration of D-1553 until disease progression, unacceptable treatment-related toxicity, withdrawal of consent or discontinuation from the study for other reasons, whichever occurs first. In the combination arms, D-1553 may be continued at the same dose level after the combination regimen is completed, if the investigator considers it is in the best interest of the subject, after consulting with the sponsor's medical representative. Safety will be assessed throughout treatment cycles and during follow-up periods. PK samples will be collected in the 1st and 3rd treatment cycles. Tumor

	assessments will be performed at screening and after every 2 treatment cycles for the first 8 treatment cycles and every 3 treatment cycles thereafter until disease progression.<br><br>Once a PR or CR is observed, a confirmatory tumor assessment is recommended to be performed with at least 4 weeks' interval per RECIST 1.1.
**Sample Size Determination**	The total sample size of the study is anticipated at approximately 286 enrolled subjects. Based on whether earlier futility criteria are met in the Phase 2 arms, fewer subjects will be enrolled. If a much higher efficacy is observed after stage 1 or stage 2 in any of the arms, the protocol might be amended to further expand the population in the respective arm.<br><br>**<u>Phase 1a (Dose Escalation)</u>**<br><br>Approximately 27 subjects will be enrolled, assuming an average of 3 subjects per cohort and 6 subjects each at the highest dose cohort in the once-daily and twice-daily administered cohorts, in this 7-cohort dose escalation study.<br><br>**<u>Phase 1b (Dose Combination)</u>**<br><br>Subjects will be enrolled into 4 groups, each with approximately 9 subjects, assuming 2 dose levels are evaluated with up to 6 subjects at the highest dose group, except group 1. Approximately 13 subjects will be enrolled into group 1, assuming 2 dose levels are evaluated with up to 10 subjects at the highest dose group. A total of approximately 40 subjects will be enrolled in this 4-group dose combination study.<br><br>**<u>Phase 2</u>**<br><br>[redacted]<br><br>• [redacted]<br><br>Arm B: approximately 70 subjects<br><br>Arm F: approximately 40 subjects.<br><br>A total of up to 219 subjects will be enrolled in Phase 2.
**Statistical Methodology**	Descriptive statistics will be used throughout the study and described per dose level along with an overall assessment of the entire data.<br><br>**<u>Safety analysis</u>**<br><br>Number and percentage of subjects with occurrence of DLT during the observation period will be provided by dose level and overall. Descriptive statistics will be provided for AEs (according to NCI CTCAE v5.0), and for ECG parameters, physical examination, performance status (ECOG), vital signs and laboratory tests. Results will be presented by dose level and overall.

	**Pharmacokinetics analysis**<br><br>Phase 1a and 1b: Noncompartmental data analysis (determination of $t_{½}$, AUC_{0-t}, AUC_{inf}, MRT, V_{dss}/F, CL/F, C_{max}, t_{max}, C_{min}) will be performed on the individual plasma concentration-time data using actual times for D-1553 single agent or in combination with [redacted] to address potential drug-drug interaction. Descriptive statistics will be performed on the plasma concentrations and on the PK parameters of D-1553. Dose proportionality of D-1553 will be analyzed if data are sufficient. Pharmacokinetics of active metabolite(s) of D-1553 (if any) will be analyzed.<br><br>Phase 2: Based on PK data obtained in this study as well as PK data obtained from other studies, a population PK analysis will be performed to characterize pharmacokinetic parameters (Clearance (CL), Volume of distribution (V)) and evaluate the effect of extrinsic and intrinsic factors to support proposed dosing regimen. Pharmacokinetic data will also be used to explore the exposure-response relationships for D-1553 antitumor activity/efficacy as well as safety in the proposed patient population, if feasible. The results of these analyses, if performed, will be reported separately.<br><br>**Efficacy analysis**<br><br>Descriptive statistics will be provided for efficacy results in Phase 1.<br><br>The estimates of objective response rate and disease control rate and their 95% exact confidence intervals by Clopper-Pearson method will be reported. The Kaplan-Meier method will be used for analyzing PFS, OS and the DOR; median PFS, median OS, median DOR, and corresponding 95% confidence intervals of the medians will be computed as appropriate. The Kaplan-Meier curves will be plotted.
**Planned Number of Subjects**	The total sample size of the study is anticipated at approximately 286 enrolled subjects.
**Planned Number of Sites**	The total number of sites is expected to be approximately 20 - 50 sites.
**Estimated Time to Complete Enrollment**	Approximately 30 months.

1.2 Schema

Figure 1-1 Study Design for Phase 1

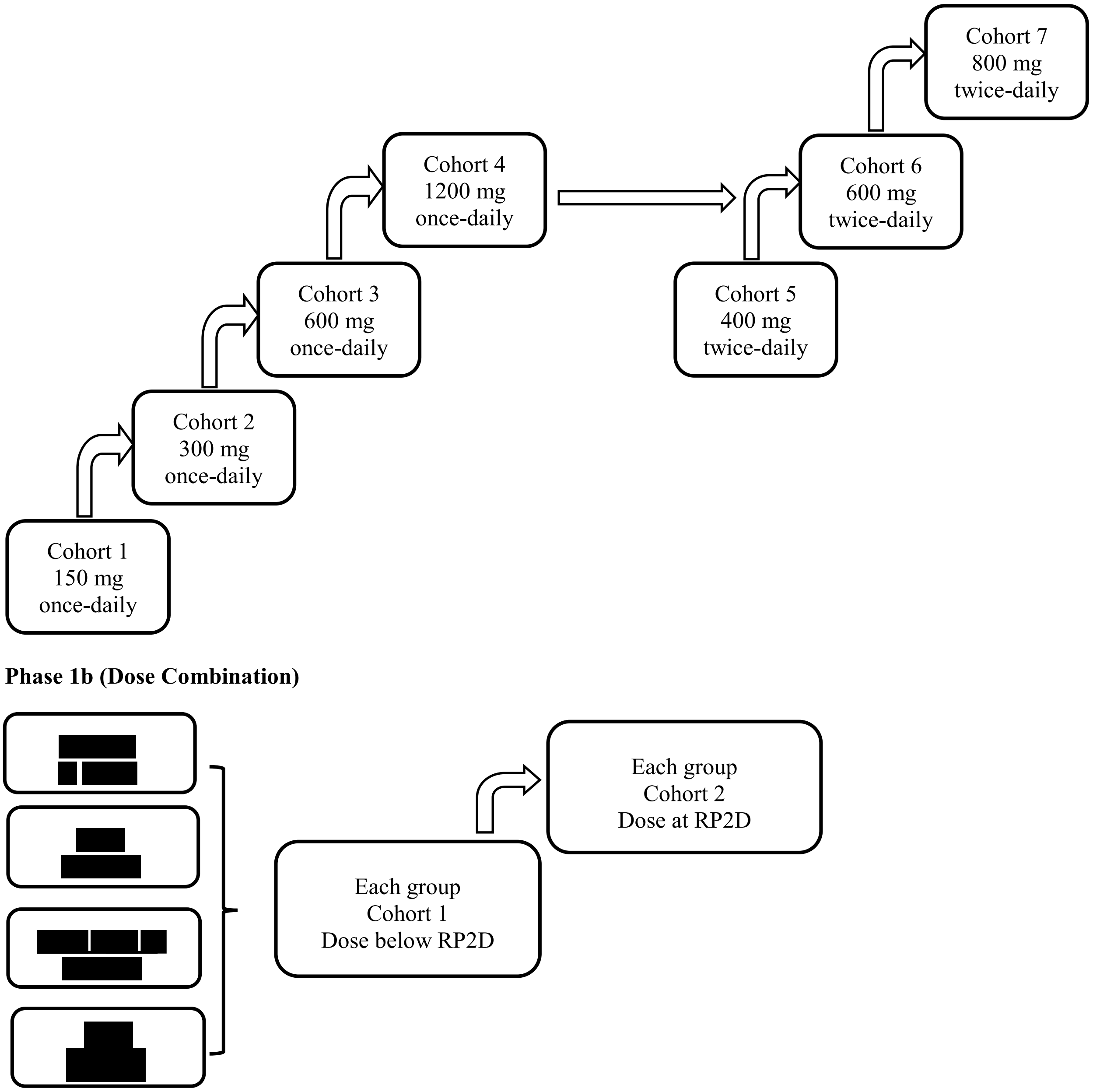

[redacted] RP2D: recommended phase 2 dose

Figure 1-2 Study Design for Phase 2

Phase 2

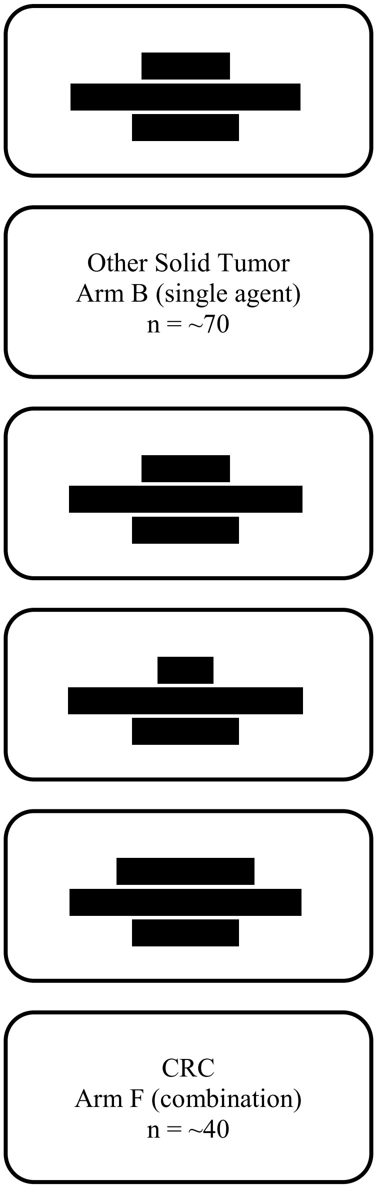

CRC: colorectal cancer[redacted]

Arm B: solid tumors other than NSCLC treated with D-1553 single agent

[redacted]

Arm F: CRC treated with D-1553 at combination RP2D with cetuximab

1.3 Schedule of Visits and Procedures

Table 1-1 Schedule of Activities for Phase 1a (Dose Escalation)

Procedures / Day / Visit Window	Screening (up to 28 days before Day 1)		Intervention Period: Treatment Cycle 1 (21-day treatment cycle)			Intervention Period: ≥ Treatment Cycle 2	End of Treatment or Withdrawal[18]	Follow-up: Safety (30 days after last dose)	Follow-up: PFS
**Day**	**-28 to -1**	**-1 to 1[17]**	**1**	**2**	**14**	**1**			
**Visit Window**	**NA**	**NA**	**0**	**0**	**± 4**	**± 4**	**+ 7**	**± 7**	**± 2 weeks**
Informed consent[1]	X								
Medical history[2]	X								
Diagnosis and history of solid tumor[3]	X								
Demographics[4]	X								
Physical examination[5]	X	X			X	X	X		
Inclusion and exclusion criteria[6]	X	X							
Pregnancy test[7] (WOCBP only)	X	X				X	X		
ECOG performance status[8]	X		X			X	X		
Assessment of KRAS^{G12C} status[9]	X								
Vital signs[10]	X	X	X	X	X	X	X		
Laboratory assessments[11] (hematology, blood chemistry, urinalysis, coagulation)	X	X			X	X	X		
12-lead ECG[12]	X		X		X	X	X		
[redacted]	X					X	X		
[redacted]	X	X				X	X		
D-1553[14]			X	X	X	X			

Procedures / Day / Visit Window	Screening (up to 28 days before Day 1)		Intervention Period: Treatment Cycle 1 (21-day treatment cycle)			Intervention Period: ≥ Treatment Cycle 2	End of Treatment or Withdrawal[18]	Follow-up: Safety (30 days after last dose)	Follow-up: PFS
Day	-28 to -1	-1 to 1[17]	1	2	14	1			
Visit Window	NA	NA	0	0	± 4	± 4	+ 7	± 7	± 2 weeks
Blood sample collection for PK[15]			X	X	X	X			
Tumor assessment[16]	X					X	X		X[16]
AE/SAE review		X	X	X	X	X	X	X	
Prior/concomitant medication review	X	X	X	X	X	X	X	X	

AE: adverse event; ALT: alanine aminotransferase; ALP: alkaline phosphatase; AST: aspartate aminotransferase; BUN: blood urea nitrogen; CNS: central nervous system; CRC: colorectal cancer; CT: computerized tomography; ECG: electrocardiogram; ECOG: Eastern Cooperative Oncology Group; INR: international normalized ratio; GGT: gamma-glutamyl transferase; LDH: lactate dehydrogenase; MRI: magnetic resonance imaging; NSCLC: non-small cell lung cancer; PK: pharmacokinetic(s); aPTT: activated partial thromboplastin time; RBC: red blood cell; RDE: recommended dose for expansion; SAE: serious adverse event, WBC: white blood cell; WOCBP: women of childbearing potential

1. Written informed consent should be obtained prior to any screening procedure or study procedure.
2. Medical history includes details of oncology history, smoke history, history of other diseases (active or resolved) and concomitant illnesses; should be collected within 28 days prior to first dose of study intervention.
3. History of solid tumor information must include previous treatment history and mutation information, if available.
4. Demographic data includes age, sex and race/ethnic group as indicated by the subject.
5. Physical examination includes major body systems. Body weight will be recorded at screening and Day 1 of each treatment cycle. Height will only be measured at Screening.
6. Inclusion and exclusion criteria must be evaluated prior to first dose of study intervention.
7. Serum or urine pregnancy tests will be performed at Screening, Pre-Dose Day -1 and End of Treatment or Withdrawal as well as on Day 1 of every odd-numbered cycle (except Cycle 1). Subjects with a positive pregnancy test will be permanently discontinued from treatment with study intervention and will enter into a safety follow-up period.
8. ECOG performance status grade is available in Appendix 1 of the protocol.

9. KRAS^{G12C} mutation status must be documented for the purpose of the inclusion criteria using a blood or tumor tissue sample and can be from a previously conducted local laboratory test result up to 5 years prior to this study. The method used for the test should also be collected.

10. Vital signs include body temperature, pulse, respiratory rate and blood pressure. These should be done once pre-dose and once post-dose on dosing days, and once on non-dosing days (pre- and post-dose procedures in BID cohort will only be required for the first dose during the day). Both pre-dose and post-dose measurements can have 1-hour window.

11. Laboratory assessments includes hematology, blood chemistry, urinalysis and coagulation. Weekly or more frequent tests may be given at the investigator's discretion:
    Hematology includes complete blood count including RBC, hemoglobin, hematocrit, WBC and differential (neutrophils, eosinophils, basophils, lymphocytes, monocytes and other cells) and platelet count. Blood chemistry includes total, direct and indirect bilirubin, ALT, AST, ALP, LDH, total protein, albumin, sodium, potassium, magnesium, chloride, calcium, phosphorus, BUN, creatinine, uric acid, glucose. Additional blood chemistry tests may be performed as clinically indicated (e.g., amylase, lipase, creatinine kinase, GGT). Urinalysis includes pH, specific gravity, protein, glucose, ketones, RBC, WBC, bilirubin. Additional analyses may be required as clinically indicated. Coagulation parameters should be determined throughout the study when clinically indicated, including aPTT and INR.

12. Triplicate measurements of 12-lead ECGs (recommended to perform 3 ECGs within 5 minutes) will be recorded during screening, at end of treatment, and at 2~6 hours post-dose on indicated study days and read by the central laboratory (pre- and post-dose procedures in BID cohort will only be required for the first dose during the day). The time of post-dose ECG is recommended to be as close as possible to the PK sampling time unless it's too difficult for clinical practice.

13. [redacted]

14. Each subject will receive daily oral doses of D-1553 (once daily for Cohorts 1 to 4 and twice daily for Cohorts 5 to 7) from Day 1 to Day 21 on an empty stomach (no food and drink, except for water, for at least 0.5 hours before and after dosing). Each 21day period will be considered as 1 treatment cycle. Subjects in the twice daily administered cohorts will be dosed with study intervention approximately 12 (± 3) hours apart. D-1553 will be dispensed on Day 1 of each cycle together with the instruction of taking the drug at home and the diary for recording of dose taken.

15. Blood samples will be collected for PK analysis of D-1553 at the following timepoints: pre-dose, 1 hour, 2 hours, 4 hours, 6 hours and 10 hours post-dose on Day 1 of treatment cycle 1, Day 14 of treatment cycle 1, and Day 1 of treatment cycle 3; pre-dose on Day 2 of treatment cycle 1.

16. Radiologic imaging will be used for tumor assessment. A CT scan (with or without contrast) of the chest, abdomen and pelvis will be performed to assess metastatic disease. A bone scan will be performed in the event of clinical symptoms of bone metastases. A baseline brain MRI (with or without contrast; CT is acceptable if MRI is contradicted), should be performed for subjects with a history of or suspected to have CNS metastases. For subjects with no CNS metastases at baseline, it is not necessary to repeat brain MRI unless CNS involvement is suspected during the study. Screening scans can be performed within 6 weeks prior to Day 1 of treatment cycle 1. Tumor assessment will be performed during screening and at the completion of every 2 treatment cycles for the first 8 treatment cycles, thereafter at the end of every 3 treatment cycles until disease progression, start of new anticancer treatment, death, withdrawal of consent, or until end of study. There is a ± 7-day window for each tumor assessment. For PFS, subjects who do not have disease progression on study will be followed for tumor assessment, approximately every 12 weeks (± 2 weeks), until disease progression, death, lost to follow-up, start of another anticancer therapy, or other reasons that a follow-up is not possible.

17. These procedures can be done on Day -1 or Day 1 but have to be completed prior to first dose of study intervention. They are intended for checking the subject status before the first dose, not for eligibility. A subject's eligibility should be determined during the screening period. Laboratory tests performed in the screening period within 7 days of Day 1 do not need to be repeated on Day -1 or Day 1 unless clinically indicated.

18. These visits need to be completed within 7 days of the last dose of study intervention:
    a. Subjects who stop treatment due to disease progression at the visit time do not need to repeat the imaging examination;
    b. The radiologic imaging examination needs to be repeated if more than 4 weeks have passed from the last examination to the end of treatment visit;
    c. Other examinations are required to be repeated if more than 1 week from last examinations.

19. [redacted]

Table 1-2 Schedule of Activities for Phase 1b (Dose Combination)

	Screening (up to 28 days before Day 1)		Intervention Period					End of Treatment or Withdrawal[23]	Follow-up	
			Treatment Cycle 1 (21 or 28-day treatment cycle)				≥ Treatment Cycle 2		Safety (30 days after last dose)	PFS
**Day**	**-28 to -1**	**-1 to 1[22]**	**1**	**2**	**8**	**14**	**1**			
**Procedures** **Visit Window**	**NA**	**NA**	**0**	**0**	**± 2**	**± 4**	**± 4**	**+ 7**	**± 7**	**± 2 weeks**
Informed consent[1]	X									
Medical history[2]	X									
Diagnosis and history of solid tumor[3]	X									
Demographics[4]	X									
Physical examination[5]	X	X				X	X	X		
Inclusion and exclusion criteria[6]	X	X								
Pregnancy test[7] (WOCBP only)	X	X					X	X		
ECOG performance status[8]	X		X				X	X		
Assessment of KRAS^{G12C} status[9]	X									
Vital signs[10]	X	X	X	X		X	X	X		
Laboratory assessments[11] (hematology, blood chemistry, urinalysis, coagulation)	X	X			X	X	X	X		
HIV, Hep B and Hep C[24]	X									
12-lead ECG[12]	X		X			X	X	X		
[redacted] [redacted]	X						X	X		
[redacted] [redacted]	X	X					X	X		
[redacted]	X		X				X	X		
D-1553[16]			X	X	X	X	X			

	**Screening (up to 28 days before Day 1)**		**Intervention Period**					**End of Treatment or Withdrawal** [23]	**Follow-up**	
			**Treatment Cycle 1 (21 or 28-day treatment cycle)**				**≥ Treatment Cycle 2**		**Safety (30 days after last dose)**	**PFS**
**Day**	**-28 to -1**	**-1 to 1** [22]	**1**	**2**	**8**	**14**	**1**			
**Procedures** **Visit Window**	**NA**	**NA**	**0**	**0**	**± 2**	**± 4**	**± 4**	**+ 7**	**± 7**	**± 2 weeks**
[redacted] [redacted]			X				X			
[redacted] [redacted]			X				X			
[redacted] [redacted]			X	X	X	X	X			
Cetuximab for CRC[19]			X		X	X	X			
Blood sample collection for PK[20]			X	X		X	X			
Tumor assessment[21]	X						X	X		X[21]
AE/SAE review		X	X	X	X	X	X	X	X	
Prior/concomitant medication review	X	X	X	X	X	X	X	X	X	

AE: adverse event; ALT: alanine aminotransferase; ALP: alkaline phosphatase; AST: aspartate aminotransferase; BUN: blood urea nitrogen; CNS: central nervous system; CRC: colorectal cancer; CT: computerized tomography; ECG: electrocardiogram; ECOG: Eastern Cooperative Oncology Group; INR: international normalized ratio; GGT: gamma-glutamyl transferase; LDH: lactate dehydrogenase; MRI: magnetic resonance imaging; NSCLC: non-small cell lung cancer; PK: pharmacokinetic(s); aPTT: activated partial thromboplastin time; RBC: red blood cell; RDE: recommended dose for expansion; SAE: serious adverse event, WBC: white blood cell; WOCBP: women of childbearing potential

1. Written informed consent should be obtained prior to the performance of any screening procedure or study procedure.
2. Medical history includes details of oncology history, smoke history, history of other diseases (active or resolved) and concomitant illnesses; should be collected within 28 days prior to first dose of study intervention.
3. History of solid tumor information must include previous treatment history and mutation information, if available.
4. Demographic data includes age, sex and race/ethnic group as indicated by the subject.

C Confidential

5. Physical examination includes major body systems. Body weight will be recorded at Screening and Day 1 of each treatment cycle. Height will only be measured at screening.

6. Inclusion and exclusion criteria must be evaluated prior to first dose of study intervention.

7. Serum or urine pregnancy tests will be performed at Screening, Pre-Dose Day -1 and End of Treatment or Withdrawal as well as on Day 1 of every odd-numbered cycle (except Cycle 1). Subjects with a positive pregnancy test will be permanently discontinued from treatment with study intervention and will enter into a safety follow-up period.

8. ECOG performance status grade is available in Appendix 1 of the protocol

9. $KRAS^{G12C}$ mutation status must be documented for the purpose of the inclusion criteria using a blood or tumor tissue sample and can be from a previously conducted local laboratory test result up to 5 years prior to this study. The method used for the test should also be collected.

10. Vital signs include body temperature, pulse, respiratory rate and blood pressure. These should be done once pre-dose and once post-dose on dosing days, and once on non-dosing days (pre-and post-dose procedures in BID cohort will only be required for the first dose during the day). Both pre-dose and post-dose measurements can have 1-hour window.

11. Laboratory assessments include hematology, blood chemistry, urinalysis and coagulation. Weekly or more frequent tests may be given at the investigator's discretion:
    Hematology includes complete blood count including RBC, hemoglobin, hematocrit, WBC and differential (neutrophils, eosinophils, basophils, lymphocytes, monocytes and other cells) and platelet count. Blood chemistry includes total, direct and indirect bilirubin, ALT, AST, ALP, LDH, total protein, albumin, sodium, potassium, magnesium, chloride, calcium, phosphorus, BUN, creatinine, uric acid, glucose. Additional blood chemistry tests may be performed as clinically indicated (e.g., amylase, lipase, creatinine kinase, GGT). Urinalysis includes pH, specific gravity, protein, glucose, ketones, RBC, WBC, bilirubin. Additional analyses may be required as clinically indicated. Coagulation parameters should be determined throughout the study when clinically indicated or warfarin is to be administered, including aPTT and INR.

12. Triplicate measurements of 12-lead ECGs (recommended to perform 3 ECGs within 5 minutes) will be recorded during screening, at end of treatment, and at 1~4 hours post-dose on the indicated study days and read by the central laboratory (pre- and post-dose procedures in BID cohort will only be required for the first dose during the day). The time for post-dose ECG is recommended to be as close as possible to the PK sampling time unless it's too difficult for clinical practice.

13. [redacted]

14. [redacted]

C Confidential

15. [redacted]

16. Each subject will receive an oral dose of D-1553 (once daily or BID) at the assigned dose on an empty stomach (no food and drink, except for water, for at least 0.5 hours before and after dosing), combined with [redacted] cetuximab in a 21-day or 28-day treatment cycle depending on the combination therapy. Subjects may continue daily D-1553 until disease progression, unacceptable treatment related toxicity, withdrawal of consent or discontinuation for any reason, whichever occurs first. D-1553 will be dispensed on Day 1 of each cycle together with the instruction of taking the drug at home and the diary for recording of dose taken.

17. [redacted]

18. [redacted]

19. [redacted] Subjects will receive cetuximab: 400 mg/m^2 initial dose (120-minute IV infusion on Cycle1 Day 1), then 250 mg/m^2 (60-minute IV infusion) once weekly. Thereafter, each 21-day cycle will be considered as 1 treatment cycle. D-1553 and cetuximab may continue until disease progression, unacceptable treatment related toxicity, withdrawal of consent or discontinuation from study for other reasons, whichever occurs first.

20. Blood samples will be collected for PK analysis of D-1553 at the following timepoints: pre-dose, 1 hour, 2 hours, 4 hours, 6 hours and 10 hours post-dose on Day 1 of treatment cycle 1, Day 14 of treatment cycle 1, and Day 1 of treatment cycle 3, pre-dose on Day 2 of treatment cycle 1.

C Confidential

21. Radiologic imaging will be used for tumor assessment. A CT scan (with or without contrast) of the chest, abdomen and pelvis will be performed to assess metastatic disease. A bone scan will be performed in the event of clinical symptoms of bone metastases. A baseline brain MRI (with or without contrast; CT is acceptable if MRI is contradicted), should be performed for subjects with a history of or suspected to have CNS metastases. For subjects with no CNS metastases at baseline, it is not necessary to repeat brain MRI unless CNS involvement is suspected during the study. Screening scans can be performed within 6 weeks prior to Day 1 of treatment cycle 1. Tumor assessment will be performed at screening and every 2 treatment cycles for the first 8 treatment cycles, thereafter every 3 treatment cycles until disease progression, start of new anticancer treatment, death, withdrawal of consent, or until end of study. There is a ± 7-day window for each tumor assessment. For PFS, subjects who do not have disease progression on study will be followed for tumor assessment, approximately every 12 weeks (± 2 weeks), until disease progression, death, lost to follow-up, start of another anticancer therapy, or other reasons that a follow-up is not possible.

22. These procedures can be done on Day -1 or Day 1 but have to be completed prior to first dose of study intervention. They are intended for checking the subject status before the first dose, not for eligibility. A subject's eligibility should be determined during the screening period. Laboratory tests performed in the screening period within 7 days of Day 1 do not need to be repeated on Day -1 or Day 1 unless clinically indicated.

23. These visits need to be completed within 7 days of the last dose of study intervention:
    a. Subjects who stop treatment due to disease progression at the visit time do not need to repeat the imaging examination.
    b. The radiologic imaging examination needs to be repeated if more than 4 weeks have passed from the last examination to the end of treatment visit.
    c. Other examinations are required to be repeated if more than 1 week from last examinations.

24. Testing for HIV, Hep B and Hep C should be conducted during Screening for eligibility determination. A positive HBsAg will be considered positive for Hep B. A positive anti-HCV antibody and anti-HIV antibody will be considered positive for Hep C and HIV, respectively[redacted]

Confidential

Table 1-3 Schedule of Activities for Phase 2: [redacted] Arm B (Single Agent)

Procedures	Screening (up to 28 days before Day 1)		Intervention Period		End of Treatment or Withdrawal[19]	Follow-up		
			Treatment Cycle 1	≥ Treatment Cycle 2		Safety (30 days after last dose)	PFS	OS
Day	-28 to -1	-1 to 1[18]	1	1				
Visit Window	NA	NA	0	± 4	+ 7	+ 7	± 2 weeks	± 2 weeks
Informed consent[1]	X							
Medical history[2]	X							
Diagnosis and history of solid tumor[3]	X							
Demographics[4]	X							
Physical examination[5]	X	X		X	X			
Inclusion and exclusion criteria[6]	X	X						
Pregnancy test[7] (WOCBP only)	X	X		X	X			
ECOG performance status[8]	X		X	X	X			
Assessment of $KRAS^{G12C}$ status[9]	X							
Vital signs[10]	X	X	X	X	X			
Laboratory assessments[11] (hematology, chemistry, urinalysis, coagulation)	X	X		X	X			
12-lead ECG[12]	X		X	X	X			
[redacted]	X			X	X			
[redacted]	X	X		X	X			
HIV, Hep B and Hep C[26]	X							
D-1553[14]			X	X				
Blood sample collection for PK[15]			X	X				
Tumor assessment[16]	X			X	X		X[21]	
Futility assessment[17]				X				

Confidential

Procedures	Screening (up to 28 days before Day 1)		Intervention Period		End of Treatment or Withdrawal[19]	Follow-up		
			Treatment Cycle 1	≥ Treatment Cycle 2		Safety (30 days after last dose)	PFS	OS
Day	-28 to -1	-1 to 1[18]	1	1				
Visit Window	NA	NA	0	± 4	+ 7	+ 7	± 2 weeks	± 2 weeks
AE/SAE review		X	X	X	X	X		
Prior/concomitant medication review	X	X	X	X	X	X		
Telephone follow-up						X[20]		X[22]
Plasma ctDNA[24]	X			X	X			
archived tumor tissue (FFPE) for solid tumors[25]	X							

AE: adverse event; ALT: alanine aminotransferase; ALP: alkaline phosphatase; AST: aspartate aminotransferase; BUN: blood urea nitrogen; CNS: central nervous system; CRC: colorectal cancer; CT: computerized tomography; ECG: electrocardiogram; ECOG: Eastern Cooperative Oncology Group; INR: international normalized ratio; GGT: gamma-glutamyl transferase; LDH: lactate dehydrogenase; MRI: magnetic resonance imaging; NSCLC: non-small cell lung cancer; PK: pharmacokinetic(s); aPTT: activated partial thromboplastin time; RBC: red blood cell; RDE: recommended dose for expansion; SAE: serious adverse event, SOC: standard of care; WBC: white blood cell; WOCBP: women of childbearing potential

1. Written informed consent should be obtained prior to the performance of any screening procedure or study procedure.
2. Medical history includes details of oncology history, smoke history, history of other diseases (active or resolved) and concomitant illnesses; should be collected within 28 days prior to first dose of study intervention.
3. History of solid tumor information must include previous treatment history and mutation information, if available.
4. Demographic data includes age, sex and race/ethnic group as indicated by the subject.
5. Physical examination includes major body systems. Body weight will be recorded at screening and Day 1 of each treatment cycle. Height will only be measured at screening.
6. Inclusion and exclusion criteria must be evaluated prior to first dose of study intervention.

7. Serum or urine pregnancy tests will be performed at Screening, Pre-Dose Day -1 and End of Treatment or Withdrawal, as well as on Day 1 of every odd-numbered cycle (except Cycle 1). Subjects with a positive pregnancy test will be permanently discontinued from treatment with study intervention and will enter into a safety follow-up period.

8. ECOG performance status grade is available in Appendix 1 of the protocol.

9. $KRAS^{G12C}$ mutation status must be documented for the purpose of the inclusion criteria using a blood and tumor tissue sample tested in the central laboratory [redacted]

10. Vital signs include body temperature, pulse, respiratory rate and blood pressure. These should be done once pre-dose and once post-dose on dosing days, and once on non-dosing days (pre- and post-dose procedures in BID cohort will only be required for the first dose during the day). Both pre-dose and post-dose measurements can have 1-hour window.

11. Laboratory assessments include hematology, blood chemistry, urinalysis and coagulation. Weekly or more frequent tests may be given at the investigator's discretion:
Hematology includes complete blood count including RBC, hemoglobin, hematocrit, WBC and differential (neutrophils, eosinophils, basophils, lymphocytes, monocytes and other cells) and platelet count. Blood chemistry includes total, direct and indirect bilirubin, ALT, AST, ALP, LDH, total protein, albumin, sodium, potassium, magnesium, chloride, calcium, phosphorus, BUN, creatinine, uric acid, glucose. Additional blood chemistry tests may be performed as clinically indicated (e.g., amylase, lipase, creatinine kinase, GGT). Urinalysis includes pH, specific gravity, protein, glucose, ketones, RBC, WBC, bilirubin. Additional analyses may be required as clinically indicated. Coagulation parameters should be determined throughout the study when clinically indicated, including PTT and INR

12. Triplicate measurements of 12-lead ECGs (recommended to perform 3 ECGs within 5 minutes) will be recorded during screening, pre-dose on Day 1 of each Cycle, at end of treatment, and post-dose on the indicated study days (refer to Table 1-6) (pre- and post-dose procedures in BID cohort will only be required for the first dose during the day) and read by the central laboratory. The time for post-dose ECG is recommended to be as close as possible to the PK sampling time unless it's too difficult for clinical practice.

13. [redacted]

14. Each subject will receive an oral dose of D-1553 (once daily or BID) at RP2D on an empty stomach (no food and drink, except for water, for at least 0.5 hours before and after dosing) from Day 1 to Day 21. Each 21-day period will be considered as 1 treatment cycle. Subjects may continue with once daily or BID administration of D-1553 until disease progression, unacceptable treatment related toxicity, withdrawal of consent or discontinuation from the study for other reasons, whichever occurs first. D-1553 will be dispensed on Day 1 of each cycle together with the instruction of taking the drug at home and the diary for recording of dose taken.

15. At selected sites, up to a total of 30 phase 2 subjects will also be consented to have intensive PK and ECG data collected (Table 1-6). Sparse PK sampling will be collected in the other subjects (Table 1-6).

16. Radiologic imaging will be used for tumor assessment. A CT scan (with or without contrast) of the chest, abdomen and pelvis will be performed to assess metastatic disease. A bone scan will be performed in the event of clinical symptoms of bone metastases. A baseline brain MRI (with or without contrast; CT is acceptable if MRI is contradicted), should be performed for subjects with a history of or suspected to have CNS metastases. For subjects with no CNS metastases at baseline, it is not necessary to repeat brain MRI unless CNS involvement is suspected during the study. Screening scans can be performed within 6 weeks prior to Day 1 of treatment cycle 1. Tumor assessment will be performed at screening and the completion of every 2 treatment cycles for the first 8 treatment cycles, thereafter the completion of every 3 treatment cycles until disease progression, start of new anticancer treatment, death, withdrawal of consent, or until end of study. There is a ± 7-day window for each tumor assessment.

17. [redacted]

18. These procedures can be done on Day -1 or Day 1 but have to be completed prior to first dose of study intervention. They are intended for checking the subject status before the first dose, not for eligibility. A subject's eligibility should be determined during the screening period. Laboratory tests performed in the screening period within 7 days of Day 1 do not need to be repeated on Day -1 or Day 1 unless clinically indicated.

19. These visits need to be completed within 7 days of the last dose of study intervention:

    a. Subjects who stop treatment due to disease progression at the visit time do not need to repeat the imaging examination;

    b. The radiologic imaging examination needs to be repeated if more than 4 weeks have passed from the last examination to the end of treatment visit;

    c. Other examinations are required to be repeated if more than 1 week from last examinations.

20. A follow-up phone call is allowed to perform the check on AE status and concomitant medications.

21. Subjects who do not have disease progression on study will be followed for tumor assessment, approximately every 12 weeks (± 2 weeks), until disease progression, death, lost to follow-up, start of another anticancer therapy, or other reasons that a follow-up is not possible.

22. Subjects will be followed for survival status monthly (± 2 weeks), until death, lost to follow-up or other reasons that a follow-up is not possible. The follow-up can be done via telephone call.

23. [redacted]

24. Plasma ctDNA is to be collected pre-dose as indicated in the Schedule of Assessments (Screening, Day 1 of Cycle ≥ 2 and EOT). [redacted]

C Confidential

25. Subjects will provide archived FFPE samples (collected within 5 years) for $KRAS^{G12C}$ mutation confirmation that enables $KRAS^{G12C}$ testing prior to starting all other screening procedures.

26. Testing for HIV, Hep B and Hep C should be conducted during Screening for eligibility determination. A positive HBsAg will be considered positive for Hep B. A positive anti-HCV antibody and anti-HIV antibody will be considered positive for Hep C and HIV, respectively. [redacted]

C Confidential

Table 1-4 Schedule of Activities for Phase 2: [redacted] Arm F (Combination)

Procedures	Screening (up to 28 days before Day 1)		Intervention Period: Treatment Cycle 1 (21 or 28-day treatment cycle)			Intervention Period: ≥ Treatment Cycle 2	End of Treatment or Withdrawal[24]	Follow-up: Safety (30 days after last dose)	Follow-up: PFS	Follow-up: OS
Day	-28 to -1	-1 to 1[23]	1	2	8	1				
Visit Window	0	0	0	0	± 2	± 4	+7	+7	± 2 weeks	± 2 weeks
Informed consent[1]	X									
Medical history[2]	X									
Diagnosis and history of solid tumor[3]	X									
Demographics[4]	X									
Physical examination[5]	X	X				X	X			
Inclusion and exclusion criteria[6]	X	X								
Pregnancy test[7] (WOCBP only)	X	X				X	X			
ECOG performance status[8]	X		X			X	X			
Assessment of $KRAS^{G12C}$ status[9]	X									
Vital signs[10]	X	X	X	X		X	X			
Laboratory assessments[11] (hematology, blood chemistry, urinalysis, coagulation)	X	X			X	X	X			
HIV, Hep B and Hep C[28]	X									
12-lead ECG[12]	X		X			X	X			
[redacted]	X					X	X			
[redacted]	X	X				X	X			

Confidential

	**Screening (up to 28 days before Day 1)**		**Intervention Period**				**End of Treatment or Withdrawal**[24]	**Follow-up**		
			**Treatment Cycle 1 (21 or 28-day treatment cycle)**			**≥ Treatment Cycle 2**		**Safety (30 days after last dose)**	**PFS**	**OS**
**Day**	**-28 to -1**	**-1 to 1**[23]	**1**	**2**	**8**	**1**				
**Procedures Visit Window**	**0**	**0**	**0**	**0**	**± 2**	**± 4**	**+7**	**+7**	**± 2 weeks**	**± 2 weeks**
LVEF measurement[15]	X		X			X	X			
D-1553[16]			X	X	X	X				
[redacted]			X			X				
[redacted]			X			X				
[redacted]			X	X		X				
Cetuximab for CRC[19]			X		X	X				
Blood sample collection for PK[20]			X			X				
Tumor assessment[21]	X					X	X		X[26]	
Futility assessment[22]						X				
AE/SAE review		X	X	X	X	X	X	X		
Prior/concomitant medication review	X	X	X	X	X	X	X	X		
Telephone follow-up								X[25]		X[27]
Plasma ctDNA[29]	X					X	X			
archived tumor tissue (FFPE) for solid tumors[30]	X									

AE: adverse event; ALT: alanine aminotransferase; ALP: alkaline phosphatase; AST: aspartate aminotransferase; BUN: blood urea nitrogen; CNS: central nervous system; CRC: colorectal cancer; CT: computerized tomography; ECG: electrocardiogram; ECOG: Eastern Cooperative Oncology Group; INR: international normalized ratio; GGT: gamma-glutamyl transferase; LDH: lactate dehydrogenase; MRI: magnetic resonance imaging; [redacted]

C Confidential

PK: pharmacokinetic(s); aPTT: activated partial thromboplastin time; RBC: red blood cell; RDE: recommended dose for expansion; SAE: serious adverse event, SOC: standard of care; WBC: white blood cell; WOCBP: women of childbearing potential

1. Written informed consent should be obtained prior to the performance of any screening procedure or study procedure.
2. Medical history includes details of oncology history, smoke history, history of other diseases (active or resolved) and concomitant illnesses; should be collected within 28 days prior to first dose of study intervention.
3. History of solid tumor information must include previous treatment history and mutation information, if available.
4. Demographic data includes age, sex and race/ethnic group as indicated by the subject.
5. Physical examination includes major body systems. Body weight will be recorded at screening and Day 1 of each treatment cycle. Height will only be measured at screening.
6. Inclusion and exclusion criteria must be evaluated prior to first dose of study intervention.
7. Serum or urine pregnancy tests will be performed at Screening, Pre-Dose Day -1 and End of Treatment or Withdrawal, as well as on Day 1 of every odd-numbered cycle (except Cycle 1). Subjects with a positive pregnancy test will be permanently discontinued from treatment with study intervention and will enter into a safety follow-up period.
8. ECOG performance status grade is available in Appendix 1 of the protocol.
9. $KRAS^{G12C}$ mutation status must be documented for the purpose of the inclusion criteria using a blood and tumor tissue sample tested in the central laboratory and this result is not necessary for eligibility.
10. Vital signs include body temperature, pulse, respiratory rate and blood pressure. These should be done once pre-dose and once post-dose on dosing days, and once on non-dosing days (pre- and post-dose procedures in BID cohort will only be required for the first dose during the day). Both pre-dose and post-dose measurements can have ± 1-hour window.
11. Laboratory assessments include hematology, blood chemistry, urinalysis and coagulation. Weekly or more frequent tests may be given at the investigator's discretion:
    Hematology includes complete blood count including RBS, hemoglobin, hematocrit, WBC and differential (neutrophils, eosinophils, basophils, lymphocytes, monocytes and other cells) and platelet count. Blood chemistry includes total, direct and indirect bilirubin, ALT, AST, ALP, LDH, total protein, albumin, sodium, potassium, magnesium, chloride, calcium, phosphorus, BUN, creatinine, uric acid, glucose. Additional blood chemistry tests may be performed as clinically indicated (e.g., amylase, lipase, creatinine kinase, GGT). Urinalysis includes pH, specific gravity, protein, glucose, ketones, RBC, WBC, bilirubin. Additional analyses may be required as clinically indicated. [redacted]

Confidential

12. Triplicate measurements of 12-lead ECGs (recommended to perform 3 ECGs within 5 minutes) will be recorded during screening, pre-dose on Day 1 of each Cycle, at end of treatment, and post-dose on the indicated study days (refer to Table 1-6) (pre- and post-dose procedures in BID cohort will only be required for the first dose during the day) and read by the central laboratory. The time for post-dose ECG is recommended to be as close as possible to the PK sampling time unless it's too difficult for clinical practice.

13. [redacted]

14. [redacted]

15. LVEF measurement with Echocardiogram only applicable to Arm [redacted] F. LVEF is examined with echocardiography, once every 4 cycles (12 weeks) since C1D1. The examination may be exempt if echocardiography conducted in the screening period is within 28 days prior to C1D1 (including prior to signing an ICF). The examination at end-of-treatment visit may be exempt if echocardiography conducted is within 28 days prior to end-of-treatment.

16. Each subject will receive an oral dose of D-1553 (once daily or twice daily) at RP2D on an empty stomach (no food and drink, except for water, for at least 0.5 hours before and after dosing), combined with [redacted] cetuximab in a 21-day or 28-day treatment cycle, depending on the combination therapy. Subjects may continue with once daily or BID administration of D-1553 until disease progression, unacceptable treatment related toxicity, withdrawal of consent or discontinuation from the study for other reasons, whichever occurs first. D-1553 will be dispensed on Day 1 of each cycle together with the instruction of taking the drug at home and the diary for recording of dose taken.

17. [redacted]

18. [redacted]

Confidential

19. [redacted] Subjects will receive cetuximab: 400 mg/m^2 initial dose (120-minute IV infusion on Cycle 1 Day 1), then 250 mg/m^2 (60-minute IV infusion) once weekly. Thereafter, each 21-day cycle will be considered as 1 treatment cycle. D-1553 and cetuximab may continue until disease progression, unacceptable treatment related toxicity, withdrawal of consent or discontinuation from study for other reasons, whichever occurs first.

20. At selected sites, up to a total of 30 phase 2 subjects will also be consented to have intensive PK and ECG data collected (Table 1-6). Sparse PK sampling will be collected in the other subjects (Table 1-6).

21. Radiologic imaging will be used for tumor assessment. A CT scan (with or without contrast) of the chest, abdomen and pelvis will be performed to assess metastatic disease. A bone scan will be performed in the event of clinical symptoms of bone metastases. A baseline brain MRI (with or without contrast; CT is acceptable if MRI is contradicted), should be performed for subjects with a history of or suspected to have CNS metastases. For subjects with no CNS metastases at baseline, it is not necessary to repeat brain MRI unless CNS involvement is suspected during the study. Screening scans can be performed within 6 weeks prior to Day 1 of treatment cycle 1. Tumor assessment will be performed at screening and at the completion of every 2 treatment cycles for the first 8 treatment cycles, thereafter at the completion of every 3 treatment cycles until disease progression, start of new anticancer treatment, death, withdrawal of consent, or until end of study. There is a ± 7-day window for each tumor assessment.

22. [redacted]

23. These procedures can be done on Day -1 or Day 1 but have to be completed prior to first dose of study intervention. They are intended for checking the subject status before the first dose, not for eligibility. A subject’s eligibility should be determined during the screening period. Laboratory tests performed in the screening period within 7 days of Day 1 do not need to be repeated on Day -1 or Day 1 unless clinically indicated.

24. These visits need to be completed within 7 days of the last dose of study intervention:

    a. Subjects who stop treatment due to disease progression at the visit time do not need to repeat the imaging examination;

    b. The radiologic imaging examination needs to be repeated if more than 4 weeks have passed from the last examination to the end of treatment visit;

    c. Other examinations are required to be repeated if more than 1 week from last examinations.

25. A follow-up phone call is allowed to perform the check on AE status and concomitant medications.

26. Subjects who do not have disease progression on study will be followed for tumor assessment, approximately every 12 weeks (± 2 weeks), until disease progression, death, lost to follow-up, start of another anticancer therapy, or other reasons that a follow-up is not possible.

Confidential

27. Subjects will be followed for survival status monthly (± 2 weeks), until death, lost to follow-up or other reasons that a follow-up is not possible. The follow-up can be done via telephone call.
28. Testing for HIV, Hep B and Hep C should be conducted during Screening for eligibility determination. A positive HBsAg will be considered positive for Hep B. A positive anti-HCV antibody and anti-HIV antibody will be considered positive for Hep C and HIV, respectively. [redacted]
29. Plasma ctDNA is to be collected pre-dose as indicated in the Schedule of Assessments (Screening, Day 1 of Cycle ≥ 2 and EOT). [redacted]
30. Subjects will provide archived FFPE samples (collected within 5 years) for KRAS p.G12C mutation confirmation that enables $KRAS^{G12C}$ testing prior to starting all other screening procedures.

C Confidential

Table 1-5 Plasma Pharmacokinetic Blood Sampling for Phase 1

	Treatment Cycle 1: Day 1 and Day 14; Treatment Cycle 3: Day 1						Treatment Cycle 1: Day 2
	Pre-dose	1 hour post-dose	2 hours post-dose	4 hours post-dose	6 hours post-dose	10 hours post -dose	Pre-dose
	- 1 to 0 hours	± 10 minutes	± 10 minutes	± 0.5 hours	± 0.5 hours	± 2 hours	- 1 to 0 hours
**D-1553***	X	X	X	X	X	X	X
[redacted]	[redacted]	[redacted]	[redacted]	[redacted]	[redacted]	[redacted]	[redacted]

*PK samples may also be used to identify and quantify potential metabolites of D-1553.

[redacted]

Confidential

Table 1-6 Plasma Pharmacokinetic Blood Sampling and ECG for Phase 2

At selected sites, up to a total of 30 phase 2 subjects will also be consented to have intensive PK and ECG data collected:

	**Treatment Cycle 1: Day 1; Treatment Cycle 2: Day 1; Treatment Cycle 3: Day 1**		
	**Pre-dose**	**1 hour post-dose**	**6 hours post-dose**
	**- 1 to 0 hours**	**± 10 minutes**	**± 0.5 hours**
**D-1553***	X	X	X
**ECG**	X	X	X

*PK samples will also be used to identify and quantify potential metabolites of D-1553.

Sparse PK sampling will be collected in the other subjects:

	**Treatment Cycle 1: Day 1; Treatment Cycle 3: Day 1**	
	**Pre-dose**	**1 hour post-dose**
	**- 1 to 0 hours**	**± 10 minutes**
**D-1553***	X	X
	**Treatment Cycle 1: Day 1; Treatment Cycle 3: Day 1**	
	**Pre-dose**	**1 ~ 2 hours post-dose****
	**- 1 to 0 hours**	
**ECG**	**X**	**X**

*PK samples will also be used to identify and quantify potential metabolites of D-1553.

**The time for post-dose ECG is recommended to be as close as possible to the PK sampling time unless it's too difficult for clinical practice.

Confidential

2 INTRODUCTION

2.1 Background of D-1553

KRAS mutations are prevalent in lung and colon cancers, with nearly 40% occurring in subjects with lung cancer and as many as 50% occurring in subjects with CRC (1, 2). The most common tumor types to exhibit $KRAS^{G12C}$ mutations have been determined to be in lung and colon cancers (2). Overall, it is estimated that approximately 13% of lung adenocarcinoma, 3% of CRC, and 1% to 2% of numerous other solid tumors (including pancreatic, cholangial, endometrial, bladder, ovarian, and small cell lung tumors) harbor the $KRAS^{G12C}$ mutation (3, 4). Only recently the KRAS mutation is recognized as a druggable target and new chemical entities are developed to target it. D-1553 is a novel compound in this class of compounds and is at the front line of clinical development.

D-1553 is a novel, targeted $KRAS^{G12C}$ inhibitor that is being developed as a potential oral agent for advanced or metastatic solid tumors with $KRAS^{G12C}$ mutation. A detailed description of the chemistry, pharmacology, toxicology and safety of D-1553 is provided in the Investigator's Brochure (IB). The term "study intervention" throughout the protocol refers to D-1553 or its combination with other drugs.

[redacted]

[redacted]

[redacted]

[redacted]

[redacted]

[redacted]

2.3 Background of Cetuximab

Cetuximab is a recombinant, human/mouse chimeric monoclonal antibody that binds specifically to the extracellular domain of human EGFR on both normal and tumor cells, and inhibits receptor activation by competing with epidermal growth factor and other ligands. *In vitro* and *in vivo* assays have shown that binding of cetuximab to EGFR blocks its dimerization and phosphorylation and its consequent activation, resulting in inhibition of cell growth, induction of apoptosis, and decreased matrix metalloproteinase and vascular endothelial factor production. Cetuximab exhibits clinical activity as monotherapy or in combination with chemotherapy and/or radiation in head and neck cancer and mCRC (25, 26).

Cetuximab is approved in several countries for the treatment of patients with CRC or head and neck squamous cell carcinomas. Cetuximab is approved for the treatment of EGFR-expressing, RAS^{wt} mCRC in combination with chemotherapeutic agents or as a single agent, in previously untreated patients and in patients who have failed irinotecan- and oxaliplatin-based regimens or who are intolerant to irinotecan-based regimens (see the locally applicable cetuximab label).

<u>Clinical Experience</u>

Clinical Pharmacokinetics

Following the recommended dose regimen (400 mg/m^2 initial dose; 250 mg/m^2 weekly dose), concentrations of cetuximab reach steady-state levels by the third weekly infusion. Although the US label for Erbitux® indicates that US-licensed cetuximab (Erbitux) provides approximately 22% greater exposure relative to cetuximab which is approved in the European Union (EU), the dosing and administration guidelines are consistent between the regions.

Clinical Efficacy

In patients with mCRC whose disease had progressed during or within 3 months after treatment with an irinotecan-based regimen, treatment with single-agent cetuximab resulted in an objective response rate of 11% and a median time to progression of 1.5 months (27). In patients with EGFR-expressing mCRC treated with cetuximab in combination with irinotecan in the second-line setting, the median PFS was 4.0 months (95% confidence interval [CI], 3.2-4.1 months) and OS was 10.7 months (95% CI, 9.6 -11.3 months) (28).

Clinical Safety

Across all studies, cetuximab was discontinued in 3% to 10% of patients because of AEs. The main undesirable effects of cetuximab are as follows:

- Skin reaction occurring in 76% to 88% of patients, with severe acneiform rash occurring in 1% to 17% of patients;

- Severe (Grade 3-4 by NCI CTCAE) hypomagnesemia in 6% to 17% of patients;
- Infusion reactions, with mild to moderate symptoms in 15% to 21% of patients, and severe in 1% to 5% of patients;
- Cardiopulmonary arrest and/or sudden death in 2% of patients receiving cetuximab in combination with radiation therapy;
- A case of tumor lysis syndrome reported with single-agent cetuximab within 24 hours of administration of the first dose (29).

More details are provided in the locally approved cetuximab label.

2.4 Pharmacology

The primary pharmacology of D-1553 was evaluated in a number of *in vitro* and *in vivo* studies.

In vitro studies were conducted to evaluate the binding specificity of D-1553 on $KRAS^{G12C}$ protein, the anti-proliferative effect of D-1553 on $KRAS^{G12C}$ cancer cell lines. These *in vitro* studies demonstrated that D-1553 functions as an $KRAS^{G12C}$ inhibitor, selectively inhibits the growth of $KRAS^{G12C}$ cancer cells.

In vivo studies were conducted to evaluate the therapeutic efficacy of D-1553 in NSCLC, CRC and other tumor xenograft models in mice. These results demonstrated a significant antitumor effect of D-1553 alone or in combination with chemotherapy or with other targeted therapy such as MEK inhibitors, SHP2 inhibitors in these xenograft tumor model. The combination resulted in greater antitumor activity when compared with each agent alone.

[redacted]

2.5 Pharmacokinetics and ADME

Various experiments were performed to evaluate PK profiles of D-1553, including absorption studies in both beagle dogs and Sprague Dawley rats, distribution and excretion studies in Sprague Dawley rats, and metabolism studies in the hepatocytes of different species.

PK and absorption, distribution, metabolism and excretion (ADME) of D-1553 have been thoroughly characterized. [redacted] No sex-related differences in PK parameters were seen in rats, and no accumulation of D-1553 was observed in rats or dogs with dosing up to 7 days.

D-1553 was distributed to all tissues within 0.5 hours [redacted]

[redacted]

Cytochrome 450 (CYP) phenotyping studies using human liver microsomes identified CYP3A as the major metabolic enzyme [redacted]

No induction of CYP1A2 or 3A4 enzyme activity or gene expression was observed in hepatocytes. [redacted]

D-1553 weakly inhibited CYP3A4 but had minimal/negligible effects on CYP1A2, CYP2B6, CYP2C8, CYP2C9, CYP2C19 and CYP2D6.

[redacted]

2.6 Toxicology

Adequate nonclinical studies have been conducted to characterize the toxicity profile of D-1553. For the toxicology studies, rat was used as the rodent species and the dog was used as the non-rodent species based on similarity to human metabolites from *in vitro* metabolite profiling study. Oral administration was chosen in order to comply with the intended human route of administration. All definitive toxicology studies were conducted in compliance with Good Laboratory Practice (GLP) regulations.

In a pivotal GLP-compliant 4-week repeat dose toxicity study male and female rats tolerated twice daily oral administration of D-1553 at total doses of 55, 110, and 165 mg/kg/day for up to 28 days with no effects on survival, serum chemistry, coagulation, urinalysis, gross observations, or histopathology. [redacted]

In the pivotal GLP-compliant 4-week toxicity study in dogs twice daily oral administration of D-1553 to beagle dogs at 20, 50, and 150 mg/kg/day for 28 days followed by a 28-day recovery did not result in mortality directly attributable to the test article, or adverse findings on clinical pathology, ophthalmology, and ECG. [redacted]

[redacted]

GLP-compliant genotoxicity assessment revealed that D-1553 did not exhibit mutagenic potential in the bacterial reverse mutation (Ames) assay, [redacted] was negative for the induction of micronuclei *in vivo* in a rat peripheral blood micronucleus assay.

2.7 Study Rationale

Mutations in KRAS oncogene have been shown to play an important part in pathogenesis of cancer. $KRAS^{G12C}$ mutation, an activating mutation in KRAS gene, is believed to be driving the abnormal growth of cancer cells in multiple tumor types, such as NSCLC and CRC. Several solid tumor types including NSCLC and CRC exhibit high prevalence of $KRAS^{G12C}$ mutation.

D-1553 is an orally bioavailable, small molecule inhibitor targeting at $KRAS^{G12C}$ mutation. This is a first -in -human (FIH) study to determine the fundamental elements of D-1553 safety, tolerability, PK and efficacy. Data from this study will assist in establishing safety and efficacy parameters for subsequent studies with D-1553.

2.8 Rationale for Selection of the Starting Dose of D-1553

The starting dose for D-1553 in the FIH trial in cancer subjects has been determined to be a fixed dose of 150 mg administered orally on an empty stomach (no food and drink, except water, for at least 0.5 hours before and after dose) once -daily, based on the information derived from nonclinical safety pharmacology and toxicology studies. [redacted]

Per the ICH guideline S9 Nonclinical Evaluation for Anticancer Pharmaceuticals (2010), the highest clinical starting dose for an anticancer small molecule should be equivalent or lower than 1/10 of STD_{10} in rodents or 1/6 of HNSTD in non-rodents. [redacted] Therefore, the proposed human starting dose of 150 mg/day (2.5 mg/kg/day or 92.5 mg/m^2/day) is reasonably safe and acceptable based on the available nonclinical study results.

[redacted]

	[redacted]	[redacted]	[redacted]		[redacted]
			[redacted]	[redacted]	
[redacted]	[redacted]	[redacted]	[redacted]	[redacted]	[redacted]
	[redacted]	[redacted]	[redacted]	[redacted]	[redacted]

[redacted]

2.9 Dose Escalation

Based on safety pharmacology and GLP Toxicity studies, the dose is escalated till the highest dose of 1600 mg/day. Even at 1600 mg/day, there's still a safety window [redacted] versus STD_{10}/HNSTD in 4-week GLP toxicity study in rat and dog, respectively. During the dose escalation, the SRC will review safety data from each dose cohort and determine if it is safe to go to the next higher dose level, thus ensuring subjects' safety. The SRC may decide to lower the increment for the next dose level or add additional cohort at dose escalation meeting, based on analysis of safety and PK.

2.10 Rationale for Combination Use of [redacted] Targeted Therapy

[redacted]

$KRAS^{G12C}$ mutations are found in up to 20% of mCRC cases with lower rates of prevalence in patients in later lines of therapy. Patients with $KRAS^{G12C}$ mCRC have a poor prognosis with shorter PFS and OS compared with patients with wild-type tumors (30). There are no specific therapies targeting $KRAS^{G12C}$ mCRC, and these patients are treated with combination chemotherapy without anti-EGFR agents.

Nonclinical experiments have provided compelling evidence that combining a $KRAS^{G12C}$ inhibitor with an EGFR inhibitor results in greater anti-tumor effects than either agent alone in $KRAS^{G12C}$ CRC cells (31). Consistent with these observations, the combination of the $KRAS^{G12C}$ inhibitor, D-1553 and cetuximab resulted in a strong synergistic antitumor activity.

As of 8 June 2022, a preliminary efficacy was observed in the current study from $KRAS^{G12C}$ mutated mCRC patients who received D-1553 600 mg BID monotherapy, with an ORR of 36.6% (4/11) which was higher than or similar to the ORR of other KRAS G12C inhibitors (e.g., sotorasib and adagrasib) monotherapy in mCRC patients.

Adagrasib has been used in combination with cetuximab in a clinical trial where32 pts with CRC (3 median prior lines of therapy) were treated with adagrasib + cetuximab. TRAEs of any grade occurred in 100% and grade 3/4 events in 16% of patients, with no grade 5 events. Among the 28 patients evaluable for clinical activity, the response rate was 43% (12/28, including 2 unconfirmed PRs who remain on study) and DCR was 100%. Adagrasib was well tolerated as combined with cetuximab and demonstrated promising clinical activity in heavily pretreated patients with KRAS^{G12C} mutant CRC (32).

Based on early clinical data suggesting clinical activity of D-1553 monotherapy and adagrasib/cetuximab combination therapy in patients with KRAS^{G12C} mCRC, the current study is designed to evaluate D-1553 in combination with [redacted] targeted therapy (e.g., cetuximab) in patients with KRAS^{G12C} mCRC. Since D-1553 antitumor activity is expected in Phase 2 Arm B and Arm F, the design of these two arms is converted into a direct expansion cohort design from the previous Simon 2 stage design.

[redacted]

[redacted]

[redacted]

[redacted]

[redacted] [redacted]

[redacted]

[redacted]

[redacted]

[redacted]

[redacted]

2.13 Benefit/Risk Assessment

D-1553 is a novel oral inhibitor targeting $KRAS^{G12C}$ mutation in KRAS oncogene. $KRAS^{G12C}$ mutation is believed to drive the tumor cell growth in multiple tumor types such as NSCLC and CRC that harbor this specific mutation. This study is the FIH clinical trial in subjects with advanced or metastatic cancer. There is no human data of D-1553 before this study. In preclinical studies, D-1553 has shown inhibitory effect of cancer cell lines bearing $KRAS^{G12C}$ mutation and antitumor activity in animal models bearing $KRAS^{G12C}$ mutated tumors. However, the benefit of D-1553 in cancer subject population is not yet established.

As this is the first clinical trial of D-1553 in human, the risk of D-1553 in cancer subjects is unknown. During the Phase 1 portion of the study, D-1553 will start at low dose level and escalate to higher dose levels after the safety and tolerability of each dose level cohorts are reviewed. Specific dose escalation rules are described in the protocol based on the number of DLTs observed in each cohort to ensure that once an unsafe dose level is determined, no more subjects will be given D-1553 at that dose level or higher. All subjects will be monitored to detect adverse events (AEs) during the study and followed appropriately to ensure resolution of AEs.

The study population is subjects with $KRAS^{G12C}$ mutated advanced or metastatic cancer. In this subject population the standard treatment is very limited and subjects frequently become resistant or refractory to standard treatments. The study will have strict inclusion and exclusion criteria to enroll subjects with severe or life-threatening advanced or metastatic cancers with $KRAS^{G12C}$

mutation for whom there is no or very limited standard therapy. In this subject population, the benefit/risk ratio of an investigational targeted agent is favored and this research study is warranted.

3 STUDY OBJECTIVES AND ENDPOINTS

3.1 Phase 1 Objective(s)

3.1.1 Primary Objectives

- To assess the safety and tolerability of D-1553 single agent and in combination with [redacted] in subjects with advanced or metastatic solid tumors with $KRAS^{G12C}$ mutation
- To determine the DLT, MTD and RP2D of D-1553 single agent and in combination with [redacted] in subjects with advanced or metastatic solid tumors with $KRAS^{G12C}$ mutation

3.1.2 Secondary Objectives

- To evaluate the PK of D-1553 alone and in combination with [redacted] in subjects with advanced or metastatic solid tumors with $KRAS^{G12C}$ mutation
- To preliminarily evaluate the antitumor activity of D-1553 alone and in combination with [redacted] in subjects with advanced or metastatic solid tumors with $KRAS^{G12C}$ mutation

3.2 Phase 1 Endpoints

3.2.1 Primary Endpoints

- Type, incidence, severity (graded by NCI CTCAE, v5.0), attribution and timing of AEs
- Incidence of DLTs

3.2.2 Secondary Endpoints

- PK parameters (AUC_{0-t}, AUC_{inf}, MRT, C_{max}, t_{max}, C_{min}, $t_{½}$, Vd_{ss}/F and CL/F) of D-1553 [redacted]
- ORR (CR + PR), DCR (CR + PR + SD), PFS and DOR, evaluated by RECIST, v1.1

3.3 Phase 2 Objective(s)

3.3.1 Primary Objective:

- To assess the antitumor effect of D-1553 single agent and in combination with [redacted] targeted therapy in subjects with advanced or metastatic solid tumors with $KRAS^{G12C}$ mutation.

3.3.2 Secondary Objectives:

- To assess the safety and tolerability of D-1553 in subjects with advanced or metastatic solid tumors with $KRAS^{G12C}$ mutation
- To evaluate the PK of D-1553

3.4 Phase 2 Endpoints

3.4.1 Primary Endpoint

- ORR (CR + PR)

3.4.2 Secondary Endpoints

- DCR (CR + PR + SD), PFS, DOR, evaluated by RECIST, v1.1 and OS
- Type, incidence, severity (graded by NCI CTCAE, v5.0), attribution and timing of AEs
- Based on PK data obtained in this study as well as PK data obtained from other studies, a population PK analysis will be performed to characterize pharmacokinetic parameters (Clearance (CL), Volume of distribution (V)) and evaluate the effect of extrinsic and intrinsic factors to support proposed dosing regimen

4 INVESTIGATIONAL PLAN

4.1 Overall Study Design

This is a phase 1/2, open label study of D-1553 single agent and in combination with [redacted] targeted therapy to assess the safety and tolerability, identify the MTD and RP2D, evaluate the PK properties and antitumor activities in subjects with advanced or metastatic solid tumor with KRAS^{G12C} mutation. The study is divided into Phase 1 and Phase 2 portions. Phase 1 will consist of 2 parts: Phase 1a (dose escalation) and Phase 1b (dose combination). Subjects will take D-1553 orally on an empty stomach every day in a 21 or 28day treatment cycle. The study treatment will be administered until disease progression, withdrawal of consent, unacceptable toxicity or discontinuation from the study for other reasons.

4.1.1 Phase 1a (Dose Escalation)

Phase 1a will start to evaluate 4 sequential cohorts administered with once -daily oral doses of D-1553 (see Table 4-1): Cohort 1 (150 mg once -daily), Cohort 2 (300 mg once -daily), Cohort 3 (600 mg once -daily) and Cohort 4 (1200 mg once daily). An additional 3 sequential cohorts administered with twice- daily oral doses of D-1553 will also be evaluated: Cohort 5 (400 mg twice- daily; total 800 mg daily), Cohort 6 (600 mg twice- daily; total 1200 mg daily) and Cohort 7 (800 mg twice- daily; total 1600 mg daily). After the safety of Cohort 3 has been determined, the dose escalation of the twice daily administered cohorts (Cohorts 5 to 7) will be conducted in parallel to the once- daily administered Cohort 4.

Table 4-1 Dose Escalation Cohorts For Phase 1a

**Cohorts (once daily)**	**Cohort 1**	**Cohort 2**	**Cohort 3**	**Cohort 4**		
**Dose**	150 mg once daily	300 mg once daily	600 mg once daily	1200 mg once daily		
**Cohorts (twice daily)**				**Cohort 5**	**Cohort 6**	**Cohort 7**
**Dose**				400 mg twice daily	600 mg twice daily	800 mg twice daily

The starting dose of 150 mg once daily in human was selected based on the information derived from nonclinical safety pharmacology and toxicology studies. This dose has been intentionally chosen as a conservative starting point to maximize safety margins according to the standard DeGeorge method[redacted]

Accelerated titration (single subject per cohort) will be used for Cohorts 1 and 2 until one Grade 2 or higher at least possibly TRAE occurs during the DLT observation period (treatment cycle 1), after which another 2 subjects will be enrolled in the same cohort and the dose escalation will be converted to follow conventional 3+3 design. If no Grade 2 or higher TRAE occurs in Cohorts 1

and 2, Cohort 3 will start to follow the conventional 3+3 design. Cohorts 5 to 7 will always follow the conventional 3+3 dose escalation design.

The conventional 3+3 dose escalation will be conducted as follows:

Three subjects will be enrolled and treated in the current cohort and observed for DLT during the DLT observation period (treatment cycle 1).

- If there is no DLT, the next higher dose cohort can start.
- If there is one DLT, another 3 subjects will be enrolled and treated.
  - If there is no more than one DLT in 6 subjects, the next higher dose cohort can start.
  - If there are more than one DLTs in 6 subjects, the MTD has been exceed and the next lower dose will be the MTD or an intermediate dose may be evaluated.
- If there are two or more DLTs, the MTD has been exceeded and the next lower dose will be the MTD or an intermediate dose may be evaluated.

This 3+3 dose escalation scheme will be repeated until the MTD is determined or the highest planned dose is reached.

Each subject will receive daily oral doses of D-1553 (once daily for Cohorts 1 to 4 and twice daily for Cohorts 5 to 7) from Day 1 to Day 21 on an empty stomach [redacted] Each 21-day period will be considered as 1 treatment cycle. Subjects may continue daily D-1553 until disease progression, unacceptable treatment related toxicity, withdrawal of consent or discontinuation for any reason, whichever occurs first. Safety will be assessed throughout treatment cycles and during followup periods. Dose limiting toxicity will be evaluated during the 1st treatment cycle. Pharmacokinetic parameters ($AUC_{0\text{-}t}$, AUC_{inf}, MRT, C_{max}, t_{max}, C_{min}, $t_{½}$, V_{dss}/F and CL/F) will be evaluated during the 1st and 3rd treatment cycles. PK timepoints of subsequent cohorts may be adjusted based on PK results from Cohort 1 and/or Cohort 2. Tumor assessments will be performed at screening and every 2 treatment cycles for the first 8 treatment cycles and every 3 treatment cycles thereafter until disease progression.

An SRC, [redacted] will regularly assess the safety of D-1553 and its combination therapy throughout the study. After completion of the 1st treatment cycle in each cohort of the conventional 3+3 dose escalation scheme, the SRC will review all available data to recommend whether or not to proceed to the next cohort. The SRC may recommend to lower the increment for the next dose level or add additional cohorts at the dose escalation meeting, based on the safety and PK results. Intermediate dose escalation levels (between Cohorts 1 and 4; Cohorts 5 and 7) may be evaluated during the study, as shown in Table 4-2.

Table 4-2 Intermediate Dose Escalation Cohorts for Phase 1a

**Cohorts (once daily)**	**Cohort 1**	**Cohort 2**	**Cohort 3**	**Cohort 4**		
**Dose**	150 mg once daily	300 mg once daily	600 mg once daily	1200 mg once daily		
**Intermediate Dose**	200 mg once daily	450 mg once daily	900 mg once daily			
**Cohorts (twice daily)**				**Cohort 5**	**Cohort 6**	**Cohort 7**
**Dose**				400 mg twice daily	600 mg twice daily	800 mg twice daily
**Intermediate Dose**			300 mg twice daily	500 mg twice daily	700 mg twice daily	

At the highest dose level or MTD, the cohort may be expanded to about 10 subjects for further evaluation of safety, PK and antitumor activity. When a higher dose is determined to be safe, the previous lower dose cohort may be backfilled if further evaluation deems necessary. Subjects who completed the DLT evaluation period can receive a higher dose in subsequent cycles provided that they have tolerated the initial dose and the increased dose has been studied and proven safe. The RP2D (at or below MTD) of D-1553 as a single agent and the way it will be administered (once daily or twice daily) will be chosen based on SRC review of the safety and PK data at the completion of Phase 1a, for further evaluation in Phases 2 Arms A and B. This dose should be safe and has shown sufficient PK exposure and/or preliminary antitumor activity in Phase 1a result.

4.1.2 Phase 1b (Dose Combination)

Phase 1b will determine the MTD of D-1553 in combination with [redacted] in subjects with advanced or metastatic [redacted] and other solid tumors. There are 4 groups in Phase 1b for different tumor types and combinations:

- [redacted]

[redacted]

The enrollment to all groups ([redacted]) will occur at the same time and each group will be evaluated independently. From Phase 1a results, if the RP2D of D-1553 single agent is the same as MTD, each group will have 2 dose level cohorts of D-1553 to assess the safety and tolerability of D-1553 in combination with [redacted] in a conventional 3+3 design: Cohort 1 (1 dose level below RP2D of D-1553 single agent) and Cohort 2 (RP2D of D-1553 single agent). Three subjects will be enrolled in Cohort 1 first. If there is no DLT, Cohort 2 will be started. If there is 1 DLT, another 3 subjects will be enrolled in Cohort 1.

If there is no DLT in the additional 3 subjects, Cohort 2 will be started. If at any time there are more than 1 DLTs in a cohort of 3-6 subjects, the MTD has been exceeded and the next lower dose level is declared to be the MTD and safe. A dose level below Cohort 1 dose level may be added as Cohort -1 for 3+3 scheme evaluation, if Cohort 1 level is determined to be unsafe.

From Phase 1a results, if the RP2D of D-1553 single agent is lower than MTD or the MTD is not reached at the highest dose, each group in Phase 1b will start with Cohort 2 (RP2D of D-1553 single agent) in a cohort of 3 subjects to evaluate the safety and PK of combination regimens.

The SRC will review the safety and PK data (if available) of each dose cohort, after completion of the 1st treatment cycle to determine whether or not to proceed to further expansion or de-escalate to the next level. The SRC may recommend to evaluate an intermediate dose level or up to two levels lower than RP2D of D-1553 single agent, based on analysis of the safety data. Once the MTD is determined, or the RP2D of D 1553 single agent is determined to be safe with the combination regimen, that dose level group ([redacted]) may proceed to Phase 2 in Arms [redacted] F, respectively. [redacted]

Each subject will receive an oral dose of D-1553 (once-daily or twice-daily) at the assigned dose, combined with [redacted] cetuximab in a 21-day cycle. Subjects may continue study treatment until disease progression, unacceptable treatment -related toxicity, withdrawal of consent or discontinuation from study for other reasons, whichever occurs first. [redacted] Safety will be assessed throughout treatment cycles and follow-up periods. Pharmacokinetic parameters (AUC, -C_{max}, t_{max}, C_{min}, $t_{½}$, V_{dss}/F and CL/F) will be evaluated during the 1st and 3rd treatment cycles. Tumor assessments will be performed at screening every 2 treatment cycles for the first 8 treatment cycles and every 3 treatment cycles thereafter until disease progression.

Once a PR or CR is observed, a confirmatory tumor assessment is recommended to be performed with at least 4 weeks' interval per RECIST 1.1.

[redacted]

[redacted]

[redacted]

[redacted]

[redacted]

[redacted]

[redacted]

[redacted]

[redacted]

[redacted]

4.1.3 Phase 2

The Phase 2 portion is a 6-arm, parallel, open-label study to evaluate the efficacy of D-1553 single agent and combination treatments in subjects with advanced or metastatic solid tumors with $KRAS^{G12C}$. [redacted] The futility criteria are based on assumptions for the ORR for the SOC and the expected effect based on the addition of D-1553 to the SOC.

Table 4-3 Simon 2-stage design futility criteria for [redacted]

The alternative vs null objective response rate rates (P1 vs P0)	Futility stage 1	Futility stage 2	Applies to
[redacted]	[redacted]	[redacted]	[redacted]
[redacted]	[redacted]	[redacted]	[redacted]
[redacted]	[redacted]	[redacted]	[redacted]

Arms [redacted] B are for single agent D-1553 treatment and Arms [redacted] F are for combination treatments, as follows:

- [redacted]
- Arm B: solid tumors other than NSCLC treated with D-1553 single agent at RP2D; [redacted]
- [redacted]

[redacted]

- Arm F: CRC, D-1553 in combination with cetuximab

Upon completion of Phase 1a (single agent dose escalation), Phase 2 Arms [redacted] B may be started, while Phase 2 Arms [redacted] F may be started once Phase 1b (dose combination) is completed. The sequence of enrollment to each group or arm will be communicated to investigational sites by study team. [redacted] Each arm will enroll approximately 30 subjects for evaluation of safety and efficacy in subjects with $KRAS^{G12C}$ bearing solid tumors. If 1 or more arms demonstrate significant antitumor activity that

warrants further evaluation, the protocol might be amended to allow further expansion of the respective arm(s).

Approximately 70 subjects will be enrolled in Phase 2 Arm B.

Approximately 40 subjects will be enrolled in Phase 2 Arm F.

Each subject will receive an oral dose of D-1553 at RP2D. Each 21- or 28-day period will be considered as 1 treatment cycle. Subjects may continue with daily administration of D-1553 until disease progression, unacceptable treatment-related toxicity, withdrawal of consent or discontinuation from the study for other reasons, whichever occurs first. In the combination arms, D-1553 may be continued at the same dose level after the combination regimen is completed, if the investigator considers it is in the best interest of the subject, after consulting with the sponsor's medical representative. [redacted] Safety will be assessed throughout treatment cycles and during follow-up periods. PK samples will be collected in the 1st and 3rd treatment cycles. Tumor assessments will be performed at screening and after every 2 treatment cycles for the first 8 treatment cycles and every 3 treatment cycles thereafter until disease progression.

Once a PR or CR is observed, a confirmatory tumor assessment is recommended to be performed with at least 4 weeks' interval per RECIST 1.1.

Subjects who do not have disease progression on study will be followed for tumor assessment, approximately every 12 weeks (±2 weeks), until disease progression, death, lost to follow-up, start of another anticancer therapy, or other reasons that a follow-up is not possible. Subjects will be followed for survival status monthly (± 2 weeks), until death, lost to follow-up or other reasons that a follow-up is not possible. The follow-up can be done via telephone call.

[redacted]

[redacted]

[redacted]

[redacted]

[redacted]

[redacted]

[redacted]

[redacted]

Targeted Therapy in Arm F

In combination with D-1553 (once daily or twice daily), subjects will receive an intravenous dose of cetuximab (400 mg/m^2 initial dose, 120-minute IV infusion on Cycle 1 Day 1), then 250 mg/m^2 (60-minute IV infusion) once weekly. Thereafter, each 21-day cycle will be considered as 1 treatment cycle. D-1553 and cetuximab may continue until disease progression, unacceptable treatment related toxicity, withdrawal of consent or discontinuation from study for other reasons, whichever occurs first.

4.1.4 Dose Escalation and Stopping Rules

This study will evaluate the safety, tolerability, PK and efficacy of D-1553 administered as a single agent and in combination with [redacted] targeted therapy in subjects with advanced or metastatic solid tumors with KRAS^{G12C} mutation. In the Phase 1 part of the study, the design requires careful multiple dose level evaluations and dose escalation based on pre-set safety evaluation criteria. After the MTD or highest planned dose of D-1553 as a single agent or in combination regimen is reached, the study will go into Phase 2 evaluation to further assess the

safety, PK and efficacy of D-1553 and its combinations in subjects with advanced or metastatic solid tumors with KRAS^{G12C} mutation. The Phase 1 design is standard and is considered to meet the objectives of the study. The Phase 1 and Phase 2 are combined into 1 study to ensure a smooth and timely transition from Phase 1 to Phase 2 to establish the proof-of-concept of treatment effect of the study treatment.

The safety assessments for the study are accepted measures for ensuring the safety of subjects during a clinical study. The PK sampling schedule is considered appropriate given the information available from preclinical studies. The rationale for the clinical study is presented in Section 2.7.

4.1.5 Definition of Maximum Tolerated Dose/Recommended Phase 2 Dose

The MTD is the highest dose level of D-1553 that doesn't cause unacceptable side effects. During dose escalation in Phase 1, if 2 out of 6 subjects experience DLTs (side effects that prevent a dose increase) at a given dose level during the first cycle of the therapy, then the MTD has been exceeded and dose escalation will be stopped. The MTD will be determined as the next lower dose level. The RP2D will be at or below MTD.

4.2 Dose-limiting Toxicity Criteria

[redacted]

[redacted]

[redacted]

[redacted]

[redacted]

[redacted]

[redacted]

[redacted]

[redacted]

[redacted]

[redacted]

[redacted]

[redacted]

[redacted]

[redacted]

[redacted]

[redacted]

[redacted]

[redacted]

[redacted]

[redacted]

[redacted]

[redacted]

[redacted]

[redacted]

- [redacted]
- [redacted]
- [redacted]
- [redacted]

4.2.2 Dose-limiting toxicity evaluation

The DLT evaluation period is the first cycle of treatment and starts from treatment cycle 1/Day 1. If a subject is not evaluable for DLT due to reasons other than treatment-related toxicities in treatment cycle 1, then an additional subject will be enrolled in the cohort for replacement.

4.3 Study Termination Criteria

The estimated duration of the study is contingent on the number of dose levels evaluated in the dose escalation. The study will be discontinued in the event of any new findings that indicate a relevant deterioration of the risk-benefit relationship that would render continuation of the study unjustifiable, or for administrative reasons by the sponsor.

5 STUDY POPULATION

Prospective approval of protocol deviations to recruitment and enrollment criteria, also known as protocol waivers or exemptions, is not permitted.

The study population will consist of subjects with advanced or metastatic solid tumors with KRAS^{G12C} mutation. Subjects must be able to provide written consent and meet all the inclusion criteria and none of the exclusion criteria.

5.1 Inclusion Criteria

Subjects are eligible to be included in the study only if all of the following criteria apply:

1. Subject must be fully informed about their illness and the investigational nature of the study protocol (including foreseeable risks and possible side effects) and must have signed and dated an Institutional Review Board (IRB) /Independent Ethics Committee (IEC) approved Informed Consent Form (ICF) that is in accordance with regulatory and ethics guidelines and it must be obtained before the performance of any protocol related procedures or tests.
2. Subject is male or female of at least 18 years of age at the time of signing informed consent.
3. Subject must be willing and able to comply with all scheduled visits, treatment, laboratory tests, be able to take oral medication and accept other requirements of the study.
4. Subject with histologically or cytologically proven, locally advanced, unresectable and/or metastatic solid tumor.
5. Subject has KRAS^{G12C} mutation in tumor tissue, blood, pleural effusion or other samples containing cancer cells or DNA. [redacted]
6. Phase 2 subjects must be able to provide a sufficient amount of representative tumor specimen (archival sample collected within 5 years) and ctDNA samples for central laboratory testing of KRAS mutation status. Subject with KRAS^{G12C} mutation in tumor or ctDNA samples local testing is acceptable for eligibility; all patients in Phase 2 study will have confirmation of KRAS^{G12C} mutation by Sponsor-designated central laboratory [redacted]
7. Subject has tumor type requirement as follows:
    a. Phase 1a: advanced or metastatic solid tumors for which no standard treatment is available or the subject is refractory to or intolerant of existing standard treatment;

    [redacted]

[redacted]

[redacted] [redacted]

[redacted] [redacted]

[redacted] [redacted]

f. Phase 2 Arm B: advanced or metastatic solid tumors other than NSCLC for which no standard treatment is available or the subject is refractory to or intolerant of existing standard treatment;

g. [redacted] Phase 2 Arm F: advanced or metastatic CRC that have progression of disease after at least 1 prior regimens:

- Disease relapse during treatment or within 6 months following adjuvant therapy will be considered advanced or metastatic disease;
- Patients who have received 2 prior regimens (i.e. those entering the study in the 3rd line setting), must have received oxaliplatin unless it was contraindicated due to underlying conditions.

8. Subject has measurable disease according to RECIST, v1.1. For Phase 1 portion, subject with evaluable disease according to RECIST, v1.1 is also acceptable.

9. Subject in Phase 1 has an Eastern Cooperative Oncology Group (ECOG) performance status of Grade ≤ 2, [redacted]. Subject in Phase 2 has an ECOG performance status of Grade 0 or 1.

10. Subject has adequate hematologic function, defined as:

- Platelet count ≥ 100 × 10^9/L;
- Hemoglobin level ≥ 8.0 g/dL. [redacted] Participants can be on stable dose of erythropoietin (≥ approximately 3 months);

- Absolute neutrophil count ≥ 1.5 × 10^9/L.

[redacted]

11. Subject has adequate renal function, defined as creatinine clearance ≥ 60 mL/min, as calculated using the modified Cockroft Gault equation or other institutional standard formula, or creatinine ≤ 1.5 × upper limit of normal (ULN).

12. Subject has adequate liver function, defined as:
    - Aspartate aminotransferase (AST) levels ≤ 2.5 × ULN (if liver metastases are present, ≤ 5 × ULN);
    - Alanine aminotransferase (ALT) levels ≤ 2.5 × ULN (if liver metastases are present, ≤ 5 × ULN);
    - Total bilirubin ≤ 1.5 × ULN (not applicable to subjects with Gilbert's syndrome).
    - [redacted]
        - [redacted]
        - [redacted]

13. Subject has adequate coagulation function, defined as prothrombin time and activated partial thromboplastin time ≤ 1.5 × ULN, and International normalized ratio (INR) ≤ 1.5. For subjects on anticoagulation therapy, the INR should be < 3.0 or within the target range of anticoagulation therapy if applicable (INR should be used instead of prothrombin time for subjects on anticoagulation therapy).

14. [redacted]

15. Female of childbearing potential must agree to abstain or use effective contraception methods from the time of signing ICF and for the duration of study participation through 6 months after the last dose of study drug. Acceptable contraceptive methods include: oral, injected or implanted hormonal methods of contraception; intrauterine device or intrauterine system; occlusive cap (diaphragm or cervical/vault caps) with spermicide together with male condom. If needed, investigators can select from the above contraception methods to meet the country or institutional standards of an effective contraception method.

- A female subject of childbearing potential is any woman, regardless of sexual orientation, who meets the following criteria: a) not surgically sterile with procedures like tubal ligation, hysterectomy, bilateral salpingectomy or bilateral oophorectomy; or b) not been naturally postmenopausal for at least 12 consecutive months (i.e., has had menses at any time in the preceding 12 consecutive months).

16. Male subjects must agree to abstain, be surgically sterilized, or agree to use an effective contraceptive method from the time of signing of ICF and for the duration of study participation through 6 months after the last dose of study drug. Effective contraceptive methods include: a) simultaneous use of condom, and for the female partner, hormonal contraceptives or intrauterine contraceptive device (used since at least 4 weeks prior to dosing); b) simultaneous use of condom, and for the female partner, diaphragm or cervical/vault caps with spermicide. If needed, investigators can select from the above contraception methods to meet the country or institutional standards of an effective contraception method.

5.2 Exclusion Criteria

Subjects are excluded from the study if any of the following criteria apply:

1. Subject has prior anticancer or investigational drug treatment within the following windows:
   a. Prior treatment with an inhibitor specific to KRAS^{G12C} mutation, such as AMG 510, MRTX849, LY3499446, JDQ443 and GDC-6036 (for subjects received previous treatment of KRAS^{G12C} inhibitors, the subjects are acceptable to participate in [redacted] Prior treatment with EGFR inhibitors (for [redacted] Phase 2 Arm F subjects only).
   b. Any anticancer therapy (including chemotherapy, targeted therapy, immune therapy, etc.) or any other investigational drug therapy less than 14 days or 3 half-lives (whichever is shorter) prior to first dose of study intervention.
2. Subject with unstable or progressive central nervous system (CNS) metastases and/or carcinomatous meningitis. Subjects with history of brain metastases are allowed, if they are clinically stable. [redacted]
3. Subjects with clinically significant cardiovascular disease, including:

- Subject with acute myocardial infarction, severe/unstable angina; or with cardiac insufficiency of New York Heart Association Functional Classification Grade 2 or above;
- Subject has corrected QT interval using Fridericia's formula (QTcF) prolongation at rest, where the mean QTc interval is > 470 msec based on triplicate measurements of electrocardiogram (ECG);
- History or current evidence of serious uncontrolled ventricular arrhythmias requiring drug therapy;
- Left ventricular ejection fraction (LVEF) < 50% (for [redacted] Arm F subject only);
- Congenital long QT syndrome, or any known history of torsade de pointes (TdP), or family history of unexplained sudden death;
- Clinically uncontrolled hypertension (after standard antihypertensive treatment, systolic blood pressure ≥ 150 mmHg and/or diastolic blood pressure ≥ 100 mmHg).

4. Subject has a history of (non-infectious) pneumonitis/interstitial lung disease that required steroids or has current pneumonitis/interstitial lung disease or any active systemic infection including but not limited to severe acute respiratory syndrome coronavirus 2 (SARS-CoV-2) infection.
5. For China and Taiwan region sites only [redacted]
   - Subject with human immunodeficiency virus (HIV) infection (positive HIV 1/2 antibody), hepatitis B infection (positive hepatitis B surface antigen), or hepatitis C infection (quantitative HCV RNA result greater than the lower limit of detection of the assay).

     [redacted]
6. Subject has any history or evidence of substance abuse or medical, psychological or social conditions that may, in the opinion of the investigator, interfere with participation in the study or evaluation of the study results.
7. Subject has impaired gastrointestinal (GI) function or GI diseases that may significantly alter the absorption or metabolism of oral medications.
8. Other serious illness or medical conditions at the investigator's discretion, that may influence study results, including but not limited to serious infection, diabetes, cardiovascular and cerebrovascular accident (CVA) (<6 months before study entry) and lung disease.

9. Subject has unresolved toxicities from prior anticancer therapy, defined as toxicities (other than alopecia) not yet resolved to NCI CTCAE, v5.0, Grade ≤ 1 (Grade ≤ 2 for peripheral neuropathy).

   [redacted]

10. Subject had major surgery within 4 weeks prior to study treatment administration or last dose of palliative radiation therapy within 2 weeks prior to study treatment administration.
    - Subject must have recovered adequately from the surgery and/or any complications of the surgery prior to starting study treatment.
    - Subject must have recovered from all radiation-related toxicities, not requiring corticosteroids, and with no occurrence of radiation pneumonitis. A 1-week washout is permitted for palliative radiation that lasted ≤2 weeks for non-CNS disease.
11. Subject has received radiation therapy >30 Gy to the lung within 6 months of the first dose of study treatment
12. Subject with uncontrolled pleural effusion, pericardial effusion or ascites. A subject who is clinically stable following treatment for these conditions (including therapeutic thoraco- or paracentesis, indwelling catheters) is eligible.
13. Subject is currently receiving or planning to receive medications known to be substrate of CYP3A4 with a narrow therapeutic window, strong inducer or strong inhibitor of CYP3A4, with a known risk to prolong the QT interval or strong inhibitor of P-glycoprotein within 14 days or 5 half-lives of the drug or its major active metabolite, whichever is shorter, prior to study day 1 [redacted]
14. Subject has any other known primary malignancy that is progressing or has required active treatment within the past 3 years. Note: subjects with basal cell carcinoma of the skin, squamous cell carcinoma of the skin, or carcinoma in situ (e.g., breast carcinoma, cervical cancer in situ) that have undergone potentially curative therapy are not excluded.
15. Subject is pregnant or lactating.
16. [redacted]

- [redacted]
- [redacted]
- [redacted]
- [redacted]
- [redacted]
- [redacted]
- [redacted]
- [redacted]

5.3 Subject Completion and Withdrawal

5.3.1 Subject Completion

In the dose escalation part of this study, a subject will be considered complete if he/she has a valid PK profile and has not withdrawn from the study prior to completing DLT observation period (at least one cycle). A subject will also be considered complete if he/she discontinued study treatment due to a DLT with or without a valid PK profile.

In other parts of the study, a subject is considered to have completed the study if he/she has completed study treatment as defined in the protocol, experienced disease progression, started another anticancer treatment, or been withdrawn by the investigator or sponsor for administrative reasons.

The end of the study for each subject is defined as the last visit of each subject. The end of the study is defined as the date of the last visit of the last subject in the study or last scheduled

procedure shown in the Schedule of Activities (SoA, Table 1-1 and Table 1-2 for Phase 1, Table 1-3 and Table 1-4 for Phase 2) for the last subject in the trial globally.

5.3.2 Subject Withdrawal

A subject may withdraw from the study at any time at his/her own request or may be withdrawn at any time at the discretion of the investigator for safety, behavioral or administrative reasons. The subject will be definitively discontinued from both the study intervention and from the study at that time. If the subject withdraws from the study intervention, survival follow up may still be conducted until death, lost to follow-up or other reasons that a follow-up is not possible.

If the subject withdraws consent for disclosure of future information, the sponsor may retain and continue to use any data collected before such a withdrawal of consent.

If a subject withdraws from the study, he/she may request destruction of any samples taken and not tested, and the investigator must document this in the site study records.

At the time of discontinuing from the study, if possible, an early discontinuation visit should be conducted, as shown in the SoA (Table 1-1 and Table 1-2 for Phase 1, Table 1-3 and Table 1-4 for Phase 2).

Refer to the SoA (Table 1-1 and Table 1-2 for Phase 1, Table 1-3 and Table 1-4 for Phase 2) for data to be collected at the time of study discontinuation and follow-up and for any further evaluations that need to be completed.

5.4 Subject Replacement

In this study, subjects who are enrolled but not treated and subjects who discontinue treatment before completion of cycle 1 in Phase I for reasons other than the occurrence of a DLT may be replaced. Any replacement subject will be enrolled in the same dose group.

6 STUDY TREATMENT

6.1 Allocation to Treatment

Subjects in this study will be identified by a subject number. Each subject in this study will receive a unique subject number after signing the informed consent. Subject and treatment numbers will be assigned in chronological order starting with the lowest number in phase 1 study. Once a subject number and treatment number have been assigned to a subject, it cannot be reassigned to any other subject.

Dose level assignment will be performed centrally by the sponsor or its designated representatives. The study site will receive confirmation of enrollment along with the next available date for dosing and the assigned dose level from the sponsor or its designated representatives. Sponsor or its designated representatives will notify the other sites for the enrollment of a new subject and the next possible enrollment date.

6.2 Investigational Product D-1553

6.2.1 Formulation, Appearance, Packaging and Labeling

The investigational product D-1553 will be supplied by InventisBio. The drug product is formulated as yellow, round tablets containing an equivalent of either 50 mg or 200 mg of D-1553 each [redacted]

The contents of the label will be in accordance with all applicable regulatory requirements.

6.2.2 Handling and Storage

The investigational product will be dispatched to a study center only after receipt of the required documents in accordance with applicable regulatory requirements and the sponsor's procedures.

Investigational product must be dispensed or administered according to procedures described herein. Only subjects enrolled in the study may receive investigational product, in accordance with all applicable regulatory requirements. Only authorized study center personnel may supply or administer investigational product. All investigational products must be stored in a secure area with access limited to the investigator and authorized study center personnel and under physical conditions that are consistent with investigational product-specific requirements. [redacted] Details of handling and storage information can be found in the label of the investigational product package.

6.2.3 Product Accountability

The investigator or designee will manage investigational product inventory and accountability. Logs will be provided by the sponsor or its contractors, or site use same template from standard operating procedure (SOP) with sponsor approval. The drug product distribution and log will be managed centrally by a designated contractor/vendor.

The investigator or designee will maintain accurate records of receipt of all test articles, including dates and amounts of receipt. In addition, accurate records will be kept regarding when and how much investigational product is dispensed and used by each subject in the study. At the completion of the study, to satisfy regulatory requirements regarding investigational product accountability, all investigational product will be reconciled and retained or destroyed according to applicable local, provincial and federal regulations.

The investigator or designee agrees to dispense and administer investigational product only to subjects under his/her personal supervision. The investigator will not supply investigational product to any person not authorized to receive it and will not use product for any purpose other than that permitted in the protocol. All investigational product must be kept in a secure room with a temperature controlled and access restricted to only necessary study site personnel. Used and unused investigational product and corresponding containers will be stored securely in the study centers. All investigational products will be disposed of by the sponsor or its contractors after the completion of the study.

6.2.4 Administration

In Phase 1a, each subject will receive daily oral doses of D-1553 (once -daily for Cohorts 1 to 4 and twice -daily for Cohorts 5 to 7) from Day 1 to Day 21 as one cycle on an empty stomach (no food and drink, except for water, for at least 0.5 hours before and after dosing) (Table 6-1). In Phase 1b, each subject will receive oral doses of D-1553 every day at recommended dose and schedule based on Phase 1a results in combination with other drugs. In Phase 2, each subject will receive oral doses of D-1553 every day at single agent RP2D or combination RP2D. All D-1553 doses should be taken on an empty stomach. On days when the subject is scheduled for a visit, D-1553 should be administered orally in the presence of the site personnel, especially when blood is collected for PK analysis. During the study, D-1553 should be taken at approximately the same time each day without treatment breaks until unacceptable toxicity, disease progression or withdrawal from the study. In the twice-daily schedule, D-1553 should be taken approximately 12 hours apart (±3 hours).

If a subject misses a dose, the dose will not be “made up” nor doubled at the next dose. If a subject vomits after taking a dose, the dose will not be “made up”, but the subject is to resume subsequent doses at the next day as prescribed.

Subjects will be instructed to swallow D-1553 tablets as a whole without chewing prior to swallowing. No tablet should be ingested if it is broken, cracked or otherwise not intact.

Table 6-1 Phase 1a (Dose Escalation)

Dose Levels	Dose Levels	Tablets Required
Cohort 1	150 mg once-daily	3 × 50 mg
Cohort 2	300 mg once-daily	1 × 200 mg + 2 × 50 mg
Cohort 3	600 mg once-daily	3 × 200 mg
Cohort 4	1200 mg once-daily	6 × 200 mg
Cohort 5	400 mg twice-daily	(2 × 200 mg) × 2
Cohort 6	600 mg twice-daily	(3 × 200 mg) × 2
Cohort 7	800 mg twice-daily	(4 × 200 mg) × 2

6.2.5 Discontinuation of Study Treatment

All study subjects may continue to receive daily D-1553 until unacceptable toxicity, disease progression or withdrawal from the study whichever occurs first. Subject may withdraw from the study at any time with or without reason. The investigator may withdraw any subjects from the study in the subject's best interest where continuation would place the subject in danger. If the investigator judges the subject is benefiting from study treatment after equivocal disease progression, study treatment may continue after discussion with the sponsor medical monitor. The decision to continue treatment beyond initial investigator-assessed progression and the subject's agreement should be documented in the study records.

6.2.6 Dose Modification

Every effort should be made to administer D-1553 at the planned dose and schedule (Table 6-2). In the event of significant toxicity, dosing may be delayed and/or reduced as described below. In the event of multiple toxicities, the dose modification should be based on the worst toxicity observed (according to the NCI CTCAE v5.0). Whenever possible, dose and scheduling modifications should be discussed between the investigator and the sponsor prior to implementation. Subjects are to be instructed to notify investigators at the first occurrence of any adverse symptom. After dose reduction for toxicity, the D-1553 dose for that subject will not be re-escalated.

Up to two dose reductions of D-1553 due to toxicity will be allowed before the subject must be permanently discontinued from the study. Any further dose reduction, if the investigator judges to be beneficial to the subject, must be discussed with the sponsor's medical director.

Table 6-2 Dose Modification Table

	Dose			
**Starting Dose Level**	600 mg QD	1200 mg QD	400 mg BID	600 mg BID
**Dose Reduction Level 1**	400 mg QD	400mg BID	600mg QD	400 mg BID
**Dose Reduction Level 2 (Daily)**		600 mg QD	400mg QD	600 mg QD

*This table is just for reference. The actual dose modification plan of each individual case should be discussed with

sponsor's medical monitor.

Dose modifications may occur:

- Within a cycle: dose interruption until adequate recovery during a given treatment cycle;
- Between cycles: next cycle administration may be postponed due to toxicity in the previous cycle.

6.2.7 Dose Interruptions/Delay/Reductions

Dose reductions of D-1553 may be required based on the worst toxicity experienced. In the event that a study drug related Grade 3 or higher toxicity occurs, defined in the NCI CTCAE v5.0, administration of D-1553 should be held until the drug related toxicity has resolved to grade 1 or baseline. Upon resolution of the toxicity or return to baseline, the subject should resume their current assigned dose of D-1553 or at the next lower dose level after discussion with sponsor. The following guidance can be used:

- After Cycle 1 Day 1, a treatment delay or dose interruption of more than one treatment cycle (21 or 28 days after the current cycle) will result in treatment discontinuation unless the subject is receiving clinical benefit as judged by the investigator and after discussion with the sponsor.
- Doses delayed or missed will not be made up (i.e., cycles will not be prolonged beyond the 21st or 28th calendar day in order to make up any missed doses).
- If a treatment interruption continues beyond Day 21 or Day 28 of the current cycle, then the day when treatment is resumed will be counted as Day 1 of the next cycle.

For subjects who experience a recurrent study drug related Grade 3 or higher toxicity, a discussion between the sponsor and investigator will occur to determine whether the subject should continue in the study. For any subject experiencing a Grade 3-4 toxicity, treatment should be resumed at next lower dose level after toxicity resolves to Grade 1 or baseline. A subject who experiences the same serious adverse event (SAE) of the same NCI CTCAE toxicity grade or higher related to D-1553 must discontinue the treatment immediately. Any subject with liver function test abnormalities meeting Hy's Law* criteria [redacted] must be permanently discontinued, [redacted]

* Hy's Law: ALT or AST ≥3 × ULN, TBIL ≥2 × ULN in the absence of cholestasis (serum alkaline phosphatase [ALP] >2 × ULN), and there are no other reasons to explain the combined elevation of aminotransferase and TBIL.

[redacted]

In combination cohorts, the combination drugs (other than D-1553) may have dose interruption, delay, reduction or other modifications according to their prescribing information.

[redacted]

[redacted]

[redacted]

[redacted]

[redacted]

[redacted]

[redacted]

[redacted]

[redacted]

[redacted]

[redacted]

[redacted]

[redacted]

[redacted]

[redacted] [redacted]

[redacted]

[redacted] [redacted]

[redacted]

[redacted] [redacted]

[redacted] [redacted]

[redacted] [redacted]

[redacted] [redacted]

[redacted]	[redacted]	[redacted]	[redacted]	[redacted]
[redacted]	[redacted]	[redacted]	[redacted] [redacted] [redacted] [redacted]	[redacted] [redacted] [redacted]
	[redacted]	[redacted]		

[redacted]	[redacted]	[redacted]	[redacted]	[redacted]<br>[redacted]<br>[redacted]
	[redacted]	[redacted]		
[redacted]	[redacted]	[redacted]	[redacted]	[redacted]
	[redacted]	[redacted]	[redacted]	
[redacted]	[redacted]	[redacted]	[redacted]<br>[redacted]	[redacted]

[redacted]	[redacted]	[redacted]	[redacted]	[redacted]
	[redacted]	[redacted]		
[redacted]	[redacted]	[redacted]	[redacted]	[redacted]
	[redacted]	[redacted]		
[redacted]	[redacted]	[redacted]	[redacted]	[redacted]
[redacted]	[redacted]	[redacted]	[redacted]	[redacted]
	[redacted]	[redacted]		
[redacted]	[redacted]	[redacted]	[redacted]	[redacted]
	[redacted]	[redacted]		
[redacted]	[redacted]	[redacted]	[redacted]	[redacted]
	[redacted]	[redacted]		
[redacted]	[redacted]	[redacted]	[redacted]	[redacted]
	[redacted]	[redacted]		
[redacted]	[redacted]	[redacted]	[redacted]	[redacted]
	[redacted]	[redacted]		
	[redacted]	[redacted]		

[redacted]

[redacted]

[redacted]

[redacted] [redacted]

[redacted]	[redacted]	[redacted]
[redacted]	[redacted]	[redacted]
[redacted]	[redacted]	[redacted]
[redacted]	[redacted]	[redacted]
[redacted]		

[redacted]

[redacted]

[redacted]

[redacted]

[redacted]

[redacted]

[redacted]

[redacted]

[redacted]

[redacted]

[redacted]

6.5 Cetuximab

Commercial cetuximab handling and storage must follow the instructions of the labeling.

https://www.accessdata.fda.gov/drugsatfda_docs/label/2019/125084s273lbl.pdf

6.5.1 Administration

Cetuximab will be administered IV weekly on Days 1, 8, and 15(±3 days) of every 21-day cycle at the study site according to institutional standards. The initial cetuximab dose (Cycle1 Day 1) is 400 mg/m^2 administered as a 120-minute IV infusion followed thereafter by a 250 mg/m^2 dose

administered as a 60-minute IV infusion. The infusion rate should be consistent with the local label but should not exceed 10 mg/min. Close monitoring is required during the infusion and for at least 1 hour after the end of the infusion. If an infusion reaction occurs while cetuximab is being administered, the infusion should be stopped immediately, and the patients should be closely monitored and treated in line with institutional standards. Any rechallenge with cetuximab following an infusion reaction should be first discussed with the Sponsor.

Premedications for routine cetuximab infusions may be used in accordance with the label and with the national and/or institutional standards, but preferably be based on a combination of an H1 antagonist (e.g., diphenhydramine) and dexamethasone (10 mg IV). Premedications should be administered approximately 30 minutes prior to cetuximab infusion. Following cetuximab label instructions, medications such as corticosteroids and antihistamines may be administered at the discretion of the Investigator to treat an existing infusion reaction, or as premedication for a patient who has previously experienced an infusion reaction.

Doses of cetuximab that are omitted for AEs or any other reason should not be made up.

6.5.2 Dose Modification

Recommended dose modifications for cetuximab based on the occurrence of cetuximab treatment-related AEs are summarized.

**Worst toxicity CTCAE, Grade**	**Dose Modification for Cetuximab During a Cycle of Therapy**
**Infusion Reaction**	If an infusion reaction occurs while cetuximab is being infused, the infusion should be stopped immediately and the patient should be evaluated.
Grade 1 or 2	Restart and complete the disrupted infusion at the discretion of the Investigator. The infusion must be restarted at a reduced rate. Additional pre-medications such as antihistamines or low-dose systemic corticosteroids may be administered when the infusion is restarted per institutional standards.<br>All subsequent infusions must also be administered at the reduced rate.
Grade 3 or 4	Permanently discontinue cetuximab
**Rash**	
Grade 1 or 2	Maintain dose level; consider initiating appropriate therapy (such as antihistamines, topical corticosteroids, and low-dose systemic corticosteroids)
Grade 3, despite therapy	Omit dose until resolved to ≤ Grade 2, then:<br>• If resolved in ≤ 7 days (or ≤ 14 days for acneiform rash), then maintain dose level<br>• If not resolved in ≤ 7 days despite appropriate skin toxicity therapy (or ≤ 14 days for acneiform rash), then permanently discontinue cetuximab
Grade 3 recurrent	Omit dose until resolved to ≤ Grade 2, then:<br>• If resolved in ≤ 7 days (or ≤ 14 days for acneiform rash), then decrease 1 dose level<br>• If not resolved in ≤ 7 days despite appropriate skin toxicity therapy (or ≤ 14 days for acneiform rash), then permanently discontinue cetuximab<br>• Permanently discontinue cetuximab after 3rd recurrence (upon 4th occurrence)
**Grade 4, despite skin toxicity therapy**	Permanently discontinue cetuximab

6.5.3 Adverse Reaction

The most common adverse reactions (incidence ≥25%) are: cutaneous adverse reactions (including rash, pruritus, and nail changes), headache, diarrhea, and infection. Please refer to the instructions of the cetuximab label for other adverse drug reactions.

[redacted]

6.7 Concomitant Medications/Treatments

Any prescription or over-the-counter (OTC) drug product other than study drugs should be discussed with the investigator to be sure there is no potential interference with study drugs, avoid their use during the study if possible. Medications or vaccinations specifically prohibited in the exclusion criteria are not allowed during the ongoing trial. No recreational or addictive drugs, herbal remedies, self-prescribed drugs, or excessive alcohol should be used during the study. If considered necessary for the subject's well-being, drugs for concomitant medical conditions or for symptom management may be given at the discretion of the investigator.

All concomitant medication will be recorded on the electronic case report form (eCRF) including, but not limited to, all prescription, OTC, herbal supplements and intravenous medications and fluids. If changes occur during the study, documentation of drug dosage, frequency, route and date will also be recorded on the eCRF. All concomitant medications received within 28 days prior to the first dose of study treatment and up to 30 days after the last dose of study intervention should be recorded. All concomitant medications administered during SAEs are to be recorded.

6.7.1 Prohibited Concomitant Therapy (during treatment and within 28 days after drug discontinuation)

Patients should not receive other anti-tumor therapy while on treatment in this study, i.e. surgery, radiotherapy therapy, cytotoxic, biological, Chinese herbal medicine with anti-tumor indication, or hormonal other than replacement. Exception can be made for localized palliative radiotherapy for symptom control provided that the localized palliative radiotherapy does not compromise tumor assessments of target lesions. Other anti-tumor treatments should not be administered until

disease progression (as per clinical practice standards at the study center), unmanageable toxicity or no further clinical benefit occurs which requires permanent discontinuation of D-1553.

Patients are not allowed to take the following medications, except for management of AEs as advised by the Principal Investigator. Patients must stop taking these drugs for at least 14 days before dosing, except coumarin anticoagulants that can be stopped one week before inclusion (heparin of low molecular weight is acceptable):

- Any strong inhibitor and inducer of CYP3A4 used 2 weeks before inclusion and during the trial treatment phases (e.g. ketoconazole, clarithromycin, indinavir, itraconazole, etc. refer to Appendix 4; however, this list may be not comprehensive, additional information may be obtained from the IFU of these drugs). In addition, herbals/supplements containing St. John's wart (*Hypericum perforatum* L.) and Sevillia orange etc. should also be avoided.
- [redacted]
- Any medications which can prolong QT interval or cause torsade de pointe (Refer to Appendix 4).

[redacted]

[redacted]

In the interest of patients' safety and acceptable standards of medical care, the Investigator will be permitted to prescribe the treatment(s) at his/her discretion.

6.7.2 Concomitant Therapy requiring caution during the study

Medications to be used with caution during treatment in this study (see Appendix 3) are listed below. These medications should be excluded if possible. If they must be given based on the investigator's judgment, then use with caution:

- Moderate inhibitors or inducers of CYP3A4
- [redacted]
- [redacted]
- [redacted]
- [redacted]
- Medications that carry a possible risk for QT prolongation

- Chinese herbal medicine or supplement

[redacted]

[redacted]

6.7.3 Permitted Concomitant Therapy during the study

The following therapies are permitted:

- Bisphosphonate
- Denosumab

[redacted]

- [redacted]
- [redacted]
- [redacted]
    - [redacted]
    - [redacted]
    - [redacted]
    - [redacted]
    - [redacted]
    - [redacted]
        - [redacted]
        - [redacted]
        - [redacted]

[redacted]

[redacted]

If, after assessment by the Investigator, therapeutic intent radiation for brain metastasis, therapy for bone metastasis or locoregional therapy e.g. local ablation, TACE, SIRT or arterial infusion chemotherapy could be initiated for the best benefit of the patient. The patient can start such therapy a minimum of 2 days after discontinuation of D-1553. Consequently, the patient will be censored for the primary endpoint analysis. D-1553 may be restarted 2 weeks after the completion of such treatment or when the patient has recovered from the side effects of such treatment.

The following medications/therapies may be given concomitantly under the following guidelines:

Hematologic Support

For Phase 1b and Phase 2, hematologic support may be administered as medically indicated (e.g., blood transfusions, granulocyte-stimulating factor (G-CSF), erythropoietin stimulating agents) according to the institutional site standard. If there are no standard procedures for the use of growth factors, the American Society of Clinical Oncology [ASCO] Guidelines for Use of Hematopoietic Colony-Stimulating Factors, available at www.asco.org, will be followed.

Management of Diarrhea

For Phase 1b and Phase 2, prophylactic treatment for diarrhea is permitted during the study if clinically indicated according to the institutional guidelines. If there are no institutional standards, refer to the guidelines published by Benson, et al. (39) in Journal of Clinical Oncology.

Management of Nausea/Vomiting

Antiemetics may be administered as clinical indicated. If there are no institutional standards, refer to the ASCO guidelines for Antiemetics in Oncology (40).

Effective Contraception

Combined (estrogen and progestogen containing) hormonal contraception associated with inhibition of ovulation:

- Oral
- Intravaginal
- Transdermal

Progestogen-only hormonal contraception associated with inhibition of ovulation:

- Oral

- Injection
- Implantable

Intrauterine device (IUD)

Intrauterine hormonal-releasing system (IUS)

Bilateral tubal occlusion

Vasectomized partner with documentation of the success of the vasectomy

Complete abstinence from heterosexual intercourse (periodic abstinence is not a safe method)

Male patients with partners who are WOCBP should use a combination of male condom with cap, diaphragm, or sponge with spermicide during the trial and for 6 months after the last dose of D-1553.

[redacted]

6.9 Compliance

When subjects are dosed at the site, they will receive study intervention directly from the investigator or designee, under medical supervision. The date and time of each dose administered in the clinic will be recorded in the source documents and recorded in the eCRF. The dose of study intervention and study subject identification will be confirmed at the time of dosing by a member of the study site staff other than the person administering the study intervention.

When subjects self-administer study intervention(s) at home, compliance with study intervention will be assessed at each visit. Subjects should record the dose and time of study intervention in a diary distributed by the study site. The diary will be reviewed by the study staff during the site visits and documented in the eCRF. Deviation(s) from the prescribed dosage regimen should be recorded in the eCRF.

A record of the number of D-1553 tablets [redacted] dispensed to and taken by each subject must be maintained and reconciled with study intervention and compliance

records. Intervention start and stop dates, including dates for intervention delays and/or dose reductions will also be recorded in the eCRF.

7 STUDY PROCEDURES

Study procedures and their time points are summarized in the Schedule of Assessments (see Table 1-1 through Table 1-3). Refer to the investigational product, laboratory and site imaging manuals for detailed collection and handling procedures.

Adherence to the study design requirements, including those specified in the Schedule of Assessments, is essential and required for study conduct.

A signed and dated IRB/IEC approved ICF must be obtained prior to performing any study specific procedures, including discontinuing standard therapy for observing study specific washout periods.

Subjects will be seen in the clinic for study evaluations. During all visits' invasive procedures like blood draws or biopsies should be completed after ECGs and vital signs.

Blood samples for biomarker and PK assessments should be drawn from a peripheral vein and not from a central venous catheter. The study specific lab manual will provide additional detail on lab sampling and handling requirements.

Furthermore, start of a treatment can be delayed for administrative/logistical reasons for up to 7 days to allow for appropriate scheduling after discussion with and final approval by sponsor.

Any missed visits, tests not done, or examinations that are not conducted must be reported as such on the eCRFs. Subsequent study visits should resume on the original schedule. Missed assessments at prior visits should not be duplicated at subsequent visits. Every effort should be taken to collect all biomarker and PK samples as described in the schedule of assessments.

Additional procedures deemed necessary as part of standard of care or as required by local laws and regulations may be performed at the Investigator's discretion.

At selected sites, up to a total of 30 phase 2 subjects will also be consented to have intensive PK and ECG data collected according to Table 1-6.

7.1 Phase 1 Procedures

7.1.1 Phase 1 - Screening Procedures (Day-28 To Day -1)

All subjects will be screened for eligibility and have a repeat brief pre-dose assessment conducted prior to the first dose of D-1553 to confirm eligibility. The following screening assessments must be performed within 28 days (unless otherwise noted) before enrollment:

- Written informed consent, must be obtained prior to undergoing any study procedure and may occur prior to the 28-day screening period
- Medical history review
- Diagnosis, history of solid tumor

- Demographics
- Physical examination, body weight and height
- Assessment against inclusion/exclusion criteria
- Pregnancy test which must be performed within 24 hours prior to initiation of first treatment. Pregnancy test is required for female subjects with childbearing potential.
- ECOG performance status
- Assessment of $KRAS^{G12C}$ status
- Vital signs, including temperature, pulse, respiratory rate and blood pressure
- Laboratory test (including hematology, blood chemistry, urinalysis, coagulation, etc.)
- ECG (12-lead) performed in a recumbent position after 5 minutes of rest, collected in triplicate and read by central laboratory
- [redacted]
- [redacted]
- [redacted]
- Tumor assessment (screening scans within 6 weeks prior to Day 1 of treatment cycle 1 are allowed, even if they are performed before the subject decides to join the study, to avoid radiation risk from extra scans)
- Prior/concomitant medication review
- Testing for HIV, Hep B and Hep C should be conducted during Screening for eligibility determination. A positive HBsAg will be considered positive for Hep B. A positive anti-HCV antibody and anti-HIV antibody will be considered positive for Hep C and HIV, respectively. [redacted]

7.1.2 Phase 1 – Pre-dose Assessment (Day-1 To Day 1)

The results from the screening visit evaluations will be reviewed to ensure the subject eligibility. The following procedures will be performed on Day -1 after check-in or the morning of Day 1 prior to dosing for all subjects:

- Reassessment of inclusion/exclusion criteria by review of all screening results and repeating any test if needed
- Physical examination, including body weight

- Vital signs, including temperature, pulse, respiratory rate and blood pressure
- Laboratory and pregnancy tests (tests performed in the screening period within 7 days of Day 1 do not need to be repeated on Day -1 or Day 1 unless clinically indicated). Pregnancy test is required for female subjects with childbearing potential
- [redacted]
- AE assessment
- Concomitant medication review

7.1.3 Phase 1 - Treatment (Day 1 of Treatment Cycle 1)

- ECOG performance status
- Vital signs
- 12-lead ECG
- [redacted]
- PK collection
- Study intervention
- Dispense study drug and instructions for dosing at home
- Dispense diary card
- AE assessment
- Concomitant medication review

7.1.4 Phase 1 - Day 2 of Treatment Cycle 1

- Vital signs
- PK collection
- Study intervention
- AE assessment
- Concomitant medication review

7.1.5 Phase 1b (Day 8 of Treatment Cycle 1)

- Laboratory assessments (including hematology, blood chemistry, urinalysis, coagulation etc.)

- Study intervention
- AE assessment
- Concomitant medication review

7.1.6 Phase 1 - Day 14 of Cycle 1

- Physical examination
- Vital signs
- Laboratory assessments (including hematology, blood chemistry, urinalysis, coagulation etc.)
- 12-lead ECG
- PK collection
- Study intervention
- AE assessment
- Concomitant medication review

7.1.7 Phase 1 - Day 1 of Treatment Cycle ≥ 2

- Physical examination
- ECOG performance status
- Vital signs
- Laboratory assessments (including hematology, blood chemistry, urinalysis, coagulation etc.)
- Pregnancy test (serum or urine) is required for female subjects with childbearing potential in odd-numbered cycles.
- 12-lead ECG
- [redacted]
- [redacted]
- [redacted]
- PK collection
- Study intervention
- Dispense study drug and instructions for dosing at home

- Dispense diary card
- AE assessment
- Concomitant medication review
- Tumor assessment at the completion of treatment cycle 2 (assessment performed at the completion of every 2 treatment cycles for the first 8 treatment cycles and at the completion of every 3 treatment cycles thereafter until disease progression, start of new anticancer treatment, death, withdrawal of consent, or until end of study)

7.1.8 Phase 1 - Upon Disease Progression or Unacceptable AE or Withdrawal from Study

The following procedures will be performed unless otherwise specified:

- Physical examination
- Pregnancy test is required for female subjects with childbearing potential
- ECOG performance status
- Vital signs
- Laboratory assessments (including hematology, blood chemistry, urinalysis, coagulation etc.)
- 12-lead ECG
- [redacted]
- [redacted]
- [redacted]
- Tumor assessment
- AE assessment
- Concomitant medication review

7.1.9 Phase 1 - Follow-Up

- AE assessment
- Concomitant medication review
- Tumor assessment will be performed every 12 weeks for subjects who do not have disease progression on study. Assessments will be continued until disease progression, death, lost to follow-up, start of another anticancer therapy or other reasons that a follow-up is not possible.

7.2 Phase 2 Procedures

During Phase 2, each of the 5 arms will apply a Simon 2-stage design with analyses after stage 1 to assess futility within the cohort before continuing into stage 2. The futility criteria are based on assumptions for the ORR for the SOC and the expected effect based on the addition of D-1553 to the SOC. Enrollment of subjects may have to be paused until valid ORR results are available for the respective number of subjects described in Section 4.1.3.

7.2.1 Phase 2 -Molecular Pre-screening of Tumor and ctDNA Samples for KRAS Alterations

Archived tumor tissue biopsy samples and ctDNA samples will be tested for KRAS^{G12C} mutation if not already tested. Patients can be enrolled and treated based on the following:

[redacted]

Subject with KRAS^{G12C} mutation in tumor or ctDNA samples local testing is acceptable for eligibility; all patients in Phase 2 study will have confirmation of KRAS^{G12C} mutation by Sponsor-designated central laboratory [redacted].

[redacted]

[redacted]

7.2.2 Phase 2 - Screening Procedures (Day -28 To Day -1)

All subjects will be screened for eligibility and a repeat brief assessment, referred to below as the pre-dose assessment, will be conducted prior to the first dose of D-1553 to confirm eligibility. The following screening assessments must be performed within 28 days (unless otherwise noted) before enrollment:

- Written informed consent, must be obtained prior to undergoing any study procedure and may occur prior to the 28-day screening period
- Medical history review
- Diagnosis, history of solid tumor
- Demographics
- Physical examination, body weight and height
- Assessment against inclusion/exclusion criteria
- Pregnancy test which must be performed within 24 hours prior to initiation of first treatment. Pregnancy test is required for female subjects with childbearing potential.
- ECOG performance status
- Assessment of $KRAS^{G12C}$ status
- Vital signs, including temperature, pulse, respiratory rate and blood pressure
- Laboratory assessment (including hematology, blood chemistry, urinalysis, coagulation, etc.)
- ECG (12-lead) performed in a recumbent position after 5 minutes of rest, collected in triplicate and read by central laboratory
- [redacted]
- LVEF measurement with Echocardiogram (only for Arm [redacted] F)
- [redacted]
- Tumor assessment (screening scans within 6 weeks prior to Day 1 of treatment cycle 1 are allowed, even if they are performed before the subject decides to join the study, to avoid radiation risk from extra scans)
- Concomitant medications

- [redacted]
- Archived tumor tissue (FFPE) for solid tumors
- Testing for HIV, Hep B and Hep C should be conducted during Screening for eligibility determination. A positive HBsAg will be considered positive for Hep B. A positive anti-HCV antibody and anti-HIV antibody will be considered positive for Hep C and HIV, respectively. [redacted]

7.2.3 Phase 2 – Pre-dose Assessment (Day -1 To Day 1)

The results from the screening visit evaluations will be reviewed to ensure the subject eligibility. The following procedures will be performed on Day -1 after check-in or the morning of Day 1 prior to dosing for all subjects:

- Reassessment of inclusion/exclusion criteria by review of all screening results and repeating any test if needed
- Physical examination, including body weight
- Vital signs, including temperature, pulse, respiratory rate and blood pressure
- Laboratory and pregnancy tests (these tests performed in the screening period within 7 days of Day 1 do not need to be repeated on Day -1 or Day 1 unless clinically indicated). Pregnancy test is required for female subjects with childbearing potential
- [redacted]
- AE assessment
- Concomitant medication review

7.2.4 Phase 2 -Treatment (Day 1 of Treatment Cycle 1)

- ECOG performance status
- Vital signs
- 12-lead ECG
- LVEF measurement with Echocardiogram (only for Arm [redacted] F)
- PK collection
- Study intervention
- Dispense study drug and instructions for dosing at home

- Dispense diary card
- AE assessment
- Concomitant medication review

7.2.5 Phase 2 –Arms [redacted] F (Day 2 of Treatment Cycle 1)

- Vital signs
- Study intervention
- AE assessment
- Concomitant medication review

7.2.6 Phase 2 – Arms [redacted] F (Day 8 of Treatment Cycle 1)

- Laboratory assessments (including hematology, blood chemistry, urinalysis, coagulation etc)
- Study intervention
- AE assessment
- Concomitant medication review

7.2.7 Phase 2 - Day 1 of Treatment Cycle ≥ 2

- Physical examination
- ECOG performance status
- Vital signs
- Laboratory assessments (including hematology, blood chemistry, urinalysis, coagulation etc.)
- Pregnancy test (serum or urine) is required for female subjects with childbearing potential in odd-numbered cycles.
- 12-lead ECG
- [redacted]
- LVEF measurement with Echocardiogram (only for Arm [redacted] F)
- [redacted]
- PK collection
- [redacted]

- Study intervention
- Dispense study drug and instructions for dosing at home
- Dispense diary card
- AE assessment
- Futility assessment (per arm according to Simon 2-stage design when stage 1 is completed for the respective arm)
- Concomitant medication review
- Tumor assessment at the completion of treatment cycle 2 (assessment performed every 2 treatment cycles for the first 8 treatment cycles and every 3 treatment cycles thereafter until disease progression, start of new anticancer treatment, death, withdrawal of consent, or until end of study.)

7.2.8 Phase 2 - Upon Disease Progression or Unacceptable AE or Withdrawal from Study

- Physical examination
- Pregnancy test is required for female subjects with childbearing potential
- ECOG performance status
- Vital signs
- Laboratory assessments (including hematology, chemistry, urinalysis, coagulation etc.)
- 12-lead ECG
- [redacted]
- [redacted]
- LVEF measurement with Echocardiogram (only for Arm [redacted] F)
- Tumor assessment
- AE assessment
- Concomitant medication review
- [redacted]

7.2.9 Phase 2 - Follow-Up

The following procedures will be performed for safety and efficacy (30 days after last dose) unless otherwise specified.

- AE assessment
- Concomitant medication review
- Tumor assessment will be performed every 12 weeks for subjects who do not have disease progression on study. Assessments will be continued until disease progression, death, lost to follow-up, start of another anticancer therapy or other reasons that a follow-up is not possible.
- Follow-up phone call every month to confirm OS

8 ADVERSE EVENT REPORTING

8.1 Adverse events

8.1.1 Definition of Adverse Events

An AE is any untoward medical occurrence in a study subject administered a product; the event need not necessarily have a causal relationship with the treatment or usage. An AE can therefore be any unfavorable and unintended sign (including an abnormal laboratory finding), symptom, or disease temporally associated with the use of an investigational product, whether or not considered related to the investigational product.

The time period for collecting AEs for each subject begins from the time the subject provides written informed consent, until the end of safety follow-up (30 calendar days after the last administration of investigational product or until the start of other anti-tumor therapy (see Section 8.2.4 for details).

Examples of AEs include but are not limited to:

- Aggravation of existing (prior to enrollment of study) medical conditions/ diseases (including exacerbation of symptoms, signs, laboratory abnormalities);
- Any new occurrence of AEs: any adverse medical conditions that newly occur (including symptoms, signs, newly diagnosed diseases);
- Abnormal laboratory test findings of clinical significance.

All AEs should be recorded in the AE page of the eCRF in details, including: event term, start and stop date, severity, seriousness, the causal relationship between the event and investigational drugs, actions taken with investigational product, as well as final results and outcomes.

8.1.2 Severity Assessment of Adverse Events

For severity of each AE, refer to the 5-grade scale developed from NCI-CTCAE v5.0. For AEs not included in NCI-CTCAE v5.0, the severity of each AE will be graded based on the general guidelines in Table 8-1.

Table 8-1 National Cancer Institute Common Terminology Criteria for Adverse Events Severity Grading

Grade	Clinical Description of Severity
1	Mild; asymptomatic or mild symptoms; clinical or diagnostic observations only; intervention not indicated.
2	Moderate; minimal, local or noninvasive intervention indicated; limiting age-appropriate instrumental ADL. Instrumental ADL refer to preparing meals, shopping for groceries or clothes, using the telephone, managing money, etc.
3	Severe or medically significant but not immediately life-threatening; hospitalization or prolongation of hospitalization indicated; disabling; limiting self-care ADL. Self-care ADL refer to bathing, dressing and undressing, feeding self, using the toilet, taking medications, and not bedridden.
4	Life-threatening consequences; urgent intervention indicated.
5	Death related to AE.

Abbreviations: AE=adverse event, ADL=Activities of Daily Living.

8.1.3 Causality Assessment of Adverse Events

An investigator's causality assessment is the determination of whether there exists a reasonable possibility that investigational product caused or contributed to an AE, such as whether the occurrence of AE follows a reasonable temporal sequence from administration of investigational product, the properties of investigational product, toxicological and pharmacological effects of investigational product, the use of concomitant medications, subjects' underlying diseases, medical history, family history and dechallenge and rechallenge reactions, etc. Generally, the facts (evidence) or arguments to suggest a causal relationship should be provided.

The causality of AE with investigational product administration will be assessed as "related", "probably related", "possibly related", "unlikely related" and "not related".

8.2 Serious adverse events

8.2.1 Definition of Serious Adverse Events

An SAE is any untoward medical occurrence at any dose of investigational product that:

- Results in death;
- Is life-threatening (refers to an event in which the subject was immediately at risk of death at the time of the event, and it does not refer to an event which hypothetically might have caused death if it was more severe);
- Requires inpatient hospitalization or prolongation of existing hospitalization;
- Results in persistent or significant disability/incapacity;
- Results in congenital anomaly/birth defect;
- Other medically significant event.

Medical and scientific judgment is exercised in determining whether SAE is appropriate in other situations, such as important medical event may not be immediately life-threatening or result in death or hospitalization but may jeopardize the patient or may require intervention to prevent one

of the other outcomes listed in the definition above. These should also usually be considered serious. Examples of such events are intensive treatment in an emergency room or at home for allergic bronchospasm; blood dyscrasias or convulsions that do not result in hospitalization; or development of drug dependency or drug abuse.

8.2.2 Hospitalization

Hospitalization is defined as any initial admission (even less than 24 hours) in a hospitalization, or any prolongation of an existing admission. Hospitalization does not include the following:

- Rehabilitation facilities;
- Nursing homes;
- General emergency admission;
- Same-day surgeries (as outpatient/same-day/ambulatory procedures)

Hospitalization or prolongation of hospitalization in the absence of a precipitating clinical AE is not in itself an SAE. Examples include:

- Admission for treatment of a preexisting condition not associated with the development of a new AE or with a worsening of the preexisting condition;
- Administrative admission (e.g., for yearly physical examination);
- Protocol-specified admission during a study (e.g., for a procedure required by the study protocol);
- Optional admission not associated with a precipitating clinical AE (e.g., for elective surgery);
- Preplanned treatments or surgical procedures. These should be noted in the baseline documentation for the entire protocol and/or for the individual subject;
- Admission exclusively for the administration of blood products.

Diagnostic or therapeutic noninvasive and invasive procedures, such as surgery, should not be reported as AEs. However, the medical condition for which the procedure was performed should be reported if it meets the definition of an AE. For example, an acute appendicitis that begins during the reporting period should be reported if the AE requirements are met, and the resulting appendectomy should be recorded as treatment of the AE.

8.2.3 Disease Progression and Death

Disease progression (PD) is defined as that the disease under study is progressing or worsening, including radiological progression and progression of clinical symptoms and signs. New metastases relative to the primary tumor and the progression of the original metastases are both considered to be PD. Events that are life-threatening, requiring inpatient hospitalization or prolongation of existing hospitalization, resulting in persistent or significant disability/incapacity,

due to symptoms and signs of PD, should not be reported as an SAE. If there is any uncertainty about whether an SAE is caused by PD, it should be reported as an SAE.

In the study population of this trial, "disease progression" is an expected situation and shall not be documented as AE. When disease progression occurs, events used to confirm disease progression shall be reported as AEs. For example, in patients with epilepsy which are determined to be related to brain metastases, the AE term "epilepsy" rather than "disease progression" or "brain metastasis" shall be documented.

All cases of death that occur within 30 calendar days after the last administration of investigational product must be reported as SAEs, regardless of whether it is assessed by the investigator as likely due to disease progression, or the subject has received any other anti-tumor therapy. The term "death" should not be reported as an SAE term, but rather as an outcome of an event. The cause of death, medical conditions/disease (including exacerbation of symptoms, signs) should be used to be recorded in the eCRF and reported as SAEs. If the cause of death is unknown at the time of report, it should be recorded as "unknown cause of death".

8.2.4 Other Anti-Tumor Therapy

If a subject begins any other anti-tumor therapy before the end of the safety follow-up period, only SAEs related to the investigational drug will be recorded after the start of such new anti-tumor therapies. Death during the safety follow-up period, regardless of whether the subject is receiving any other therapies, or whether the death is drug-related, must be reported in a timely manner.

8.2.5 SAE reporting requirements

The time period for collecting SAEs for each subject begins from the time the subject provides written informed consent, until [redacted] 30 calendar days after the last administration of other investigational product study treatment, whichever is earlier must be reported by the investigator.

All SAEs should be reported to InventisBio by the investigator on the "Serious Adverse Event or Adverse Event of Special Interest Report Form for Clinical Trial" (signed and dated) within 24 hours of awareness, regardless of whether this is an initial report or a follow-up report. The investigator should also report the SAEs to relevant organizations in a timely manner as required by local regulations.

The sponsor's email to receive safety reports (for SAEs, AESIs or exposure during pregnancy) in this study:

Email: pv2@inventisbio.com

SAEs occurring in a subject after the safety follow-up period has ended are reported to InventisBio if the investigator becomes aware of them; all SAEs that the investigator believes have at least a

reasonable possibility of being related to investigational product must be reported to InventisBio. The detailed record content of SAE should include symptoms, severity, causality with investigational product, time of onset, time of treatment, action taken with investigational product, follow-up time and method as well as outcome. If the investigator considers that a SAE is not related to investigational product while potentially related to the study conditions (e.g., termination of the original treatment or complications during the study), the relationship should be described in detail in the narrative section of SAE report form.

8.3 Adverse Event of Special Interest (AESI)

8.3.1 Overdose

For this study, an overdose of D-1553 is defined as any dose over 50% of assigned dose; [redacted] For other combination [redacted] agents [redacted], an overdose is defined as any dose over 50% of assigned dose. All overdose should be reported to InventisBio by the investigator on "Serious Adverse Event or Adverse Event of Special Interest Report Form for Clinical Trial" (signed and dated) within 24 hours of awareness (see Section 8.2.5 for safety reports contact details).

Any clinical symptoms/signs or abnormal laboratory results due to overdose of any study therapy should be reported as an adverse event.

No specific information is available on the treatment of overdose of D-1553 [redacted] In the event of overdose, the subject should be observed closely for signs of toxicity. Appropriate supportive treatment should be provided if clinically indicated.

8.3.2 Liver function test abnormalities

Abnormal values in AST and/or ALT concurrent with abnormal elevations of TBIL that meet all of the following 3 criteria in the absence of other etiologies should be reported as AESIs (Table 8-2). The Investigator should report to InventisBio on "Serious Adverse Event or Adverse Event of Special Interest Report Form for Clinical Trial" (signed and dated) within 24 hours of awareness (see Section 8.2.5 for safety reports contact details). If an AESI meets the SAE criteria, it should also be reported following the procedures for reporting SAE.

Table 8-2 Criteria for Liver Function Test Abnormalities

Criteria	Laboratory abnormalities
(1) Abnormal AST or ALT	• Subjects with AST and ALT baseline values within the normal range subsequently present with AST or ALT values ≥ 3 × ULN.<br>• Subjects with AST or ALT baseline values above the normal range subsequently present with AST or ALT values ≥ 2 times the baseline values and ≥ 3 × ULN, or with AST or ALT values ≥ 8 × ULN (whichever is smaller).
(2) Abnormal TBIL	• Subjects with TBIL baseline values within the normal range subsequently present with a TBIL value ≥ 2 × ULN;<br>• Subjects with TBIL baseline values above the normal range subsequently present with an increase of ≥ 1 × ULN in TBIL from baseline, or with a TBIL value ≥ 3 × ULN (whichever is smaller).
(3) No evidence of hemolysis, and ALP value < 2 × ULN or not available.	

Abbreviations: ALT=alanine aminotransferase, ALP=alkaline phosphatase, AST=aspartate aminotransferase, TBIL=total bilirubin, ULN= upper limit of normal.

Subjects who present with abnormal values in AST and/or ALT concurrent with abnormal elevations of TBIL during the treatment or follow-up period should return to the investigational site and be evaluated as soon as possible, preferably within 48 hours from awareness of the abnormal results. This evaluation should include laboratory tests, detailed history, and physical assessment. Besides repeated measurement of AST and ALT, the laboratory tests may also include albumin, creatine kinase, total bilirubin, direct bilirubin, GGT, PT/INR, and ALP. Detailed history, including relevant information, such as review of ethanol, acetaminophen, recreational drug, supplement (herbal) use and consumption, family history, sexual history, travel history, history of contact with a jaundiced person, surgery, blood transfusion, history of liver or allergic disease, and potential occupational exposure to chemicals, may be collected. Further testing for acute hepatitis A, B, C, D, and E infection and liver imaging (e.g., biliary tract) may be warranted. If a re-examination still shows consistency with above laboratory criteria and there are no other possible causes, the event shall be reported as AESI.

8.4 Pregnancy

D-1553 and D-1553 in combination with other therapies [redacted] may have adverse effects on a fetus in utero. Furthermore, it is not known if other therapies [redacted] has transient adverse effects on the composition of sperm.

Participants should be informed that taking the study medication may involve unknown risks to the fetus (unborn baby) if pregnancy were to occur during the study. In order to participate in the study, participants of childbearing potential must adhere to the contraception requirement from the day of study medication initiation (or 14 days prior to the initiation of study medication for oral contraception) throughout the study period up 6 months after the last dose of study medication. If there is any question that a participant of childbearing potential will not reliably comply with the requirements for contraception, that participant should not be entered into the study.

All pregnancies and exposure during breastfeeding, from the time of treatment/ allocation through 6 months following cessation of study treatment, or 30 days following cessation of study treatment if the participant initiates new anticancer therapy must be reported by the investigator.

If a female subject is pregnant during the study, the subject must discontinue investigational product administration immediately and withdraw from the study; if a male subject's partner becomes pregnant during the study, the male subject can continue the study. The investigator must report the pregnancy to InventisBio within 24 hours of awareness using the "Pregnancy Report Form" (see Section 8.2.5 for safety reports contact details), and to the relevant institutions in a timely manner per local requirement.

The investigator should follow up monthly on the pregnancy outcome (e.g., any early termination of pregnancy, or a live birth) until 1 month after delivery, and notify InventisBio and the ethics committee (or other organizations as required by local regulations) of the pregnancy outcome. If the pregnancy outcome meets the criteria for an SAE (i.e., ectopic pregnancy, spontaneous abortion, intrauterine fetal demise, neonatal death, or congenital anomaly), the investigator should follow the procedures for reporting SAEs.

If a subject experience an SAE during pregnancy, both the pregnancy and the SAE should be reported following the procedures for reporting SAE.

8.5 Collection and follow-up of AE/AESI/SAE

At each study visit, the investigator should assess whether any new AEs have occurred. All AEs and SAEs documented at a previous visit/contact and are designated as ongoing, will be reviewed at subsequent visits/contacts. And the investigator should provide the available follow-up information and resolve queries according to sponsor`s requirements in a timely manner.

Each AE must be monitored until the symptoms subside and any clinically relevant changes in laboratory values have returned to baseline and/or to Grade ≤ 1, or there is a satisfactory explanation for it (such as lost to follow-up, death). Every effort should be made to ensure that the subject achieves the best outcome and definite causality assessment is obtained.

9 DATA ANALYSIS/STATISTICAL METHODS

Detailed methodology for summary and statistical analyses of the data collected in this study will be documented in a Statistical Analysis Plan (SAP). This SAP will describe the analyses for each of the different phases of the study and provide results across the phases as appropriate. The SAP will be developed prior to the final analysis. Any modification of the predefined the analysis methodology will be described in the clinical study report.

9.1 Statistical Hypotheses

The Phase 2 part of the study will include Arms A to F. [redacted] Hypotheses will test within each cohort the null hypothesis that the true response probability is less than some uninteresting level P0 (poor), against the alternative hypothesis that the treatment effect is larger, [redacted]

9.2 Sample Size Determination

The total sample size of the study is anticipated at approximately 286 enrolled subjects. Based on whether earlier futility criteria are met in the Phase 2 arms, fewer subjects will be enrolled. However, in any of the arms a much higher effect is observed after stage 1 or stage 2, the protocol might be amended to further expand the population in the respective arm (see Section 4.1.3).

9.2.1 Phase 1a (Dose Escalation)

Approximately 27 subjects will be enrolled, assuming an average of 3 subjects per cohort and 6 subjects each at the highest dose cohort in the once-daily and twice-daily administered cohorts, in this 7-cohort dose escalation study.

9.2.2 Phase 1b (Dose Combination)

Subjects will be enrolled into 4 groups, each with approximately 9 subjects, assuming 2 dose levels are evaluated with up to 6 subjects at the highest dose group, except group 1. Approximately 13 subjects will be enrolled into group 1, assuming 2 dose levels are evaluated with up to 10 subjects at the highest dose group. A total of approximately 40 subjects will be enrolled in this 4-group dose combination study.

9.2.3 Phase 2

Subjects will be enrolled into 6 arms, [redacted] depending on the evaluation of effects at stage 1 and potential futility assessments. Arm B will enroll approximately 70 patients and Arm F will

enroll approximately 40 patients. A total of approximately 219 subjects will be enrolled in Phase 2 (see Section 4.1.3).

[redacted]

[redacted]

[redacted]

[redacted]	[redacted]
[redacted]	[redacted]
[redacted]	[redacted]
[redacted]	[redacted]
[redacted]	[redacted]

[redacted]

[redacted]

9.4 Statistical Analyses

The SAP will be developed and finalized prior to the final analysis. Below is a description of planned statistical analyses. Further details are presented in the SAP.

9.4.1 General Considerations

Descriptive statistics will be used throughout the study and described per dose level along with an overall assessment of the entire data. Descriptive analyses will present frequencies, percentages and 95% confidence intervals as well as summary measures like mean, median, minimum, maximum, standard deviations, standard errors and 95% confidence intervals.

9.4.2 Primary Endpoint(s)

For the Phase 1 portion, the primary endpoint will be the type, incidence, severity of AEs (graded by NCI CTCAE, v5.0), attribution and timing of AEs and the incidence of DLTs. Descriptive analyses will be performed for these outcomes (see Section 9.4.1).

9.4.3 Secondary Endpoint(s)

For the Phase 2 portion, ORR (CR + PR), DCR (CR + PR + SD), PFS and DOR, evaluated by RECIST, v1.1. Descriptive analyses will be performed on these endpoints (see Section 9.4.1). For time to event endpoints, DOR and PFS Kaplan-Meier analyses will be performed.

For the PK analyses the following analyses are planned: noncompartmental data analysis (determination of $t_{½}$, $AUC_{0\text{-}t}$, AUC_{inf}, MRT, V_{dss}/F, CL/F, C_{max}, t_{max} and C_{min}) will be performed on the individual plasma concentration-time data using actual times for D-1553 single agent [redacted] to address potential drug-drug interaction. Descriptive statistics will be performed on the plasma concentrations and on the PK parameters of D-1553 [redacted]

For the analysis of safety endpoints as secondary endpoints see Section 9.4.4.

Based on PK data obtained in this study as well as PK data obtained from other studies, a population PK analysis will be performed to characterize pharmacokinetic parameters (Clearance (CL), Volume of distribution (V)) and evaluate the effect of extrinsic and intrinsic factors to support proposed dosing regimen. Pharmacokinetic data will also be used to explore the exposure-response relationships for D-1553 antitumor activity/efficacy as well as safety in the proposed patient population, if feasible. The results of these analyses, if performed, will be reported separately.

9.4.4 Safety Analyses

Number and percentage of subjects with occurrence of DLT during the observation period will be provided by dose level and overall. Descriptive statistics will be provided for AEs (according to NCI CTCAE v5.0), and for ECG parameters, physical examination, performance status (ECOG), vital signs and laboratory tests. Results will be presented by dose level and overall.

9.4.5 Efficacy

The estimates of the objective response rate and disease control rate and their 95% exact confidence intervals by Clopper-Pearson method will be reported. The Kaplan-Meier method will be used for analyzing PFS, OS and the DOR; median PFS, median OS, median DOR, and corresponding 95% confidence intervals of the medians will be computed as appropriate. The Kaplan-Meier curves will be plotted. Descriptive statistics will be provided for efficacy results in Phase 1.

9.4.6 Other Analyses

Dose proportionality of D-1553 will be analyzed if data are sufficient. Pharmacokinetics of active metabolite(s) of D-1553 (if any) will be analyzed. PK analyses will be further described in the SAP.

9.5 Interim Analyses

No formal interim analyses are planned. There will be analyses of safety and PK (if available) data in Phase 1a and 1b during the dose escalation to decide escalation or de-escalation, and between Phase 1a and 1b to select D-1553 starting dose to be used in Phase 1b. [redacted]

9.6 Data Monitoring Committee

An SRC, [redacted] will regularly assess the safety of D-1553 administration throughout the clinical study and review the efficacy and safety data after each of the stage 1 results for a futility decision.

9.7 Protocol Deviations

Protocol deviations (missing assessments/visits) will be listed while those related to coronavirus disease 2019 (COVID-19) will be listed separately.

10 QUALITY CONTROL AND QUALITY ASSURANCE

The sponsor is responsible for implementing and maintaining quality assurance and quality control systems with written SOPs which is in compliance with ICH GCP and all applicable local regulations.

10.1 Monitoring of the Study

The clinical monitor (such as clinical research associate, CRA), a representative of the Sponsor, has the obligation to monitor the study closely. In doing so, the monitor will visit the investigator or sub-investigator and study facility periodically in addition to maintaining necessary contact through telephone, email, and letter. The monitor will maintain up to date information about the study through observation, review of study records and source documentation, and discussion of the conduct of the study with the investigator or sub-investigator and the staff. All aspects of the study will be carefully monitored by InventisBio or its designee for compliance with applicable government regulations, current GCP, and current standard operating procedures.

10.2 Audit

The sponsor or the third-party vendor delegated by the sponsor may conduct periodic audits of the study processes, including, but not limited to the study site visits, local laboratories, clinical database, and the final clinical study reports. When audits are conducted, access must be authorized for all study-related documents including medical history and concomitant medication documentation to authorized Sponsor's representatives.

10.3 Inspection

The regulatory authority may conduct inspection to the study site, the investigator shall authorize access of all study related documents to the regulatory authority.

10.4 Study Record Retention

Essential documents should be retained for at least 5 years after the last approval of a marketing application in an ICH region and until there are no pending or contemplated marketing applications in an ICH region or at least 2 years after the formal discontinuation of the clinical development of the investigational product. However, if it is required by the applicable regulations or by an agreement with the sponsor, these documents should be retained for a predefined/agreed period. It is the responsibility of InventisBio to inform the principal investigator or sub-investigator/institution as to when these documents are no longer needed to be retained.

11 DATA HANDLING AND RECORD KEEPING

11.1 Data Collection

An electronic data capturing as well as an information management system will be used. The system should combine all aspects of source data gathering with process control and clinical study management. All clinical and laboratory data should be collected and subjected to data entry, as appropriate.

The responsible study monitor will check the data during the monitoring visits. The investigator will ensure that the data collected are accurate, complete and legible. Data will be monitored against the source documents by the study monitor. Any changes made should be documented and signed.

11.2 Case Report Forms and Source Documents

All data obtained using paper collection methods during the clinical study will be recorded in the electronic data management system. All source documents should be placed in the subject's personal records/file.

The original electronic transferred data to the management system entries will be checked against source documents by the study monitor. Instances of missing or uninterpretable data will be discussed with the investigator for resolution.

11.3 Access to Source Documents

During the course of the clinical study, a study monitor will visit the sites to review protocol compliance, compare data entries and individual subject's personal records, assess drug accountability and ensure that the clinical study is being conducted according to pertinent regulatory requirements. Data entries will be verified against source documents. The review of medical records will be handled confidentially to ensure subject anonymity.

Checking of the data entries for completeness and clarity and verifying with source documents, will be required to monitor the clinical study for compliance with GCP and other regulations. Moreover, regulatory authorities of certain countries, IECs/IRBs can inspect/audit the site and the sponsor's clinical quality assurance group can conduct auditions. Direct access to source data will be required for these inspections and audits; data protection and subject confidentiality will be well observed.

11.4 Data Management

Standardized and validated procedures and systems will be used to collect, process and file the clinical data of this study. Any system used will be in compliance with the Food and Drug Administration (FDA) 21 CFR Part 11 requirements.

A data management plan will be prepared to describe the processes and data-flow within the clinical study. Timelines, versions for the electronic systems and coding will be defined in the plan, and if applicable, sponsor-specific requests will also be documented. The plan will be finalized before any dosing if possible, but before database lock.

A data validation specification will be created to outline the validation checks to be performed during the study and finalized. After the data has been monitored by the responsible study monitor, all data received will be reviewed, logged and filed.

The raw data intended for further processing will be checked by standard routines or according to the data validation specification. Queries will be generated and sent to the investigator for review and resolution. Corrections resulting from these queries will be confirmed on the data clarification forms. This process will be repeated until no further discrepancies are found. Applicable documentation will be stored in the study files.

Only trained study staff will have access to the clinical database and any change in the data will have a full audit trail.

12 ETHICS/PROTECTION OF THE HUMAN SUBJECTS

12.1 Ethical Conduct of the Study

The study will be conducted in accordance with ethical principles originating from the current GCP of the Declaration of Helsinki and are consistent with ICH.

12.2 Subject Data Protection

The ICF will incorporate with relevant data protection and privacy legislation.

All clinical study findings and documents are confidential. The investigator and members of the research team must not disclose such information without prior written approval from the sponsor.

The anonymity of the participating subjects must be maintained. Subjects will be specified in the electronic data management system and other documents by their subject number, not by name, date of birth or any other identifiable private information.

Documents that identify the subject (e.g., signed ICF) will be maintained in confidential by the investigator.

Study data will be stored in accordance with local and international data protection laws.

12.3 Ethics and Regulatory Review

The study will be submitted to the regulatory authority for review and approval, by the Contract Research Organization (CRO) in accordance with local regulatory procedures. The study will be submitted for ethical review and approval in accordance with local regulations.

12.4 Informed Consent

The subjects shall be informed of the nature, significance, implications and risks of the research study; and an informed consent will be presented and evidenced in writing, dated and signed, by the subject as evidence to indicate his/her acknowledgement of informed consent, prior to the start of the study.

The nature of the informed consent will be in compliance with the current Declaration of Helsinki, current GCP and local regulations.

12.5 Changes to the Clinical Study Protocol and Informed Consent Document

Study procedures will not be changed without mutual agreement of the investigator and the sponsor. If there are any substantial changes to the clinical study protocol, then these changes will be documented in a protocol amendment.

The amendment, if applicable, must be approved by the Regulatory Authority and the IRB before its implementation. Local requirements should be followed for revised clinical study protocols as well. If a protocol amendment requires a change to the ICF, the IRB should approve the revised

ICF before the revised document is used. Administrative changes will be communicated to the IRB, in accordance with local requirements.

13 RISK MANAGEMENT

Risk management will be implemented throughout the study. As this is a FIH study, dose escalation will be strictly managed and an SRC is set up to evaluate the safety and risk of the ongoing trial and determine the most appropriate way for dose escalation. AE trend will also be carefully monitored. Risk management process will be recorded and reported.

There is currently an outbreak of respiratory disease (COVID-19) caused by a novel SARS-CoV-2 that was first detected in Dec 2019. This new virus has rapidly spread across the globe causing the World Health Organization to declare a pandemic situation on March 12, 2020. The countermeasures initiated by national and local governments worldwide and the recommendations issued by the health authorities have impacted current and new clinical studies. As the threat of pandemic burden including new outbreaks, locally or globally, will impact the further conduct of clinical studies, appropriate risk assessments and mitigation measures will need to be taken into consideration in all clinical studies to protect subjects, site staff and society as a whole.

Both European Medicines Agency and FDA as well as national health authorities in Europe have issued new guidelines that aim to provide recommendations for actions for conduct of clinical studies of medical products during the COVID-19 pandemic (41, 42). Since the pandemic situation is evolving, guidelines, recommendations, national laws and local restrictions may change at high pace. Given the circumstances of potentially relapsing pandemic or epidemic situation with regard to the spread of COVID-19 in future, special attention will be paid to protect subjects participating in the study and site staff involved in the investigations against infection with SARS-CoV-2 as requested by the newly issued applicable local guidelines.

14 REFERENCES

1. Beganovic S. Clinical significance of the KRAS mutation. Bosn J Basic Med Sci. 2009;9 Suppl 1:17-20.

2. Hong DS. Targeting the KRAS G12C mutation in patients with advanced solid tumors. Clin Adv Hematol Oncol. 2019;17(11):612-4.

3. Biernacka A, Tsongalis PD, Peterson JD, de Abreu FB, Black CC, Gutmann EJ, et al. The potential utility of re-mining results of somatic mutation testing: KRAS status in lung adenocarcinoma. Cancer Genet. 2016;209(5):195-8.

4. Neumann J, Zeindl-Eberhart E, Kirchner T, Jung A. Frequency and type of KRAS mutations in routine diagnostic analysis of metastatic colorectal cancer. Pathol Res Pract. 2009;205(12):858-62.

5. [redacted]

17. [redacted]

[redacted]

25. Mendelsohn J, Baselga J. Epidermal growth factor receptor targeting in cancer. Semin Oncol. 2006;33(4):369-85.

26. Vermorken JB, Mesia R, Rivera F, Remenar E, Kawecki A, Rottey S, et al. Platinum-based chemotherapy plus cetuximab in head and neck cancer. N Engl J Med. 2008;359(11):1116-27.

27. Cunningham D, Humblet Y, Siena S, Khayat D, Bleiberg H, Santoro A, et al. Cetuximab monotherapy and cetuximab plus irinotecan in irinotecan-refractory metastatic colorectal cancer. N Engl J Med. 2004;351(4):337-45.

28. Sobrero AF, Maurel J, Fehrenbacher L, Scheithauer W, Abubakr YA, Lutz MP, et al. EPIC: phase III trial of cetuximab plus irinotecan after fluoropyrimidine and oxaliplatin failure in patients with metastatic colorectal cancer. J Clin Oncol. 2008;26(14):2311-9.

29. Krishnan G, D'Silva K, Al-Janadi A. Cetuximab-related tumor lysis syndrome in metastatic colon carcinoma. J Clin Oncol. 2008;26(14):2406-8.

30. Van Cutsem E, Köhne C-H, Láng I, Folprecht G, Nowacki MP, Cascinu S, et al. Cetuximab plus irinotecan, fluorouracil, and leucovorin as first-line treatment for metastatic colorectal cancer: updated analysis of overall survival according to tumor KRAS and BRAF mutation status. J Clin Oncol. 2011;29(15):2011-9.

31. Xue JY, Zhao Y, Aronowitz J, Mai TT, Vides A, Qeriqi B, et al. Rapid non-uniform adaptation to conformation-specific KRAS(G12C) inhibition. Nature. 2020;577(7790):421-5.

32. Adagrasib Data Create Buzz at ESMO. Cancer Discov. 2021.

33. Rizvi S, Gores GJ. Pathogenesis, diagnosis, and management of cholangiocarcinoma. Gastroenterology. 2013;145(6):1215-29.

34. [redacted]

39. Benson AB, Schrag D, Somerfield MR, Cohen AM, Figueredo AT, Flynn PJ, et al. American Society of Clinical Oncology recommendations on adjuvant chemotherapy for stage II colon cancer. J Clin Oncol. 2004;22(16):3408-19.

40. Basch E, Prestrud AA, Hesketh PJ, Kris MG, Somerfield MR, Lyman GH. Antiemetic Use in Oncology: Updated Guideline Recommendations from ASCO. American Society of Clinical Oncology Educational Book. 2012(32):532-40.

41. European Medicines Agency. Guidance on the Management of Clinical Trials during the Covid 19 (Coronavirus) Pandemic.Version 3. Mar 2020 [Available from: https://ec.europa.eu/health/sites/default/files/files/eudralex/vol-10/guidanceclinicaltrials_covid19_en.pdf.

42. Food and Drug Administration. Guidance on Conduct of Clinical Trials of Medical Products During Covid 19 Public Health Emergency. Jan 27, 2021 [Available from: https://www.fda.gov/regulatory-information/search-fda-guidance-documents/fda-guidance-conduct-clinical-trials-medical-products-during-covid-19-public-health-emergency.

15 APPENDIX

APPENDIX 1 ECOG PERFORMANCE STATUS

Grade	Performance
0	Fully active, able to carry on all pre-disease performance without restriction
1	Restricted in physically strenuous activity but ambulatory and able to carry out work of a light or sedentary nature, e.g., light housework, office work
2	Ambulatory and capable of all self-care but unable to carry out any work activities; up and about more than 50% of waking hours
3	Capable of only limited self-care; confined to bed or chair more than 50% of waking hours
4	Completely disabled; cannot carry on any self-care; totally confined to bed or chair
5	Dead

Oken M, Creech R, Tormey D, et al. Toxicity and response criteria of the Eastern Cooperative Oncology Group. Am J Clin Oncol. 1982;5:649-655.

[redacted] [redacted]

[redacted]	[redacted]	[redacted]	[redacted]
[redacted]	[redacted]	[redacted]	[redacted]

[redacted]

APPENDIX 3 LIST OF CONCOMITANT MEDICATIONS TO BE USED WITH CAUTION

[redacted]	[redacted]
[redacted]	[redacted]
[redacted]	[redacted]
[redacted]	[redacted]
[redacted]	[redacted]
[redacted]	[redacted]
[redacted]	[redacted]
[redacted]	[redacted]
[redacted]	

APPENDIX 4 PROHIBITED CONCOMITANT MEDICATION

[redacted]	[redacted]
[redacted]	[redacted]
[redacted]	[redacted]
[redacted]	[redacted]
[redacted]	[redacted]
[redacted]	[redacted]
[redacted]	
